# A critical role of the mechanosensor PIEZO1 in glucose-induced insulin secretion in pancreatic β-cells

**DOI:** 10.1038/s41467-022-31103-y

**Published:** 2022-07-22

**Authors:** Yingying Ye, Mohammad Barghouth, Haiqiang Dou, Cheng Luan, Yongzhi Wang, Alexandros Karagiannopoulos, Xiaoping Jiang, Ulrika Krus, Malin Fex, Quan Zhang, Lena Eliasson, Patrik Rorsman, Enming Zhang, Erik Renström

**Affiliations:** 1 Lund University Diabetes Centre, Department of Clinical Sciences Malmö, Unit of Islet Pathophysiology, Lund University, 20502 Malmö, Sweden; 2 Metabolic Research Unit, Institute of Neuroscience and Physiology, Department of Physiology, University of Göteborg, 405 30 Göteborg, Sweden; 3 Section for Surgery, Department of Clinical Sciences Malmö, Lund University, 20502 Malmö, Sweden; 4 Lund University Diabetes Centre, Department of Clinical Sciences Malmö, Unit of Islet Cell Exocytosis, Lund University, 20502 Malmö, Sweden; 5 Institution of Physical Science and Technology, Southwest University, Chongqing, 400715 China; 6 Lund University Diabetes Centre, Department of Clinical Sciences in Malmö, Unit of Molecular Metabolism, Malmö University Hospital, Lund University, 20502 Malmö, Sweden; 7 Oxford Centre for Diabetes, Endocrinology, and Metabolism, Radcliffe Department of Medicine, University of Oxford, Oxford, OX3 7LE UK

**Keywords:** Diabetes, Calcium signalling, Mechanotransduction

## Abstract

Glucose-induced insulin secretion depends on β-cell electrical activity. Inhibition of ATP-regulated potassium (K_ATP_) channels is a key event in this process. However, K_ATP_ channel closure alone is not sufficient to induce β-cell electrical activity; activation of a depolarizing membrane current is also required. Here we examine the role of the mechanosensor ion channel PIEZO1 in this process. Yoda1, a specific PIEZO1 agonist, activates a small membrane current and thereby triggers β-cell electrical activity with resultant stimulation of Ca^2+^-influx and insulin secretion. Conversely, the PIEZO1 antagonist GsMTx4 reduces glucose-induced Ca^2+^-signaling, electrical activity and insulin secretion. Yet, PIEZO1 expression is elevated in islets from human donors with type-2 diabetes (T2D) and a rodent T2D model (*db/db* mouse), in which insulin secretion is reduced. This paradox is resolved by our finding that PIEZO1 translocates from the plasmalemma into the nucleus (where it cannot influence the membrane potential of the β-cell) under experimental conditions emulating T2D (high glucose culture). β-cell-specific *Piezo1*-knockout mice show impaired glucose tolerance in vivo and reduced glucose-induced insulin secretion, β-cell electrical activity and Ca^2+^ elevation in vitro. These results implicate mechanotransduction and activation of PIEZO1, via intracellular accumulation of glucose metabolites, as an important physiological regulator of insulin secretion.

## Abstract (web summary)

Insulin secretion depends on action potential firing in pancreatic islet beta-cells, but the underlying mechanism is unclear. Here, the authors show that activation of the mechanosensor ion channel PIEZO1 plays a central role in beta-cell electrical activity and insulin release.

## Introduction

Defective insulin secretion, resulting in chronically elevated blood glucose levels, is a hallmark of all types of diabetes. The pancreatic islet is highly vascularized and dietary glucose reaches the pancreatic islet via circulation^1,2^. In the insulin-secreting β-cell, glucose initiates a series of reactions leading to the closure of ATP-regulated K^+^ channels (*K*_ATP_), initiation of electrical activity and activation of voltage-gated Ca^2+^ channels (VGCC), which culminate in stimulation of insulin secretion^3^. In vascular endothelial cells, mechanical shear forces induced by blood flow induce ATP release and trigger intracellular Ca^2+^ waves^4,5^. Regulation of internal islet blood flow by capillary pericytes is perturbed in islets from donors with type-2 diabetes (T2D)^6^. In endocrine β cells, high-glucose-induced hypertonicity results in swelling and stimulates insulin secretion by signal transduction pathways distinct from those that are secondary to glucose metabolism^7–9^.

PIEZO1 is a mechanosensitive ion channel involved in the regulation of a diverse range of physiological responses including (but not limited to) sense of touch, erythrocyte morphology and the development of the cardiovascular organogenesis^10–12^. PIEZO1 is a non-selective cation channel that allows passage of Ca^2+^, K^+^, and Na^+^ ions^13^. Under physiological conditions, opening of PIEZO1 therefore gives rise to an inward/depolarizing current^4,14–16^.

PIEZO1 is found both in the plasma membrane and the nucleus. Whereas plasmalemmal Piezo1 channels are influenced by membrane tension^17,18^, nuclear Piezo1 likely subserves functions other than mechanotransduction (such as control of gene transcription)^19^. Mechanotransduction might seem an unlikely mediator of metabolic sensing but yoda1, a PIEZO1 agonist, stimulates insulin secretion in insulin-secreting β-cell lines and rodent pancreatic islets^20^. Interestingly, an SNP linked to *PIEZO1* (rs9933309) is associated with long-term glycemic control in East Asians^21^. Epidemiological studies provide further evidence for an association between hypertension and abnormalities in insulin secretion^22,23^.

Here we have investigated the role of PIEZO1 in rodent and human pancreatic β cells. We found that PIEZO1 regulates β-cell electrical activity, intracellular Ca^2+^, insulin secretion, and gene expression. We propose that mechanosensing and PIEZO1 play a previously unrecognized role in β-cell function and systemic glucose metabolism and that this mechanism becomes defective in patients with type-2 diabetes.

## Results

### *PIEZO1* expression is increased in T2D islets

**Fig. 1:**
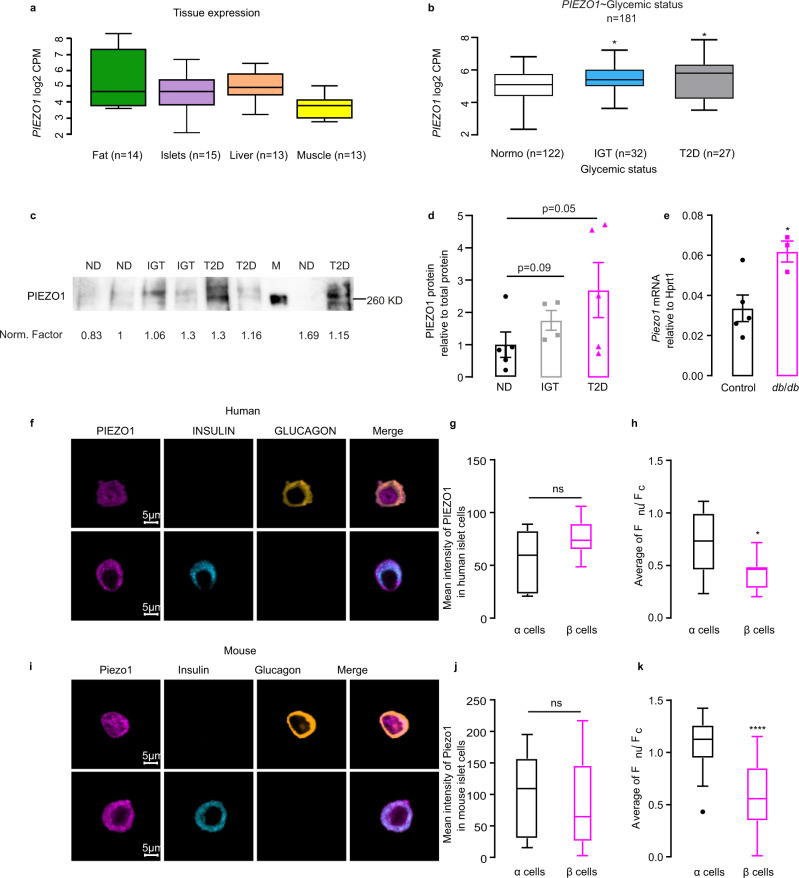
Piezo1 is highly expressed in diabetic islets. **a** RNA-seq data for *PIEZO1* in fat, pancreatic islets, liver and muscle from human tissues. CPM: count per million. **b** RNA-seq data for *PIEZO1* in samples of human islets from healthy donors (ND; *n* = 122), donors with impaired glucose tolerance (IGT; *n* = 32, *p* = 0.023) and donors with type 2 diabetic (T2D; *n* = 27, *p* = 0.015). **c** Immunoblot for PIEZO1 of human islets from ND (*n* = 5), IGT (*n* = 4) and T2D (*n* = 5) donors. **d** Densitometry quantification of PIEZO1 protein normalized to total protein in **c**, PIEZO1 expression in ND was set to 1. **e** *Piezo1* expression in mRNA level in C57BL/6J (Control; *n* = 5) and *db*/*db* mouse islets (*n* = 3). **f** Immunostaining for PIEZO1 (violet) in human islet α- and β cells. Glucagon (yellow) and insulin (light blue) served as the marker for α- and β cells, respectively. **g** Quantification of mean intensity of PIEZO1 in **f** (*n* = 7 α cells and 9 β cells). **h** The average ratio of fluorescence for PIEZO1 in the nuclear over that in cytosol calculated for **f** (*n* = 11 α cells and 11 β cells, *p* = 0.0109). **i** Same as in **f** but in dispersed mouse islets. **j** Quantification of mean intensity of PIEZO1 in **i** (*n* = 26 α cells and 30 β cells). **k**. The average ratio of fluorescence for PIEZO1 in the nuclear over that in cytosol calculated for **i** (*n* = 26 α cells and 30 β cells, *p* < 0.0001). Data are presented as dot plots with mean values ± SEM superimposed for figures **d**, **e** and box Tukey plot in **a**, **b**, **g**, **h**, and **j**, **k**. The minima and maxima of the box Tukey are the minimum and maximum data excluding the outliers, the center is the median, the bounds of box are the 25th (Q1) and 75th (Q3) percentiles, whiskers are the farthest points that are not outliers (i.e., that are within 3/2 times the interquatile range of Q1 and Q3). In **b**, statistical significances were evaluated by one-way ANOVA multiple comparisons. Unless otherwise indicated, statistical analysis was performed using a two-tailed Student’s *t*-test except for panel **d** where a one-tailed *t*-test was used. In **b**, **e**, **g**, **h**, **j**, **k**, *p < 0.05, ****p < 0.0001, ns not significant.

PIEZO1 is highly expressed in brain, lung, spleen and the urinary bladder^5,14,24^. RNA-sequencing (RNA-seq) in human tissues revealed expression of *PIEZO1* in pancreatic islets at levels comparable to those found in fat, liver, and muscle (Fig. 1a). Expression of *Piezo1* was also observed in mouse islets at about 10% the level in heart (Fig. S1a).

RNA-seq analysis in isolated human islets suggested that *PIEZO1* gene expression is significantly (*p* = 0.023) increased in donors with type-2 diabetes (T2D) and also the donors with impaired glucose tolerance (IGT) (*p* = 0.015) compared to levels found in normoglycemic donors (Fig. 1b). We confirmed this at the protein level: expression of the protein is increased by 150% in islets from patients with T2D relative to that observed in islets from non-diabetic donors (ND) (Fig. 1c, d). *Piezo1* expression was also increased by 100% in islets from diabetic *db*/*db* mice (Fig. 1e).

Pancreatic islet consists of several types of endocrine cell. Published RNA-seq data suggest low but detectable expression of *Piezo1* in both mouse and human α and β cells^25,26^. We corroborated this by confocal immunofluorescence imaging and Western blotting using a specific PIEZO1 antibody (see Methods). Expression of PIEZO1 was comparable in α and β cells in both human and mouse islet cells (Fig. 1f, g and i, j). Whereas localization of PIEZO1 was largely cytoplasmic/plasmalemmal in β cells, significant nuclear labeling was observed in α cells. We quantified this by calculating the nucleus/cytoplasm fluorescence ratio (*F*_nu_/*F*_c_). The ratio is significantly lower in β cells than in α cells in both human and mouse islets (Fig. 1h, k). The role of nuclear PIEZO1 in α cells is beyond the scope of this study, which focuses on its function in β cells, but we point out that α- and β cells are reciprocally regulated by glucose; this may contribute to the differential intracellular localization of PIEZO1 in the two cell types.

### Glucose-induced translocation of PIEZO1 into nuclei in both human and rodent β cells

**Fig. 2:**
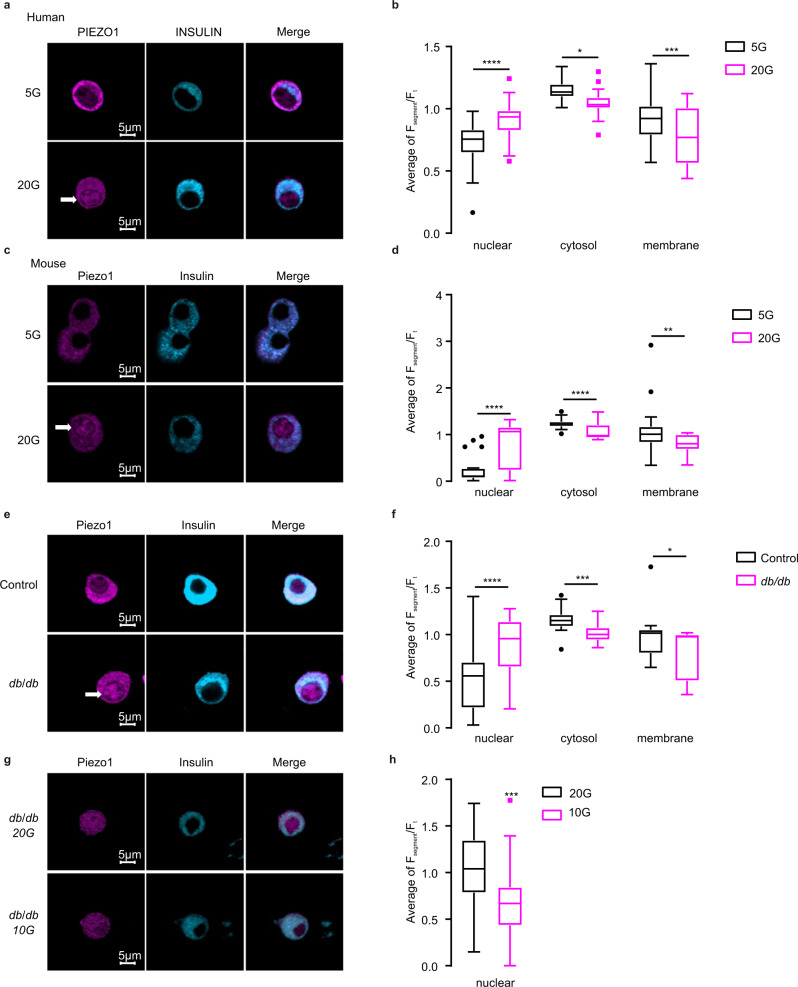
High glucose induces the translocation of PIEZO1 into the nucleus. **a** Immunostaining of PIEZO1 (violet) and Insulin (light blue) in human islet β cells incubated for a total of 96 h (72 h as intact islets and 24 h after dispersion into single cells) with 5 or 20 mM glucose (5 and 20G). **b** The average ratio of fluorescence for PIEZO1 in the nucleus, cytosol and membrane normalized to the total PIEZO1 fluorescence calculated for **a** (*n* = 34 and 38 cells for 5 and 20 mM glucose (5 and 20G), respectively), *p* values for the comparison between 5 and 20G for nuclear, cytosol or membrane are <0.0001, 0.0187, 0.0004, respectively. **c** Immunostaining of PIEZO1 (violet) and insulin (light blue) in mouse islet β cells incubated for 48 h with 5 or 20 mM glucose (5G and 20G). **d** As in **b** but for data in **c** (*n* = 24, 40 cells for 5 and 20 mM glucose (5 and 20G), respectively, *p* < 0.0001 for the comparison of fluorescence intensity in nuclear or cytosol with 5/20 mMG treatment, *p* = 0.0045 for that in membrane), *p* values for the comparison between 5 and 20G for nuclear, cytosol or membrane are <0.0001, <0.0001, 0.004, respectively. **e** Immunostaining of PIEZO1 (violet) and insulin (light blue) in C57BL/6J (Control) and *db*/*db* mouse islet β cells. Islets were dissociated into single cells immediately after isolation and cultured in standard medium (10 mM glucose) for 24 h prior to immunostaining. **f** As in **b** but for data in **e** (*n* = 21 and 23 cells for Control and *db*/*db*, respectively). **g** Immunostaining of PIEZO1 (violet) and insulin (light blue) in *db*/*db* mouse islet β cells cultured with 20 or 10 mM glucose for 72 h, *p* values for the comparison between 5 and 20G for nuclear, cytosol or membrane are <0.0001, 0.00017, 0.027, respectively. **h** The average ratio of fluorescence for PIEZO1 in the nucleus normalized to the total PIEZO1 fluorescence calculated for **g** (*n* = 23 and 22 cells for 20 and 10 mM glucose, respectively, *p* = 0.0009). Data are presented as box Tukey plot. The definition of box Tukey is as indicated as in Fig. 1. Statistical significances were evaluated by a two-tailed Student’s *t*-test in **b**, **d**, **f**, **h** *p < 0.05, **p < 0.01, ***p < 0.001, ****p < 0.0001, ns not significant.

Having established the presence of PIEZO1 in both mouse and human β cells, we next tested the effect of elevated glucose on its intracellular distribution. We found that exposing β cells to high glucose (20 mM) for 96 h promoted the intracellular translocation of PIEZO1 from the cytosol and plasma membrane into the nucleus in human (Fig. 2a, b) and mouse β cells (Fig. 2c, d and Fig. S1c). Thus, the intracellular distribution of PIEZO1 is under metabolic control.

In islets from hyperglycemic/diabetic *db*/*db* mice (fed plasma glucose: >25 mM), the proportion of PIEZO1 in the nucleus was also significantly higher than in β cells from normoglycemic control mice (Fig. 2e, f). Interestingly, PIEZO1 expression in *db*/*db* mouse β cells shifted from the nucleus to the cytosol/membrane following a subsequent 72-h incubation at 10 mM glucose compared to cells maintained at 20 mM throughout (Fig. 2g, h). In *db*/*db* islets incubated at 10 mM glucose, nuclear labeling was comparable to that in non-diabetic control β cells (Fig. 2f); note that 10 mM is close to normoglycemia in fed healthy mice^27^. We also compared the intracellular distribution of PIEZO1 in β cells from ND and T2D donors but observed no significant difference (Fig. S1d, e). This discrepancy between the mouse and human data we attribute to good glycemic control (unlike the untreated *db*/*db* mice) in patients with T2D. Indeed, measured HbA1c was not much elevated in donors with T2D compared to ‘control’ NDs (Supplemental Table 1). Collectively, these data indicate that the redistribution of PIEZO1 seen in diabetic *db/db* mouse islet β cells is a consequence of hyperglycemia and that T2D is associated with a reduction of plasmalemmal PIEZO1.

To determine which domain of PIEZO1 controls the intracellular trafficking, we cloned PIEZO1 fragments and studied the intracellular localization after transfection into INS-1 832/13 cells. PIEZO1 aa2189-2547-GFP (comprising the entity of the channel’s central pore) and aa2189-2458-GFP (corresponding to the outer helix of the pore) were confined to the cytoplasm regardless of the glucose concentration in the culture medium (Fig. S2c, d and f, g). By contrast, PIEZO1 aa2458–2547-GFP (the inner helix of the pore) exhibited both cytoplasmic and nuclear distribution and re-distributed into the cytosol in response to high glucose (Fig. S2e, h), echoing the behavior of wild-type PIEZO1. These results suggest that the C-terminal inner helix part of PIEZO1 (aa2458–2547) is required for the intracellular trafficking of PIEZO1. Additional experiments are required to identify the exact residue(s)/protein regions responsible for sensing the metabolic state and the translocation of PIEZO1 into the nucleus.

### PIEZO1 is important for swelling-induced insulin secretion

**Fig. 3:**
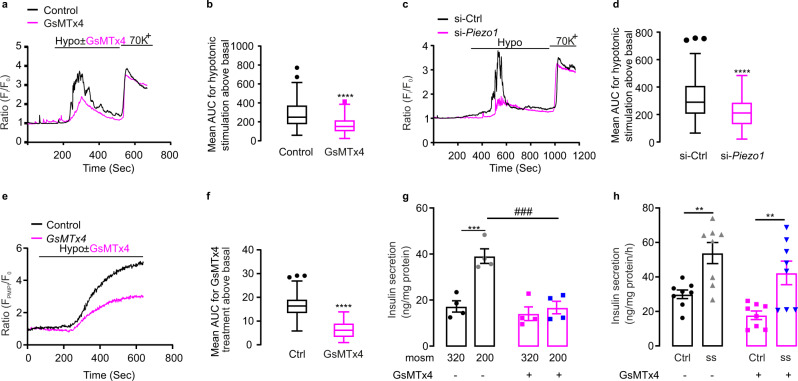
Piezo1 is important for swelling-induced insulin secretion. **a** [Ca^2+^]_i_ in INS-1 832/13 cells (measured using Fura-2 fluorescence; *F*_i_) normalized to the basal (*F*_0_) in cells superfused with 2.8 mM glucose before and during exposure to hypotonic solutions (‘hypo’) in the absence (black) or presence (red) of GsMTx4 followed by stimulation with 70 mM K^+^ as indicated. **b** Average of increase in AUC above basal for hypotonic stimulation with or without GsMTx4 treatment in **a** (*n* = 85 and 81 cells for Ctrl and GsMTx4 treatment, respectively, *p* < 0.0001). **c** As in **a** but in control cells (si-Ctrl) and after silencing of *Piezo1* (si-*Piezo1*) superfused as indicated. **d** As in **b** but in control cells (si-Ctrl) and after silencing of *Piezo1* (si-*Piezo1*) for **c** (*n* = 164 and 156 cells for control and si-*Piezo1*, respectively, *p* < 0.0001). **e** As in **a** but changes in membrane potential ∆ψ_p_ measured using PMPI. **f** Average of increase in AUC above basal for changes in ∆ψ_p_ induced by the superfusion medium with or without GsMTx4 for **e** (*n* = 71 and 62 cells for control and GsMTx4, respectively, *p* < 0.0001). **g** Insulin secretion in INS-1 832/13 cells in isotonic (~320 mOsm) or hypotonic buffer (~200 mOsm) with or without GsMTx4 (*n* = 4) as indicated, *p* values for the comparison between isotonic and hypotonic stimulation without/with GsMTx4 treatment are 0.0003, 0.7647, respectively, *p* value for the comparison between hypotonic stimulation without/with GsMTx4 is 0.0002. **h** Insulin secretion in INS-1 832/13 cells under shear stress (ss) with or without GsMTx4 in isotonic 2.8 mM glucose secretion buffer (*n* = 8), *p* values for the comparison between Ctrl and ss without/with GsMTx4 treatment are 0.0095, 0.0075, respectively. Data are presented as box Tukey plot in figures **b**, **d**, and **f**. In **g** and **h**, dot plots of individual experiments with mean values ± SEM are shown. The definition of box Tukey is as indicated as in Fig. 1. Evaluation of statistical significance was done using unpaired two-tailed Student’s *t*-test in figure **b**, **d**, and **f**, two-way ANOVA multiple comparisons in **g**. In **h**, statistical significances were evaluated by one-way ANOVA multiple comparisons. *, the significance compared between 2.8 mM and 16.7 mM glucose, #, the significance compared between treatments with and without pharmacological drugs. All statistical tests used were two-sided unless otherwise indicated. **p < 0.01, ***p < 0.001, ****p < 0.0001, ^###^*p* < 0.001.

Glucose-induced insulin secretion is triggered by membrane depolarization, electrical activity, activation of voltage-gated Ca^2+^ channels (VGCC) and elevation of [Ca^2+^]_i_^2,27^. We explored if activation of PIEZO1 in β cells leads to membrane depolarization using [Ca^2+^]_i_ imaging. We first examined Ca^2+^ signaling in INS-1 832/13 cells in response to swelling induced by hypotonic stimulation. This experimental paradigm emulates the situation following intracellular accumulation of glucose metabolites^28^. Hypotonicity led to robust increases in [Ca^2+^]_i_, in agreement with previous reports^9^, which was reduced by 50% in the presence of the Piezo inhibitor GsMTx4^29^ (Fig. 3a, b). Notably, GsMTx4 did not affect the increase in [Ca^2+^]_i_ produced by high extracellular K^+^ (Fig. 3a, b), a condition leading to membrane depolarization and opening of voltage-gated Ca^2+^ channels. The low expression of *Piezo2* is observed in mouse and human β cells^20,25^, makes it likely that the effect is principally (if not exclusively) mediated by activation of PIEZO1.

It has been reported that glucose-induced cell swelling coupled to insulin secretion involves SWELL1 (encoded by the gene *Lrrc8a*)^9^. We investigated this by measurements of hypotonicity-induced [Ca^2+^]_i_ increases and found that silencing of *Piezo1* or *Swell1* exerted similar but non-additive inhibitory effects (Fig. S3). Thus, PIEZO1 and SWELL1 operate in parallel.

To further investigate the effect of PIEZO1 in swelling-induced [Ca^2+^]_i_, we compared the hypotonicity-induced increases in [Ca^2+^]_i_ in control and *Piezo1*-deficient cells (Fig. 3c, d). Silencing of *Piezo1* led to >80% and ~50% reduction of mRNA and protein expression, respectively (Fig. S4). Genetic silencing of *Piezo1* produced effects similar to those observed with GsMTx4.

To confirm that activation of PIEZO1 depolarizes INS-1 832/13 cells, we measured the membrane potential using the plasma membrane potential indicator PMPI. Hypotonicity strongly depolarized the cells, an effect was partially reversed by the PIEZO1 inhibitor GsMTx4 (Fig. 3e, f).

We further tested whether PIEZO1 is involved in swelling-induced insulin secretion. INS-1 832/13 cells were exposed to isotonic or hypotonic solutions in the absence or presence of the blocker GsMTx4. GsMTx4 abolished insulin secretion evoked by hypotonicity (Fig. 3g).

PIEZO1 has been reported to be activated by shear stress^11,30,31^. Shear stress (induced by vigorous shaking of the test vials^20^) stimulated insulin secretion but this effect (unlike that produced by hypotonicity) persisted in the presence of GsMTx4 (Fig. 3h). Taken together, these data confirm that although β cells respond to both shear stress and hypotonicity with stimulation of insulin secretion only the latter effect involves activation of PIEZO1.

### PIEZO1 underlines glucose-induced cytosolic Ca^2+^ increases in β cells

**Fig. 4:**
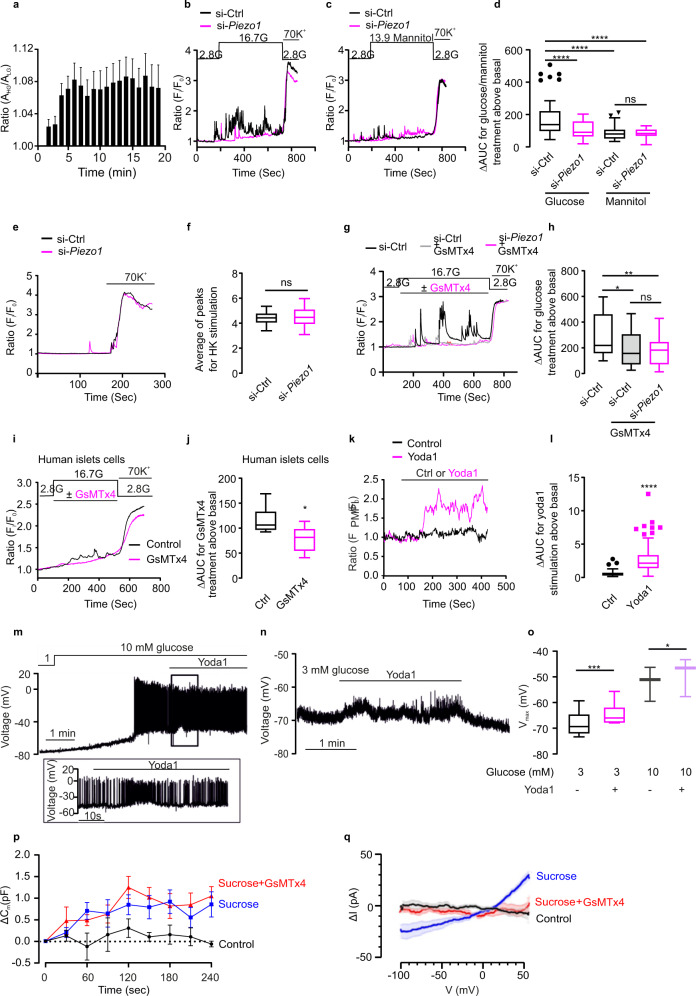
Piezo1 controls β-cell cytosolic Ca^2+^ homeostasis. **a** INS-1 832/13 cells were initially incubated at 2.8 mM glucose with the dye CFSE. The cells were superfused with medium containing 2.8 mM (*n* = 17) or 16.7 mM (*n* = 16) glucose while recording CFSE fluorescence. The cell area under 16.7 mM glucose relative to the cell size in cells that were maintained at 2.8 mM glucose throughout to compensate for dye bleaching. **b** [Ca^2+^]_i_ in INS-1 832/13 cells (measured using Fura-2 fluorescence; *F*_i_) normalized to the basal (*F*_0_) in cells superfused with medium containing 2.8 or 16.7 mM glucose in control cells (si-Ctrl; black) and after silencing of *Piezo1* (si-*Piezo1*, red) and following stimulation with 70 mM K^+^ as indicated. **c** As in **b** but 13.9 mM mannitol was added to the extracellular medium in the continued presence of 2.8 mM glucose as indicated. **d** Average of increase in AUC above basal (ΔAUC) in the presence of glucose (total: 16.7 mM) or mannitol (13.9 mM in the presence of 2.8 mM glucose) for **b** and **c** in control (si-Ctrl) cells and after silencing of *Piezo1* (si-*Piezo1*) (*n* = 54, 47, 41 and 31 cells, respectively, all the significant *p* values are <0.0001). **e** As in **b** but before and after increasing extracellular K^+^ ([K^+^]_o_) to 70 mM as indicated. **f** Peak *F*_i_/*F*_0_ above basal upon 70 mM K^+^ stimulation for **e** (*n* = 29 cells for si-Ctrl and si-*Piezo1*, respectively). **g** [Ca^2+^]_i_ in INS-1 832/13 cells (measured using Fura-2 fluorescence; *F*_i_) normalized to the basal (*F*_0_) in cells superfused with medium containing 2.8 or 16.7 mM glucose (G) in control cells (si-Ctrl) without (black) or with (gray) GsMTx4 and after silencing of *Piezo1* (si-*Piezo1*, red) with GsMTx4 and following stimulation with 70 mM K^+^ as indicated. **h** ΔAUC for the effects in response to glucose with or without GsMTx4 in control cells (si-Ctrl) or with GsMTx4 after silencing of *Piezo1* (si-*Piezo1*) induced increase in *F*_i_/*F*_0_ for **g** (*n* = 43, 31 and 34 cells, respectively, *p* values for the comparison between si-Ctrl and si-Ctrl/ si-*Piezo1* with GsMTx4 are 0.029, 0.0028, respectively). **i** [Ca^2+^]_i_ in human β cells superfused with medium containing 2.8 mM glucose or 16.7 mM glucose (G) in the absence (black) or presence (red) of GsMTx4 as indicated. The experiment was concluded by increasing [K^+^]_o_ to 70 mM. **j** ΔAUC for the effects of increasing glucose from 2.8 to 16.7 mM in the absence (black) or presence of GsMTx4 (red) for **i** (*n* = 7 and 8 cells for control and GsMTx4, respectively, *p* = 0.014). **k** Changes in membrane potential (∆ψ_p_) in INS-1 832/13 cells superfused with control (DMSO; black) or yoda1 (red trace) as indicated by horizontal lines. ∆ψ_p_ expressed as the PMPI fluorescence normalized to the initial value (*F*_PMPI_/F_0_) fluorescence **l** ΔAUC for changes in ∆ψ_p_ induced by addition of yoda1 (red) or solvent (DMSO, black) to superfusion medium for experiments of the type in **k** (*n* = 94 and 156 cells for control and yoda1, respectively, all the significant *p* values are <0.0001). **m** Recordings of electrical activity of β cells in intact pancreatic islets at 10 mM glucose, before and during application of 50 μM yoda1 (*n* = 6 cells in different islets). **n** As in **m**, but at 3 mM glucose. **o** The most negative membrane potential (*V*_max_) of β cells in the presence and absence of 50 μM yoda1 at 3 (*n* = 6 cells/islets) and 10 mM glucose (*n* = 3 cells/islets), p values for the comparison between yoda1 treatment under 3 mM G or 10 mM G are 0.0006, 0.0283. **p** Increase in cell capacitance (Δ*C*_m_) measured at various time points after establishment of the whole-cell configuration (*t* = 0 s) under control conditions and during intracellular application of sucrose with or without inclusion of GsMTx4 as indicated. *n* = 4, 5, and 5 cells for data control, sucrose and GsMTx4 treatment, respectively. **q** Net swelling-induced currents (ΔI) recorded during voltage ramps (100 ms) between −100 and +50 mV under control conditions (black; *n* = 4) and in the presence of 100 mM intracellular sucrose (blue; *n* = 5) and in the presence of intracellular sucrose and GsMTx4 (red; *n* = 5). Data are mean values (continuous lines) ± SEM (shaded areas). The swelling-induced current was isolated under each experimental conditions by subtracting currents recorded immediately after establishment of the whole-cell configuration from those observed after 4 min. Data are presented as box Tukey plot in figures **d**, **f**, **h**, **j**, **l**, and **o**. The definition of box Tukey is as indicated as in Fig. 1. Statistical significances were evaluated by one-way ANOVA multiple comparisons in **d** and **h**; unpaired Student’s *t*-test in **f**, **j**, and **l**, one-tailed *t*-test in **o**. All statistical tests used were two-sided unless otherwise indicated. *p < 0.05, **p < 0.01, ***p < 0.001, ****p < 0.0001, ns: not significant.

We confirmed that high glucose (from a basal 2.8–16.7 mM) induced β-cell swelling and increased cell area by >6% (Fig. 4a), in reasonable agreement with previous reports^9,32^. We explored whether swelling-induced activation of PIEZO1 plays a role in glucose-induced [Ca^2+^]_i_ signaling. To address this, we compared the effect of glucose and the non-metabolizable hexose mannitol (as an extracellular osmotic control) on [Ca^2+^]_i_. Unlike glucose, mannitol is not transported into β cells and it is not metabolized^33^. It is therefore not expected to increase the concentrations of intracellular osmolytes. High glucose (16.7 mM) elicited a series of [Ca^2+^]_i_ oscillations (Fig. 4b). Mannitol exerted no stimulatory effect beyond the spontaneous activity observed at basal (2.8 mM) glucose (Fig. 4c). Thus, swelling and ensuing β-cell excitation results from intracellular accumulation of glucose metabolites.

To investigate whether the effect of glucose reflects swelling-induced activation of PIEZO1, *Piezo1* was silenced by siRNA transfection. Silencing of *Piezo1* lowered the glucose-induced increase in [Ca^2+^]_i_ whilst not affecting [Ca^2+^]_i_ in the presence of mannitol (Fig. 4b–d). The response to membrane depolarization produced by high extracellular K^+^ (70 mM) was unaffected by silencing *Piezo1* (Fig. 4e, f). GsMTx4 inhibited the glucose-induced increases in [Ca^2+^]_i_ in control cells but had no additive inhibitory effect after silencing of *Piezo1* (Fig. 4g, h). Thus, GsMTx4 is a selective inhibitor of PIEZO1 in glucose-stimulated insulin-secreting cells.

GsMTx4 abolished glucose-induced [Ca^2+^]_i_ oscillations in human β cells whilst not affecting the increase produced by high-[K^+^]_o_ depolarization (Fig. 4i, j). Effects in rat islets were essentially identical to those in human islets (Fig. S5a, b).

We used the PIEZO1 activator yoda1^34^ to investigate the role of the channel in primary human β cells. We first verified the specificity of yoda1 by establishing that it was without effect on [Ca^2+^]_i_ after silencing of *Piezo1* but remained capable of increasing [Ca^2+^]_i_ in INS-1 832/13 cells lacking *Piezo2* (Fig. S6a–d). In human β cells (identified functionally by the glucose-induced increase in [Ca^2+^]_i_), yoda1 increased [Ca^2+^]_i_ when applied at 2.8 mM glucose (Supplementary Fig. S6e, f). The same effects of yoda1 were observed in primary rat β cells (Fig. S6g, h).

### Effects of yoda1 on glucose-induced electrical activity in β cells

We tested the effects of the PIEZO1 activator yoda1 on β-cell electrical activity. Using fluorimetric membrane potential measurements we found that application of yoda1 to INS-1 832/13 cells resulted in membrane depolarization (Fig. 4k, l), presumably by activating PIEZO1 channels^20^. We extended these observations to primary mouse β cells by electrophysiological measurements in intact islets^35^. The β cells were identified by functional fingerprinting as previously described^36^. Increasing glucose from 1 mM to 10 mM depolarized the β cell from −72 ± 6 mV to −52 ± 4 mV and action potential firing was initiated (Fig. 4m, o). Application of yoda1 consistently depolarized the interspike membrane potential but was otherwise had no obvious effects on electrical activity. We next tested yoda1 at a subthreshold glucose concentration (3 mM), which does not evoke electrical activity and insulin secretion itself. Addition of yoda1 (50 μM) again reversibly depolarized the β cell by 3 mV (Fig. 4n-o) but failed to generate regenerative action potential firing and induced low-amplitude (5–10 mV) membrane potential oscillations. We correlated the effects on β-cell electrical activity to changes in membrane conductance (determined after switching the amplifier from the current- to voltage-clamp mode). Yoda1 increased the membrane conductance (measured between −80 and −40 mV, a range of membrane potentials not associated with opening of voltage-gated ion channels) by 0.51 ± 0.23 nS (Fig. 4o, *n* = 12; *p* = 0.048 by paired Student’s *t*-test). Thus, the mode of action of yoda1 differs from that of the sulfonylureas that instead act by reducing the resting conductance (by inhibiting the K_ATP_ channels^37^).

We characterized the membrane currents induced by cell swelling in β cells in intact islets. Cell swelling was induced by supplementing the intracellular medium dialyzing the cell interior with 100 mM sucrose during standard whole-cell recordings (to mimic the effects of intracellular accumulation of metabolically derived osmolytes during glucose stimulation). We point out that this experimental paradigm is equivalent to extracellular hypotonicity. Cells infused with the standard (sucrose-free) intracellular medium were used as controls. Intracellular application of sucrose increased cell size (measured as cell capacitance, which is proportional to cell area) by 1.04 ± 0.27 pF over 4 min (*n* = 5; Fig. 4p), 13 ± 3% of the initial cell capacitance (7.3 ± 0.4 pF) and twice that produced by 16.7 mM glucose (see above). These experiments are performed at room temperature and with the intracellular Ca^2+^ concentration buffered to low levels using EGTA and the voltage-gated Ca^2+^ channels blocked with 1 mM Co^2+^, conditions associated with suppression of insulin secretion^38^ making it likely that the increase in capacitance reflects membrane unfolding/stretching^39^ rather than membrane insertion due to exocytosis of secretory granules^39^. No increase in cell capacitance was observed under control conditions (Fig. 4p, 0.1 ± 0.1 pF; *n* = 4). Inclusion of GsMTx4 in the extracellular medium did not prevent the sucrose-induced increase in cell capacitance (Fig. 4p, 1.3 ± 0.2 pF; *n* = 5).

The swelling-induced current was characterized by voltage ramps between −100 mV and +50 mV (Supplementary Fig. 7a). Current responses recorded under control conditions (no sucrose) and in the presence of sucrose with/without GsMTx4 immediately after establishment of the whole-cell configuration (≤30 s) were subtracted from those at later times to isolate the swelling-induced current (Supplementary Fig. 7b). There was a time-dependent increase in membrane currents induced by sucrose at both −100 and +50 mV that plateaued after 3–4 min. Under control conditions, the changes in membrane currents that occurred over 4 min were small (square). The experiments had to be performed at room temperature to allow the study of the impact of GsMTx4 at steady state (without perfusion) as the experiments would otherwise be prohibitively expensive.

Figure 4q shows current–voltage relationships of the net swelling-induced currents. Under control conditions, no time-dependent increases in membrane conductance were observed (−0.03 ± 0.02 nS relative initial membrane conductance; *n* = 8). In the presence of intracellular sucrose, an outwardly rectifying current developed with a reversal potential of 0 mV was observed. At negative membrane potentials, this current had a conductance of 0.19 ± 0.04 nS (*n* = 10; *p* < 0.0002 vs no sucrose). This current was abolished in the presence of GsMTx4 (2.5 μM) and the conductance change that occurred over 4 min averaged −0.03 ± 0.01 nS (*n* = 9), i.e. not different from that observed when cells were dialyzed with sucrose-free medium.

### PIEZO1 is required for glucose-stimulated insulin secretion in β cells

**Fig. 5:**
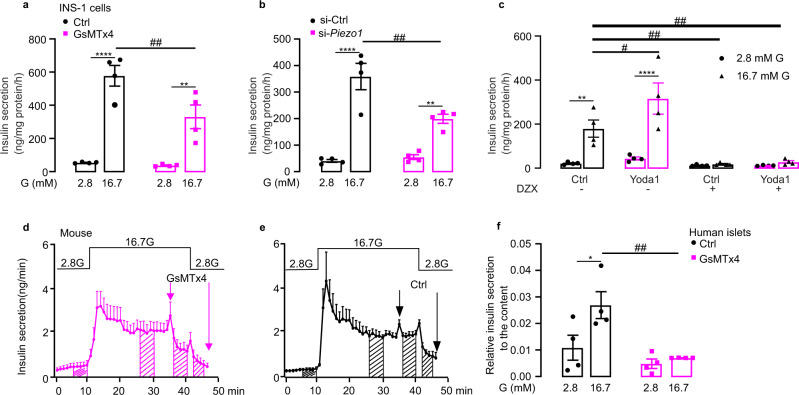
Piezo1 is required for glucose-stimulated insulin secretion in β cells. **a**. Insulin secretion in INS-1 832/13 cells at indicated glucose concentration in the absence (black) or presence (red) of GsMTx4 (2.5 μM) (*n* = 4), *p* values for the comparison between 2.8 and 16.7 mM G are <0.0001, 0.0018, *p* value for the comparison between Ctrl and GsMTx4 under 16.7 mM G is 0.0061. **b** As in **a** but in control cells (si-Ctrl) and after silencing of *Piezo1* (si-Piezo1) (*n* = 4), *p* values for the comparison between 2.8 and 16.7 mM G are <0.0001, 0.0051, *p* value for the comparison between si-Ctrl and si-*Piezo1* under 16.7 mM G is 0.0025. **c** Insulin secretion in INS-1 832/13 cells at indicated glucose concentration in the presence of DMSO (Ctrl), 25 µM yoda1, 200 µM diazoxide (DZX) or yoda1+DZX (*n* = 4), *p* values for the comparison between 2.8 and 16.7 mM G under Ctrl or yoda1 treatment are 0.0028, <0.0001, *p* values for the comparison between Ctrl and yoda1, Ctrl+DZX, yoda1+DZX under 16.7 mM G are 0.0155, 0.0035, 0.0062, respectively. **d** The average curve of insulin release measured by in situ pancreas perfusion with GsMTx4 from t=35 min to t=46 min as indicated (GsMTx4; *n* = 4). **e** As in **d** but perfuse without GsMTx4 (Ctrl; *n* = 3). **f** As in **a** but in human islets (*n* = 4), *p* value for the comparison between 2.8 and 16.7 mM G in Ctrl is 0.015, that for the comparison between Ctrl and GsMTx4 under 16.7 mM G is 0.0039. Data are presented as dot plots with mean values ± SEM. Statistical significances were evaluated by two-way ANOVA multiple comparisons in **a**, **b**, **c**, **f**. *, the significance compared between 2.8 mM and 16.7 mM glucose, #, the significance compared between treatments with and without pharmacological drugs. All statistical tests used were two-sided unless otherwise indicated. *p < 0.05, **p < 0.01, ****p < 0.0001, ^#^*p* <0.05, ^##^*p* < 0.01, ns not significant.

The results above demonstrate that PIEZO1 plays a key role in the β cell’s responsiveness to (glucose-induced) cell swelling. Indeed, GsMTx4 inhibited glucose-induced insulin secretion by 50% (Fig. 5a), comparable to the inhibitory effect of silencing of *Piezo1* (Fig. 5b). *Piezo1* silencing failed to affect insulin secretion evoked by high-K^+^ stimulation (Fig. S8a), in agreement with the [Ca^2+^]_i_ imaging data (Fig. 4e, f). In freshly isolated rat islets, both GsMTx4 (Fig. S8b) and ruthenium red (RR) (Fig. S8c), a non-selective inhibitor of PIEZO1, reduced glucose-stimulated insulin secretion. Similar effects were observed in human islets (Fig. S8d) and INS-1 832/13 cells (Fig. S8e).

The Piezo1 agonist yoda1 stimulated glucose-induced insulin secretion in INS-1 832/13 cells (Fig. 5c). We compared the stimulatory effects of yoda1 in the absence and presence of the K_ATP_ channel opener diazoxide (200 μM). Diazoxide alone decreased basal and high-glucose-stimulated insulin secretion by 50% and 90%, respectively (Fig. 5c). The stimulatory effect of yoda1 on insulin secretion was abolished by diazoxide (Fig. 5c). Yoda1 was also without effect on insulin secretion in INS-1 832/13 cells depolarized with 50 mM K^+^ regardless of whether the experiments were performed in the absence or presence of diazoxide (Fig. S8f).

We used the perfused mouse pancreas to determine the effect of GsMTx4 on insulin secretion under conditions that are close to the in vivo situation. Elevating glucose from 2.8 to 16.7 mM evoked a biphasic stimulation of insulin secretion (Fig. 5d, e). The inhibitor was added during steady state 2nd phase insulin secretion. To quantify the effect, we compared insulin secretion before and after addition of the inhibitor. Data were quantified during the indicated intervals and the data are summarized in Fig. S8g, h. When applied at steady state, GsMTx4 inhibited glucose-induced insulin secretion by 36% (Fig. 5d and Fig. S8g). Insulin secretion promptly decreased to the prestimulatory level both in the absence and presence of GsMTx4 when glucose was subsequently returned to the basal 2.8 mM. Control experiments were done to ascertain that simply switching the medium did not affect insulin secretion (Fig. 5e and Fig. S8h).

We extended these data to human islets using the PIEZO1 inhibitor GsMTx4. In static incubations, the inhibitor abolished glucose-stimulated insulin secretion in human islets (Fig. 5f). These data suggest that PIEZO1 plays an important role in glucose-induced insulin secretion.

### Impairment of glucose-induced insulin secretion and β-cell electrical activity in β-cell-specific *Piezo1*-knockout mice

**Fig. 6:**
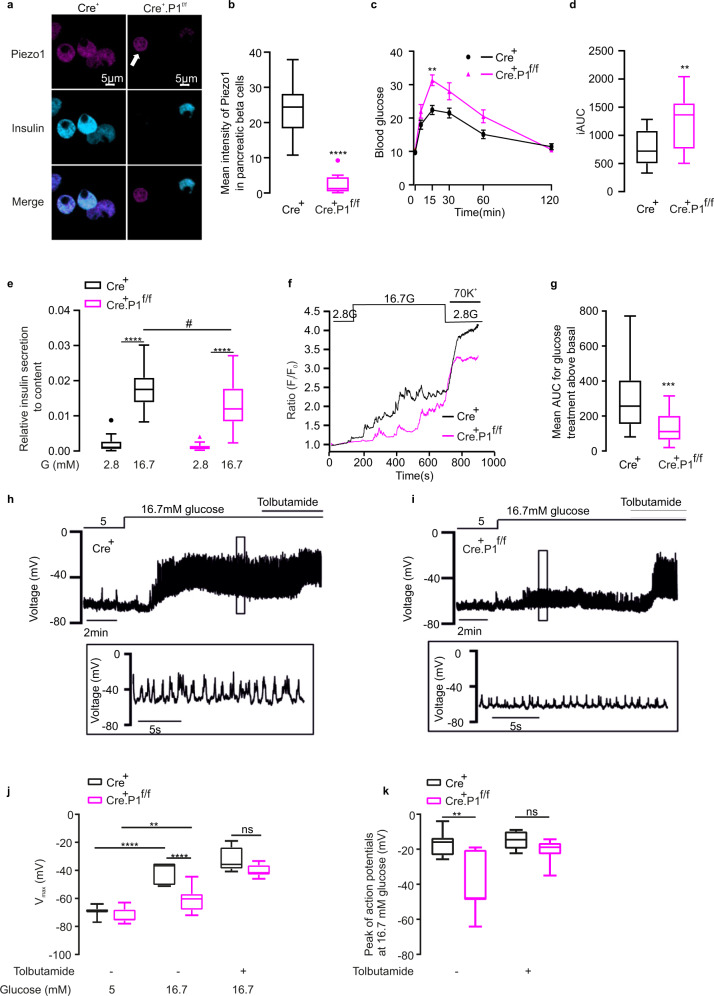
Impairment of glucose-induced Ca^2+^ homeostasis in β-cell-specific *Piezo1*-knockout mice. **a** Immunostaining of PIEZO1(violet), Insulin (light blue) in dispersed RIP-Cre^+^, Cre^+^.P1^f/f^ mouse islets, white arrow points to the non- β cells. **b** The mean intensity of PIEZO1 in β cells for **a** (*n* = 28, 12 cells for RIP-Cre^+^, Cre^+^.P1^f/f^, respectively, *p* < 0.0001). **c** Blood glucose post-IPGTT in male RIP-Cre^+^ (*n* = 9, black) or Cre^+^.P1^f/f^ mice (*n* = 9, red), *p* = 0.004. **d** The average of the incremental area under the curve (iAUC) from 0 to 120 min for **c** in male RIP-Cre^+^ (black) or Cre^+^.P1^f/f^ mice (red) (*n* = 9 and 9, respectively, *p* = 0.027). **e** Insulin secretion in isolated RIP-Cre^+^ (*n* = 15, 20 for 2.8 or 16.7 mM G, respectively, black) or Cre^+^.P1^f/f^ mouse (*n* = 15, 19 for 2.8 or 16.7 mM G, respectively, red) islets, the results were achieved from 4 mice in each group, *p* values for the comparison between 2.8 and 16.7 mM G in RIP-Cre^+^, Cre^+^.P1^f/f^ mouse were <0.0001, and that for the comparison between RIP-Cre^+^ and Cre^+^.P1^f/f^ under 16.7 mM G was 0.013. **f** [Ca^2+^]_i_ in RIP-Cre^+^, Cre^+^. P1^f/f^ mouse islet cells superfused with medium containing 2.8 or 16.7 mM glucose and following stimulation with 70 mM K^+^ as indicated. Data expressed as fura-2 fluorescence at each time point normalized to initial value (*F*_*i*_/*F*_0_). **g** Average of area under the curve (AUC) above basal for 16.7 mM glucose-induced increase in *F*_i_/*F*_0_ for experiments in **f** (*n* = 33 and 20 for RIP-Cre^+^, and Cre^+^.P1^f/f^, respectively), *p* = 0.0002. **h**, **i** Representative recordings of electrical activity of β cells in intact pancreatic islets from control (Cre^+^; **h** and *Piezo1*-knockout (Cre^+^.P1^f/f^; **i** mice perfused with 5 mM and 16.7 mM glucose (*n* = 8 for control and *n* = 10 for knockout islets). Tolbutamide was added to superfusion medium as indicated. **j** Most negative membrane potential at 5 (*n* = 8, 10 cells for Cre^+^, Cre^+^.P1^f/f^, respectively, *p* < 0.0001) and 16.7 mM (*n* = 8, 10 cells for Cre^+^, Cre^+^.P1^f/f^, respectively, *p* < 0.0001) glucose and 100 μM tolbutamide (*n* = 6, 10 cells for Cre^+^, Cre^+^.P1^f/f^, respectively) in control and *Piezo1*-deficient cells, *p* value for the comparison between 5 and 16.7 mM G in Cre^+^.P1^f/f^ mice was 0.0033. **k** Averaged action potential peak voltage at 16.7 mM glucose and in the presence (*n* = 6, 10 cells for Cre^+^, Cre^+^.P1^f/f^, respectively) or absence (*n* = 8, 8 cells for Cre^+^, Cre^+^.P1^f/f^, respectively, *p* = 0.0001) of tolbutamide as indicated in control and *Piezo1*-deficient cells. Data are presented as box Tukey plot for figures **b**, **d**, **e**, **g**, **j**, and **k**. The definition of box Tukey is as indicated as in Fig. 1. Statistical significances were evaluated by two-way ANOVA multiple comparisons in **c**, **e**, **j**, and **k**; unpaired *t*-test was used in **b**, **d**, **g**. All statistical tests used were two-sided unless otherwise indicated. **p < 0.01, ***p < 0.001, ****p < 0.0001, ^#^*p* < 0.05.

To explore the systemic role of PIEZO1-dependent insulin secretion, we generated β-cell-specific Piezo1-knockout mice (Fig. S9a and b). The phenotype of the RIP-Cre mice used here is indistinguishable from that of wild-type C57BL/6 mice^40^. Blood glucose excursions after IPGTT between RIP-cre and floxed *Piezo1* mice were not different (Fig. S9c). We confirmed the successful deletion of *Piezo1* in β cells, real-time qPCR was performed using primers targeting exons 21-22 (Fig. S9d) or exons 22–23 (Fig. S9e) in isolated islets from RIP-Cre, heterozygous (Cre^+^.P1^f/+^) and homozygous (Cre^+^.P1^f/f^) *Piezo1*-knockout mice. *Piezo1* expression was reduced by ~50% in Cre^+^.P1^f/f^ mice. Immunostaining of dispersed mouse islet cells confirmed the successful deletion of *Piezo1* in β cells in homozygous (Cre^+^.P1^f/f^) *Piezo1-*knockout mice (Fig. 6a, b). As expected, the non-β cells retained PIEZO1 immunoreactivity (Fig. 6a, b). This accounts for the residual *Piezo1* expression detected in mouse islets after β-cell-specific ablation of the gene.

Compared to control RIP-Cre (Cre^+^), β-cell-specific *Piezo1*-knockout mice (Cre^+^.P1^f/f^) had impaired glucose tolerance and blood glucose 15 min after the glucose bolus as well as the incremental AUC over 120 min were increased by ~100% (Fig. 6c, d).

We compared glucose-induced insulin secretion in isolated islets from Rip-Cre control and *Piezo1-*knockout mice. Basal insulin secretion at 2.8 mM glucose was not different between control and *Piezo1*-knockout mice. However, glucose-induced insulin secretion was reduced by ~50% (Fig. 6e), explaining the impaired glucose tolerance in these mice.

We measured [Ca^2+^]_i_ in dispersed β cells from control Rip-Cre and *Piezo1*-knockout mice to explore whether the reduction of glucose-induced insulin secretion was attributable to lowered capacity of glucose to increase [Ca^2+^]_i_. Although *Piezo1*-deficient β cells retained some glucose responsiveness, the increase in [Ca^2+^]_i_ in response to high glucose was reduced by >65% compared to control cells (Fig. 6f, g).

We applied perforated patch whole-cell measurements of electrical activity in β cells of control and *Piezo1*-knockout mice^35^ (Fig. 6h, i). In control islets, increasing glucose from 5 mM to 16.7 mM depolarized the β-cell membrane potential from −69 ± 1 mV to −42 ± 3 mV (*n* = 8; Fig. 6j) and initiated action potential firing with a frequency of 2.2 ± 0.4 Hz. In *Piezo1*-knockout islets, increasing glucose from 5 mM to 16.7 mM likewise produced membrane depolarization but the effect was weaker than in control β cells; from −72 ± 2 mV at 5 mM glucose to −61 ± 3 mV at 16.7 mM glucose (*n* = 10; Fig. 6i). Glucose-induced action potential firing was variable; no regenerative electrical activity was observed in 2 of the 10 cells and in the remaining 8 β cells the amplitude was lower. On average, the action potentials peaked at −17 ± 2 mV (*n* = 8, Fig. 6k) in control cells and −40 ± 6 mV in the *Piezo1*-deficient cells in which electrical activity was observed (*n* = 8, Fig. 6k). In addition, the firing frequency was reduced and averaged 1.0 ± 0.4 Hz (*n* = 10; *p* < 0.04 vs control cells). *Piezo1*-deficient β cells retained full responsiveness to 100 μM tolbutamide, which depolarized the β cells to −40 ± 1 mV (*n* = 10), similar as the −34 ± 4 mV (*n* = 6) in control β cells (Fig. 6h–j). Even in the presence of tolbutamide the amplitude of the action potentials observed in the *Piezo1*-deficient β cells tended to be lower than in the control cells but this difference was not statistically significant (Fig. 6k).

Two important conclusions can be drawn from these measurements. First, PIEZO1 is required for glucose-induced electrical activity. Second, the finding that tolbutamide depolarizes β cells in control and *Piezo1-*knockout β cells to the same extent suggests the existence of an additional depolarizing background conductance.

### Regulation of Piezo1 on global gene expression

**Fig. 7:**
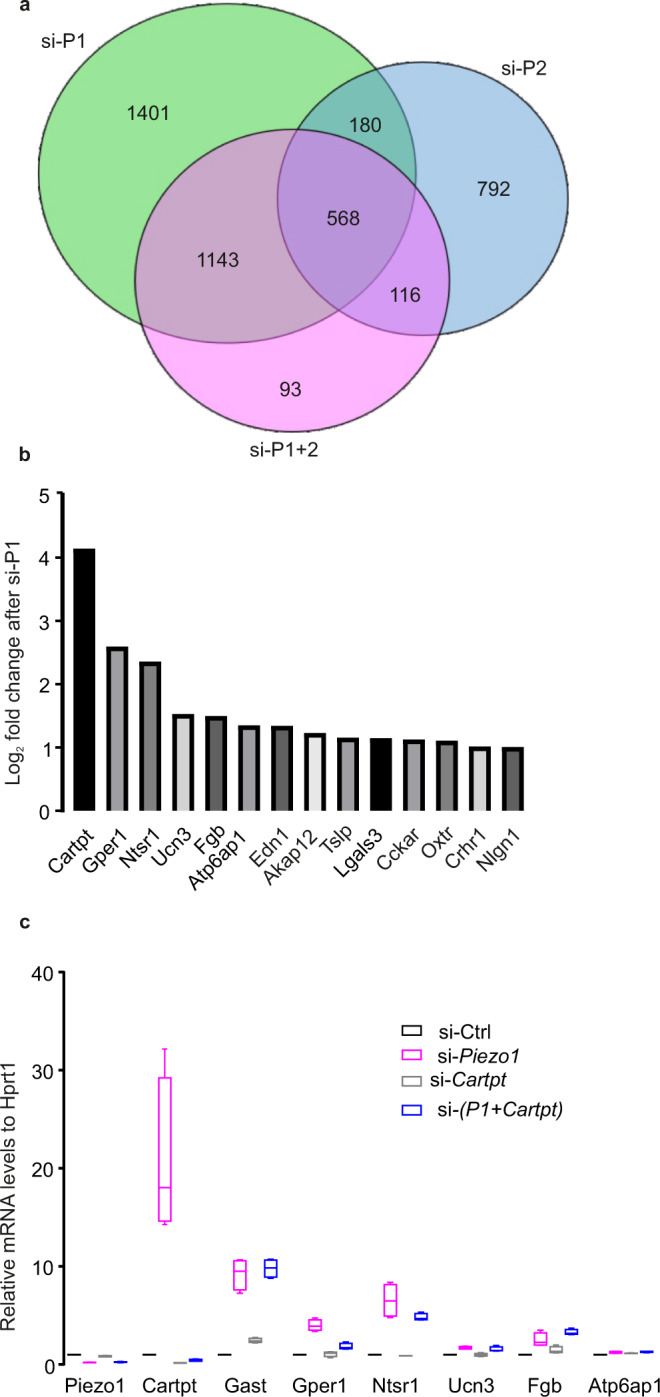
Piezo1-regulated gene networks. **a** Overall differentially expressed genes’ counts after silencing *Piezo1* (si-P1), *Piezo2* (si-P2), *Piezo1* and *Piezo2* (si-P1+2). **b** Log_2_-fold changes for top 14 genes involved in the GO term- “positive regulation of secretion by cell” compared si-*Piezo1* to si-Ctrl. **c** The relative mRNA expression of the identical top 7 DE genes as in **b**, from cells of after silencing of *Piezo1* (si-*Piezo1*, *n* = 4), silencing of *Cartpt* (si-*Cartpt*, *n* = 4), and double silencing of both (si-*P1+Cartpt*, *n* = 4). Data are presented as box Tukey plot, and the definition of box Tukey is as indicated as in Fig. 1.

The translocation of PIEZO1 into the nucleus in response to hyperglycemia suggests that PIEZO1 might be involved in transcriptional control in addition to serving as a mechanosensor in the plasma membrane. To address this, we performed mRNA sequencing in INS-1 832/13 cells to determine which genes regulated by Piezo channels. Overall, we found 3292, 1656, and 1920 genes were significantly differentially expressed after silencing *Piezo1*, *Piezo2*, or both genes, respectively (Fig. 7a). Of the 3292 genes affected by silencing *Piezo1*, 1452 were downregulated and 1394 upregulated. Gene ontology (GO) terms of enrichment was then performed and significantly enriched terms were selected (Fig. S10). Silencing of *Piezo1* was associated with downregulation of 58 and 42 genes involved in the regulation of “intracellular transport” and “nucleocytoplasmic transport”, respectively. These genes represent candidates for further study of the mechanism of PIEZO1 redistribution. Notably, 68 genes involved in “positive regulation of secretion” were upregulated by silencing of *Piezo1*. Among these, the expression of the gene encoding cocaine- and amphetamine-regulated transcript (*Cartpt*) was increased >15-fold (Fig. 7b). We confirmed this effect by qPCR, which indicated a corresponding stimulation of expression (Fig. 7c). In insulin-secreting INS-1 832/13 cells, Cart has been reported to influence the expression and release of insulin^41^. Thus, the impact of PIEZO1 on β-cell function may not be limited to electrical excitability but these changes are likely to operate on a slower timescale than the acute/electrical effects.

## Discussion

**Fig. 8:**
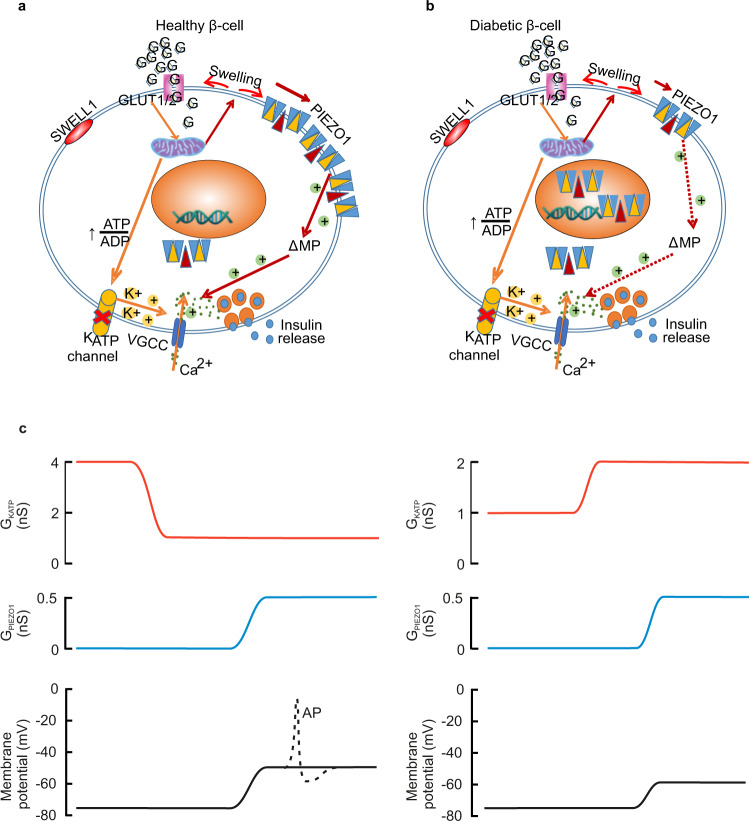
Model of PIEZO1 functions in the regulation of insulin secretion in healthy and diabetic β cells. **a** An increase in plasma glucose and intracellular accumulation of glucose metabolites induce cell swelling and this leads to the activation of plasmalemmal PIEZO1 channels. The opening of these cation-conducting PIEZO1 channels leads to a small membrane depolarization, which in turn leads to activation of voltage-gated Ca^2+^ channels (VGCC) culminating in the elevation of [Ca^2+^]_i_ and stimulation of insulin release. Other channels, including (but not limited to) SWELL1, also provide a depolarizing current but full glucose-induced depolarization requires the cooperation of PIEZO1 and these additional conductances. **b** In β cells from individuals with T2D, PIEZO1 is translocated into the nucleus and the resultant reduction of membrane-associated PIEZO1 curtails the depolarization that would normally result from the glucose metabolism-induced cell swelling, culminating in impaired glucose-stimulated insulin secretion. **c** *Left*: Changes in membrane potential occurring when G_KATP_ is reduced from 4 to 1 nS and G_PIEZO1_ then increases from 0 to 0.5 nS. The associated depolarization approaches the threshold for action potential firing (dotted lines). *Right*: Changes in membrane potential when G_KATP_ is increased to 2 nS and G_PIEZO1_ then increases to 0.5 nS. In this case, depolarization is not sufficient for action potential firing to occur, explaining the suppression of insulin secretion. Membrane potential changes estimated from Eq. 1. AP action potential.

Figure 8 presents a schematic model that delineates the roles of PIEZO1 and SWELL1 in healthy insulin-secreting β cells (Fig. 8a) and how it may become disrupted in type 2 diabetes (Fig. 8b). An increase in plasma glucose and intracellular accumulation of glucose metabolites induces cell swelling and this leads to the activation of plasmalemmal PIEZO1 channels. The opening of these cation-conducting PIEZO1 channels leads to a small membrane depolarization, which in turn leads to activation of voltage-gated Ca^2+^ channels culminating in the elevation of [Ca^2+^]_i_ and stimulation of insulin release.

The crosstalk between PIEZO1 and other membrane conductances in the β cells is illustrated in Fig. 8c. The relative impact of K_ATP_ channels and PIEZO1 channels on the β-cell resting membrane potential (*E*_m_) is given by the simplified constant field equation

1 $${E}_{{{{{{\rm{m}}}}}}}=\frac{{E}_{K}+{\alpha E}_{{{{{{\rm{PIEZO}}}}}}1}}{1+a}$$

where α is the ratio between the whole-cell PIEZO1 and K_ATP_ conductances (*α* = G_PIEZO1_/G_KATP_) and *E*_K_ and *E*_PIEZO1_ are the reversal (equilibrium) potential of the currents flowing through the K_ATP_ and PIEZO1 channels, respectively. The net increase in β-cell resting conductance induced by yoda1 is 0.5 nS and this provides an upper estimate of the magnitude of the PIEZO1-dependent glucose-induced membrane conductance (note that yoda1 retains a stimulatory effect at 10 mM glucose). We acknowledge that the conductance increase measured at negative membrane potential during sucrose infusion at room temperature is limited to 0.2 nS but it is likely that this underestimates the magnitude of the increase at physiological membrane potentials. For example, voltage-gated Ca^2+^ currents are reduced by 50% when the bath temperature is reduced from +37 °C to room temperature^38^. With *E*_PIEZO1_ and *E*_K_ being 0 (Fig. 4q) and −74 mV (measured intact β cells under similar experimental conditions)^42^. At low glucose (1 mM), PIEZO1 activity is low (G_PIEZO1_ ≈ 0) and K_ATP_ channel activity is high (*G*_KATP_ ≈ 4 nS)^43^, the β-cell membrane potential will therefore approximate E_K_, close to the −72 mV as we measured (Fig. 4m). Glucose produced a concentration-dependent decrease in the resting membrane conductance (largely reflecting G_KATP_) and at concentrations ≥10 mM it has fallen to ~1 nS (reflecting cell coupling^43^). This change is accompanied by an increase in G_PIEZO1_ to 0.5 nS. Thus, α increases from 0 to 0.5. From these values, the above relationship predicts a membrane potential at 10 mM glucose of −49 mV, in good agreement with the −52 mV measured experimentally (Figs. 4o and  8c, left). The ability of PIEZO1 to depolarize the β cell is strongly dependent on *G*_KATP_. This explains why even maximal pharmacological activation of PIEZO1 using yoda1 fails to evoke electrical activity and insulin secretion in the presence of the pharmacological K_ATP_ channel activator diazoxide or at subthreshold glucose concentrations. Even a moderate activation of the K_ATP_ channels (e.g. 2 nS) will keep the membrane potential as negative as -60 mV (Fig. 8c, right). It should be noted that this is a conservative estimate of the increase in K_ATP_ channel activity produced by diazoxide; if G_KATP_ were to increase 10-fold (as is observed experimentally^44^), the membrane potential would be −70 mV. In either case, the membrane potential is too negative for regenerative electrical activity to occur even when the PIEZO1 channels are fully active.

The idea that PIEZO1 plays an important—albeit not critical—role in glucose-induced insulin secretion and electrical activity is also supported by the studies in β-cell-specific *Piezo1*-knockout mice. Glucose-induced insulin secretion was reduced by 50% (Fig. 6e), an effect that correlated with weaker glucose-induced (16.7 mM glucose) membrane depolarization (from 27 mV to 11 mV). This suggests that PIEZO1 accounts for 60% of the depolarizing current. Importantly, the PIEZO1-dependent effects normally operate in conjunction with other depolarizing membrane currents. These include SWELL1^9^ (a volume-regulated anion channels; VRAC)^45^ and transient receptor potential (TRP) channels^2^. The existence of such additional depolarizing currents is evident from the observation that tolbutamide remains capable of eliciting electrical activity in *Piezo1*-deficient β cells. When superimposed on residual K_ATP_ channel activity at high glucose^37^, these conductances are not sufficient to produce strong membrane depolarization on their own (explaining the impact of genetic ablating *Piezo1*), and complete inhibition of the K_ATP_ channels that their impact is unveiled. However, individually these depolarizing currents are clearly insufficient to evoke regenerative glucose-induced electrical activity, explaining why the effects of genetically ablating Swell1 and Piezo1 on calcium signaling are not additive (Fig. S3).

Crucially, the finding that the PIEZO1 inhibitor GsMTx4 strongly inhibits glucose-induced insulin secretion in human islets suggests that the channel also plays a central role in human β cells. Our observation that T2D is associated with the translocation of PIEZO1 from the cell surface (plasma membrane) to the nucleus may therefore be pathophysiologically significant as β-cell electrical activity is central to insulin secretion and may account for the diminution of glucose-induced insulin secretion that is a hallmark of T2D^46^. Thus, pharmacological agents that promote the trafficking of PIEZO1 into the plasma membrane (perhaps targeting the residues that mediate the metabolic regulation) may represent a potential therapeutic means of restoring β-cell function in T2D.

## Methods

### Human islet donors

Pancreatic islets/human tissues (fat, liver, and muscle) were obtained from the Nordic Network for Islet Transplantation (Uppsala University, Sweden) via EXODIAB Human Tissue Lab in Lund University with the approval of the ethics committees at Uppsala and Lund. Informed consent was obtained from pancreatic donors or their relatives in accordance with the approval by the local ethics committee at Uppsala and Lund Universities regarding organ donation for medical research. The RNA-seq data were analyzed from 181 donors (114 healthy, 40 pre-diabetes, 27 diabetes). Of these, islets from 12 healthy (8 males + 4 females) and 1 diabetic (male) donors were used for experiments. More information about the donors was listed in Supplemental Table 1.

### Animal models

Rats and mice used were approved by the Malmö/Lund and Gothenburg Animal Care and Use Committee and abided by the guide for the care and use of the laboratory animals published by the Directive 2010/63/EU of the European Parliament. Animals were allocated to experimental groups by genotype/diabetic phenotype. All the animal experiments were conducted according to ARRIVE guidelines^47^. All the animals were kept in a pathogen‐free facility on a 12–12 h light–dark cycle at temperature of 22 °C with humidity of 55%. The chow food the animals have taken for the whole life was Cat# A30 from SAFE complete care competence.

### β-cell-specific *Piezo1*-knockout mice

β-cell-specific *Piezo1*-knockout mice (RIP-Cre^+^.Piezo1^f/f^) were generated by mating mice expressing the Cre recombinase gene under the control of rat insulin 2 gene promoter (RIP-Cre^+^)^48^ with Piezo1^tm2.1Apat/J^ (also known as P1^f^) (Stock #029213, The Jackson Laboratory) mice^12^ to obtain RIP-Cre^+^. Piezo1^f/+^ mice, which were then crossed with P1^f^ to get RIP-Cre^+^.P1^f/f^ knockout (KO) mice. Mice were genotyped using the following primers: Cre F: GCA TTA CCG GTC GAT GCA ACG AGT GAT GAG, Cre R: GAG TGA ACG AAC CTG GTC GAA ATC AGT GCG using dreamtaq hot start DNA polymerase (#EP1702, Thermofisher) with the following cycling conditions: 95 °C, 1 min; 95 °C, 30 s, 68 °C, 3 min (35 cycles); 68 °C 3 min. Reactions were separated on 2% agarose gels yielding a ≈ 400 bp band. The same sample was used for P1 F: GCC TAG ATT CAC CTG GCT TC, P1 R: GCT CTT AAC CAT TGA GCC ATC T using the same polymerase as Cre with the following cycling conditions: 95 °C, 4 min; 95 °C, 30 s, 65 °C, 30 s, 72 °C, 30 s (35 cycles); 72 °C, 5 min. The following band sizes were observed for P1^f/f^: ≈380bP, P1^f/+^: ≈380 bp and 188 bp. RIP-Cre^+^ mice were used as controls. The mice with Cre positive and P1^f/f^ genotype were selected as homozygous *Piezo1-*knockout mice and the mice with Cre positive and P1^f/+^ genotype were regarded as heterozygote. Confirmation of *Piezo1* deletion by qPCR was performed using primers targeting exons 21-22 (Mm01241547_g1, Cat # 4448892, Thermofisher) or exons 22–23 (Mm01241548_m1, Cat # 4448892, Thermofisher) of *Piezo1*.

### Wistar rats

Islets isolated from male Wistar rats (≥11 weeks old) were used. A minimum of 3 rats were used in each group.

### db/db and C57BL/6J mice

Islets were isolated from male *db/db* mice and age-matched control male C57BL/6 J mice (≥12 weeks of age). At least 3 mice were used in each group.

### Cell culture

INS-1 832/13 cells were derived from parental INS-1 cells and express the human proinsulin gene^49^. INS-1 832/13 cells were cultured in RPMI 1640 (HyClone) medium containing 11.1 mΜ d-glucose (Sigma) supplemented with 10% fetal bovine serum (Sigma), 10 mM HEPES (HyClone), 100 U/ml penicillin (HyClone), 100 μg/ml streptomycin (HyClone), 2 mM l-glutamine (HyClone), 1 mM sodium pyruvate (HyClone) and 0.05 mΜ 2-mercaptoethanol (Sigma), at 37 °C in a humidified atmosphere containing 95% air and 5% CO_2_.

### Islet isolation

Rats and mice were killed by high CO_2_ and cervical dislocation, respectively. Collagenase solution (Boehringer Mannheim Collagenase P, 1 mg/ml, pH 7.4) was injected into the main pancreatic duct. The pancreas was resected and incubated for 17 min at 37 °C. The islets were handpicked under a stereomicroscope. Whole islets were cultured in petri dishes (Sarstedt) containing RPMI 1640 (HyClone) as above but substituted with 5 mM d-glucose and 10% fetal bovine serum (for rat islets)/10 mM d-glucose and 5% fetal bovine serum (for mouse islets) and lacking 2-mercaptoethanol.

### Dissociation of islet cells

Fresh whole islets were picked up manually and incubated in Ca^2+^ free solution (in mM: 138 NaCl, 5.6 KCl, 1.2 MgCl_2_, 5 HEPES, 3 glucose, 1 EGTA, 1 mg/ml BSA) for 12 min at 37 °C. Free β cells were released by gentle agitation. The resulting cell suspension was centrifuged. After discarding the supernatant, the cell pellet was resuspended in the culture medium mentioned above. Finally, the resuspended cells were seeded on glass-bottomed dishes for further experiments.

### RNA interference and real-time quantitative PCR

INS-1 832/13 cells were seeded by 150,000 per well in 24-well plates in penicillin/streptomycin-free culture medium prior to transfection the next day. Rat ON-TARGET plus smart-pool *Piezo1* (#L-093854-02-0005, Dharmacon) or *Piezo2* (#L-092136-02-0005, Dharmacon) RNA interference oligonucleotides (final concentration: 25 nM) were used to silence *Piezo1* or *Piezo2* and ON-TARGET plus negative control (#D-001810-10-20, Dharmacon) was served as a control. The siRNA was transfected using Lipofectamin^®^ RNAimax (#13778150, Thermofisher Scientific). Double transfection with the same protocol was performed after 48 h and maintained for another 48 h for later measurements.

The cells, islets were collected and extracted for mRNA by using RNeasy^®^ plus Mini kit (#74136, Qiagen), mRNA from mouse tissues was extracted using miRNeasy^®^ Mini kit (#1038703, Qiagen) following the manufacturers’ instruction. Reverse transcription was performed with 500 ng mRNA. The resulting cDNA was used for the expressions of *Piezo1* (Primer: #Rn01432593_m1 for cells and rat islets, #Mm01241549_m1 for mouse), *Piezo2* (Primer: #Rn01491821_m1 for cells and rat islets, #Mm01265861_m1 for mouse) and housekeeping gene *Hprt1* (Primer: #Rn01527840_m1 for cells and rat islets, Mm03024075_m1 for mouse, Thermofisher Scientific) by real-time quantitative PCR (RotorGene 2000, Corbett Research) in 10-μl reactions containing 2× universal PCR Master Mix (#4304437, Thermofisher Scientific) and 0.2 mM primers. The results were calculated after normalization to the expression levels of *Hprt1* in each sample.

### Western blotting

INS-1 832/13 cells or human islets were lysed with RIPA buffer (150 mM NaCl, 1% Triton X-100, 0.1% SDS, 0.5% Sodium deoxycholate, 2 mM EDTA, 50 mM NaF) with 2% protease inhibitor cocktails (#11873580001, Roche). The lysates were centrifuged at 10,000 g for 10 min at 4 °C and the supernatant was collected for western blot after testing the protein concentration by Pierce BCA assay kit (#23225, Thermofisher Scientific). The protein lysate (35 to 50 µg of total protein) was mixed with loading buffer (pH 6.8; 2% SDS, 10% glycerol, 0.1% bromophenol blue, 100 mM DTT, 50 mM Tris-HCl) supplemented with 1 mM DTT and heated at 70 °C for 10 min, followed by SDS-PAGE (using 4–15% Mini-PROTEAN^®^ Criterion TGX Stain-Free gels; #456-8084, Bio-Rad) and transferred to a PVDF membrane (Bio-Rad).

The gel was transferred to the PVDF membrane in the transferring buffer (57.66 mM Tris, 191.8 mM glycine, 0.1% SDS, 20% ethanol) at a constant current of 380 mA for 70 min at 4 °C. The total protein on the membrane after transferring was analyzed before blocking in 5% skim milk in TBST buffer (20 mM Tris, 150 mM NaCl, 0.1% Tween) for at least 30 min. The membrane was cut according to the molecular weight of the protein and incubated with primary antibodies of Piezo1 (1:1000, #15939-1-AP, Proteintech) or β-actin (1:1000, #A2228, Sigma) overnight at 4 °C. On the following day, the PVDF membrane was washed with TBST buffer 5 times (5 min each), incubated with anti-rabbit or mouse horseradish peroxidase (HRP)-conjugated secondary antibodies (1:1000, #7074, Cell Signaling Technology and #P0447, Dako) and kept at room temperature for 1 h. The membrane was washed with TBST buffer as before. Enhanced chemiluminescent (ECL) substrates were employed for the detection of HRP enzyme activity. For detection of the low levels of Piezo1, SuperSignal™ West Femto Maximum Sensitivity Substrate was used. Data were analyzed and quantified with the Image-Lab 5.2.1 program, e.g., normalization factor in Fig. 1c was calculated by the Image-Lab 5.2.1 as (total reference lane protein/total target lane protein), and this was used to adjust the target band intensity.

### Purification of plasmids and overexpression

The plasmids of Piezo1 aa2189-2547-GFP, aa2189-2458-GFP, aa2458–2547-GFP were assembled into pcDNADest47_A095, purified and verified by Thermofisher Scientific. The resulting plasmids were then amplified and purified by transforming to One-Shot Stbl3 chemically competent cells (#C7373-03, Thermofisher Scientific), heat-shocked and grew on LB agar plates, a single colony was picked for culturing and the DNA was purified using Qiagen Maxiprep purification kit (#12162, Qiagen) following the manufacturers’ instruction. The DNA concentration was measured by Nanodrop. The plasmids including the GFP control were transfected into INS-1 832/13 cells using Lipofectamine™ 3000 Transfection Reagent (#L3000008, Thermofisher Scientific) for 72 h and the fluorescence of GFP was captured by confocal microscopy (LSM 510, Carl Zeiss, Germany).

### Immunostaining

Single cells were cultured in µ-Slide (chambered coverslip) with 8 wells (ibidi, Germany) one day prior to immunostaining. Cells were first washed twice with PBS (Hyclone) and fixed with 3% PFA-K-PIPES and 3% PFA-Na_2_BO_4_ for 5 and 10 min, respectively, followed by permeabilization with 0.1% Triton X-100 for 30 min at room temperature. The cells were incubated with the blocking solution containing 5% normal donkey serum (Jackson immunoresearch) in PBS for 30 min. Primary antibodies against Piezo1 (1:200, #15939-1-AP, Proteintech), insulin (1:400, #16049, Progen) or glucagon (1:200, #ab10988, Abcam) were diluted in blocking solution and incubated overnight at 4 °C. Immunoreactivity was detected by fluorescently labeled secondary antibodies (1:400, anti-rabbit Alexa Flour 488 secondary antibody (711-545-152, Jackson ImmunoResearch), anti-mouse cy3 (715-165-151, ImmunoResearch), anti-guinea pig Alexa647 (706-165-148, Jackson ImmunoResearch)) and visualized by confocal microscopy (LSM 510, Carl Zeiss, Germany). It was ascertained that the PIEZO antibody did not label β cell in *Piezo1-*knockout islets pointing out the specificity of PIEZO1 antibody. The membrane was defined as the first 0.5 µm from the surface edge. In Fig. 1, the ratio was calculated as *F*_nucleus_/*F*_cytosol_. In Fig. 2, the ratio of mean intensity of the protein in the nucleus, cytosol, and surface membrane by that in the whole cell were calculated according to the formula: Ratio = *F*_segment_/*F*_total_, where *F*_segment_ represents the fluorescence intensities of nucleus, cytosol, and surface membrane, and F_total_ represents the fluorescence intensity of the entire cell.

Mouse islets were cultured with 5, 10, or 20 mM glucose for 72 h, followed by immunostaining using a standard protocol. In brief, islets were washed with PBS twice and fixed with 3% PFA in PBS with 0.1% Triton X-100 for 30 min at room temperature, the islets were permeabilized with 0.1% Triton X-100 overnight at 4 °C. The next day, the islets were incubated with the blocking solution containing 5% normal donkey serum (Jackson immunoresearch) in PBS with 0.1% Triton X-100 for 30 min, followed by incubation with primary and secondary antibodies as above.

### Ratiometric Ca^2+^ imaging

INS-1 832/13 cells after silencing of *Piezo1* or *Piezo2* for 72 h, dispersed islets cells from rats, human or Cre^+^/Cre^+^.P1^f/f^ mice were seeded onto 14-mm round poly-L-lysine-coated glass-bottom culture dishes, cultured in normal culture medium for another 24 h, then proceeded for cytosolic Ca^2+^ imaging. Prior to the assay, cells were washed with a Krebs buffer (pH 7.4 with NaOH, in mM: 120 NaCl, 4.7 KCl, 2.5 CaCl_2_, 1.2 KH_2_PO_4_, 1.2 MgSO_4_, 25 NaHCO_3_, 10 HEPES and 2.8 glucose) once and incubated with 1 µM Fura-2 AM (#F1221, Thermofisher Scientific) in 1 ml Krebs buffer for 30 min at 37 °C with 5% CO_2_ in the incubator. The cells were washed twice with Fura-2 free Krebs buffer. Next, cells in the dish were perfused at 1 ml/min at 32 °C with Krebs buffer or hypotonic Krebs buffer with 60 mM NaCl supplemented with different substances as indicated. Yoda1 (SML1558, Sigma) was served as an agonist for activating the channel of Piezo1.

The fluorescence from Fura-2 AM was monitored at 340/ 380 nm excitation and 510 nm emission using a Polychrome V high-speed switching monochromator on a Nikon microscope equipped with an Andor ER-BOB-100 trigger box, an Andor camera Ixon3, and IQ2 software. The area under the curve (AUC) was calculated by GraphPad Prism 7 software, according to

2 $${{{{{\rm{AUC}}}}}}\,=\sum [0.5({F}_{{i}}/{F}_{{0}}+{F}_{i-1}/{F}_{{0}})\times ({t}_{i}\,{-}\,{t}_{i-1})]$$

where F_i_ and F_i-1_ represent the ratios calculated with fluorescence intensity excited by 340 nm to that was by 380 nm wavelength from two neighboring images (*i* to *i*-1) followed in the entire time course. *F*_0_ represents the basal condition which calculated the mean ratio from images that acquired in the first 1 min before stimulation. *t*_i_ – *t*_i−1_ is the time interval of the neighboring images.

Because of dye bleaching, linear slope correction was applied in some experiments.

### Electrophysiology

#### Perforated-patch clamp

Electrical activity was measured from β cells within intact mouse islets using the perforated-patch technique. All measurements were performed between 32–34 °C. During the experiments, the islets were immobilized using a wide-bore glass suction pipette^35^ and superfused with a modified Krebs–Ringer solution containing (mM) 140 NaCl, 3.6 KCl, 1.3 CaCl_2_, 0.5 MgSO_4_, 10 HEPES, 0.5 NaH_2_PO4, and 5 NaHCO_3_ at pH 7.4 with NaOH and glucose as indicated. The solution within the pipette contained 76 K_2_SO_4_, 10 KCl, 10 NaCl, 1 MgCl_2_, and 5 HEPES (in mM, pH 7.35 using KOH). Membrane perforation was effected by inclusion of the pore-forming antibiotic amphotericin B (240 μg/ml) in the intracellular buffer. Islet cell types were established by functional fingerprinting^36^. Measurements were performed using an EPC-10 patch-clamp amplifier (HEKA Electronics, Ludwigshafen/Rhein, Germany) and PatchMaster software (version 2 × 91).

#### Whole-cell patch clamp

Swelling-induced currents were recorded from β cells in intact mouse islets using the standard whole-cell technique. The extracellular solution consisted of (mM): 118 NaCl, 20 TEA-Cl, 5.6 KCl, 2.6 CaCl_2_, 10 HEPES (pH adjusted to 7.4 with NaOH), 1.2 MgCl_2_, 1 glucose and 1 CoCl_2_. The intracellular solution contained (mM): 140 CsCl, 2 EGTA, 3 MgATP, 1 MgCl_2_, 10 HEPES (pH adjusted to 7.15 with CsOH). To induce cell swelling, 100 mM sucrose was added to the intracellular solution. In some experiments, GsMTx4 (2.5 μM) was added to the extracellular solution. All experiments were performed at room temperature without perfusion. Measurements were performed using HEKA EPC-10 patch-clamp amplifiers with the PatchMaster software (version 2 × 91).

### Cell size measurement

INS-1 832/13 cells were stained with a fluorescent dye carboxyfluorescein succinimidyl ester (CFSE) (#C34570, Thermofisher Scientific). Due to its covalent coupling reaction to intracellular molecules, fluorescent CFSE is retained within cells once incorporated. Briefly, the cells were incubated with 5 µM CFSE in Krebs buffer containing (mM) 120 NaCl, 4.7 KCl, 2.5 CaCl_2_, 1.2 KH_2_PO_4_, 1.2 MgSO_4_, 2.8 glucose, 25 NaHCO_3_, 10 HEPES (pH 7.4 with NaOH) for 30 min at 37 °C with 5% CO_2_. The cells were washed twice with Krebs buffer (as above) with 1 mg/ml BSA. Next, the cells were incubated for a further 10 min to allow CFSE to undergo full hydrolysis. Subsequently, the cells were perfused at a rate of 1 ml/min at 32 °C with Krebs buffer containing 2.8 or 16.7 mM glucose, respectively. The fluorescence from CFSE was monitored every min at excitation wavelength 492 nm and the emitted light signals were read at 517 nm long-pass filter on confocal microscopy (LSM 510, Carl Zeiss, Germany). The cell area was measured by ImageJ software (version 1.52a).

### Fluorimetric measurements of membrane potential

The membrane potential was monitored in INS-1 832/13 cells with the indicator PMPI (R-8042; Molecular Devices, Sunnydale, CA), it is loaded into the cells for 30 min as recommended by the manufacturer. After loading with the dye, the cells were superfused at 1 ml/min (32 °C) with Krebs buffer containing PMPI supplemented as indicated. The fluorescence from PMPI was monitored at excitation wavelength 514 nm and the emitted light signals were read at 530 nm long-pass filter on confocal microscopy (LSM 510, Carl Zeiss, Germany).

### Insulin secretion assays

#### Cells

INS-1 832/13 cells (150,000 cells per well) were cultured in 24-well plates (Sarstedt, USA) and transfected with siRNA target to *Piezo1* for 96 h. The cells were washed with 1 ml secretion assay buffer (SAB) (114 mM NaCl, 4.7 mM KCl, 1.2 mM KH_2_PO_4_, 1.16 mM MgSO_4_, 25.5 mM NaHCO_3_, 2.6 mM CaCl_2_, 20 mM HEPES (pH 7.3), 0.2% BSA) with 2.8 mM glucose twice, and pre-incubated in 2.8 mM glucose SAB for 2 h at 37 °C with 5% CO_2_ in the incubator. Insulin secretion was measured in static incubations of cells (500 μl SAB containing 2.8 or 16.7 mM glucose for 1 h). In the experiments involving hypotonicity, the concentration of NaCl was reduced to 54 mM NaCl. The supernatant was collected pending later measurements of insulin release. Insulin content was determined after lysing the cells with 200 μl RIPA buffer (see above) followed by shaking on ice for at least 20 min. Insulin concentrations were measured with a high-range rat insulin ELISA (#10-1145-01, Mercodia AB) and normalized to protein content. Protein content was determined using the Pierce™ BCA protein assay kit (#23225, Thermo Fisher Scientific).

#### Islets

Rat islets were handpicked and freshly used for insulin secretion. Islets were pre-incubated in 3.5-cm petri dishes at 37 °C and 5% CO_2_ for 30 min in Krebs buffer containing 2.8 mM glucose and 1 mg/ml BSA. Next, groups of 6 islets in each well were incubated in triplicates for each condition in 500 μl Krebs buffer containing 2.8 or 16.7 mM glucose, incubated at 37 °C with 5% CO_2_ for 1 h. GsMTx4 (#ab141871, Abcam) was used as an inhibitor of PIEZO1. Samples (400 μl) were taken for secretion in measurements. The islets were lysed by the addition of 100 μl RIPA buffer and samples were taken for determination of insulin contents. Secreted insulin in rat islets was measured using the same ELISA kit as above and data was normalized to insulin content. Human islets were obtained from the Scandiatransplant unit in Uppsala. Samples were taken as described above for rat islets and insulin concentrations measured using the human insulin ELISA kit (#10-1113-10, Mercodia AB).

#### In situ pancreas perfusion

Dynamic measurements of insulin secretion were performed using in situ pancreatic perfusion. Non-fasted C57BL/6 J mice were injected intraperitoneally with heparin (2000 units/kg, MT#49001, AstraZeneca) and then killed by rising CO_2_. After opening the abdominal cavity and ligating the renal, hepatic, splenic, superior mesenteric and inferior mesenteric arteries, the aorta was tied off above the level of the pancreatic artery and a silicone catheter connected to a cannula Butterfly needle (27G) placed in the celiac aorta. The pancreas was perfused at a rate of 1 ml/min using a KDS Legato 100 series syringe pump (KD Scientific). The perfusion medium consisted of (in mM) 120 NaCl, 4.7 KCl, 2.5 CaCl_2_, 1.2 KH_2_PO_4_, 1.2 MgSO_4_, 25 NaHCO_3_, 10 HEPES, 2.8 or 16.7 glucose. The pH was balanced with O_2_/CO_2_ (95:5) and the temperature maintained at +37 °C using a heating pad (#HK35, Beurer GmbH). The perfusion medium was supplemented with 1 mg/ml BSA and filtered using the Filtropur S 0.2 unit (#83.1826.001, Sarstedt). The pancreas was first perfused with 2.8 mM glucose Krebs buffer for 20 min and samples were then collected every 1 min under the indicated experimental conditions via a silicone catheter inserted into the portal vein. Insulin in the effluent medium was determined by a radioimmunoassay (RIA) (# RI-13K, EMD Millipore Corporation).

#### IPGTT

Intraperitoneal glucose tolerance test (IPGTT) was performed on 5–8 week-old male Cre^+^, Cre^+^.P1^f/+^, Cre^+^.P1^f/f^ mice without fasting period. Blood glucose levels were measured with a handheld glucometer (OneTouch; Lifescan) prior to an intraperitoneal injection of 2 g glucose/kg body weight. Blood glucose was measured at 0, 5, 15, 30, 60, and 120 min after glucose injection.

#### mRNA-sequencing data analysis

mRNA was extracted from Piezo1, Piezo2, or Piezo1+Piezo2-silenced INS-1 832/13 cells as described previously^50^ for library preparation and mRNA sequencing. Sequencing reads were mapped to the Ensembl rat genome (Rnor_6.0) and quantified by Salmon (v.0.14.0). Differential gene expression analysis was performed using DESeq2 (v. 1.25.10). Genes with non-zero counts in at least 50% of the samples were included in the differential expression analysis. Genes with an adjusted *p* value < 0.05 were considered to be differentially expressed. Piezo1- (si-P1), Piezo2- (si-P2) and Piezo1+Piezo2- (si-P1+2) silenced samples were compared against the non-targeting siRNA treated samples (si-Ctrl, scramble siRNA) to obtain the differentially expressed gene list.

Genes were grouped according to their expression profiles using hierarchical clustering with the R package DEGreport (v.1.21.1). GO terms enrichment was performed using the R package ClusterProfiler (v. 3.13.0). Statistically significant enrichment was determined using an adjusted *p* value (*q* value) < 0.05 after Benjamini-Hochberg correction for multiple hypotheses. Selected GO terms were plotted using a custom R script.

### Statistical analysis

All statistical analyses were performed using GraphPad Prism 7. The data are presented as means ± SEM for indicated number (n) individual repeats or number of cells when indicated. Statistical tests applied are indicated in the figure legends. Evaluation of statistical significance was done using either Student’s t-test or two-way analysis of variance (ANOVA) with Friedman test for multiple comparisons. In all figures, *p < 0.05, **p < 0.01, ***p < 0.001, ****p < 0.0001.

### Reporting summary

Further information on research design is available in the Nature Research Reporting Summary linked to this article.

## Supplementary information

Supplementary Information

Reporting Summary

Peer Review File

## Data Availability

The authors declare that all data supporting the findings of this study are available within the article and its Supplementary Information Files or from the corresponding author on reasonable request. A reporting summary for this Article file is available as a Supplementary Information file. The RNA sequence raw data has been deposited in NCBI’s Gene Expression Omnibus (GEO) with GEO Series accession number GSE197449. Source data are provided with this paper.

## Source data

Source data
