# Supplementary material for: A critical role of the mechanosensor PIEZO1 in glucose-induced insulin secretion in pancreatic β-cells
Source: Nat Commun. 2022 Jul 22;13:4237. doi: 10.1038/s41467-022-31103-y (PMC9307633; doi:10.1038/s41467-022-31103-y)
Supplement: Supplementary file 1 — Supplementary Information [file 41467_2022_31103_MOESM1_ESM.pdf]

Supplementary Information

A critical role of the mechanosensor Piezo1 in glucose-induced insulin secretion in pancreatic β-cells

Yingying Ye, Mohammad Barghouth, Haiqiang Dou, Cheng Luan, Yongzhi Wang, Alexandros Karagiannopoulos, Xiaoping Jiang, Ulrika Krus, Malin Fex, Quan Zhang, Lena Eliasson, Patrik Rorsman, Enming Zhang, Erik Renström

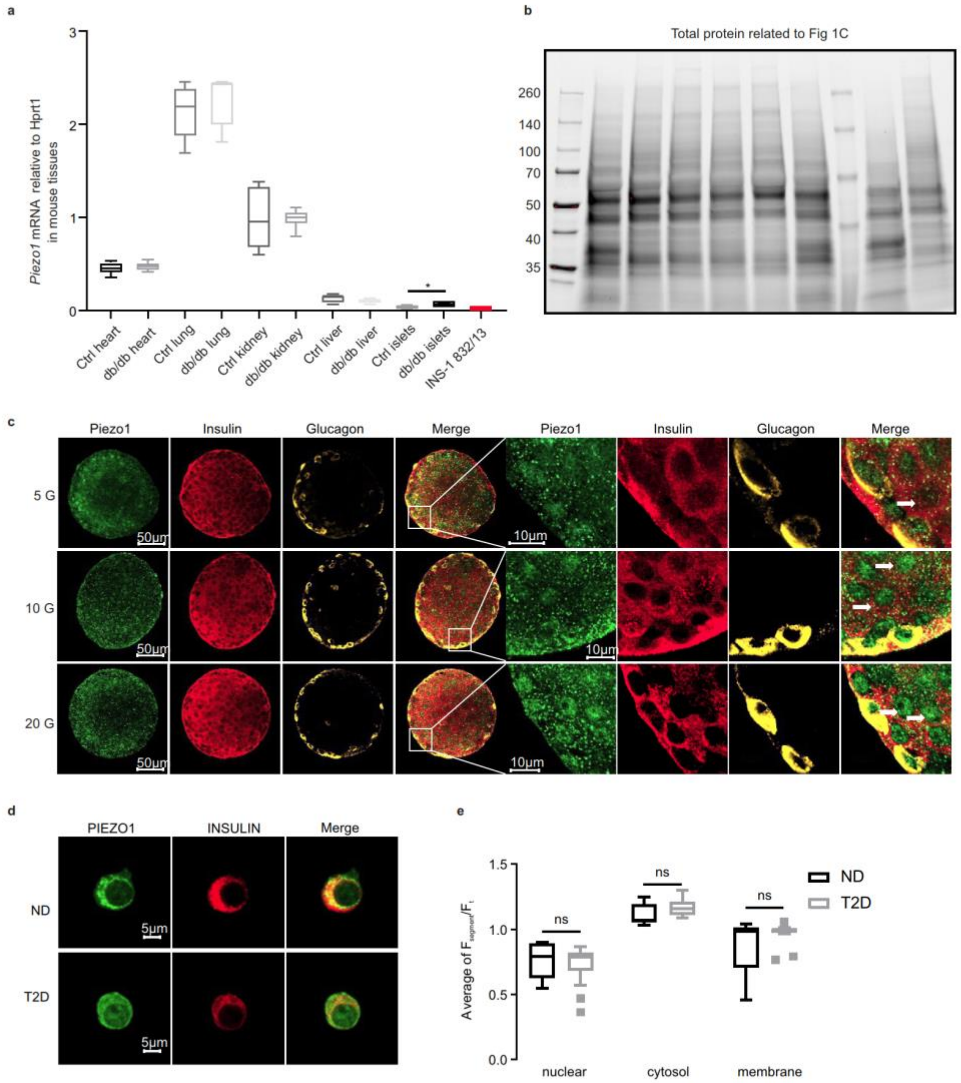

Figure S1 Piezo1 expression in different tissues.

a. Messenger RNA expression of *Piezo1* in C57BL/6J (Ctrl) and *db*/*db* mouse tissue (n=3), p=0.026 between the expression in islets from Ctrl and *db*/*db* mice.

b. Total protein for normalization in Figure 1c.

c. Whole mouse islet immunostaining for PIEZO1 (green), insulin (red) and glucagon (yellow) after culture in 5, 10 or 20 mM glucose, as indicated. Images are representative of 5 experiments for each condition.

d. Immunostaining of PIEZO1 (green) and insulin (red) in human islets from healthy donors (ND) and donors with type 2 diabetes (T2D).

e. The ratio of fluorescence for PIEZO1 in the nucleus, cytosol and membrane normalized to the total PIEZO1 fluorescence calculated for **d** (n=12 and 15 cells for ND and T2D,

respectively).

Data are presented as mean values ± S.E.M. Statistical significance were evaluated using two-tailed unpaired Student's *t*-test in **e**, ns: not significant.

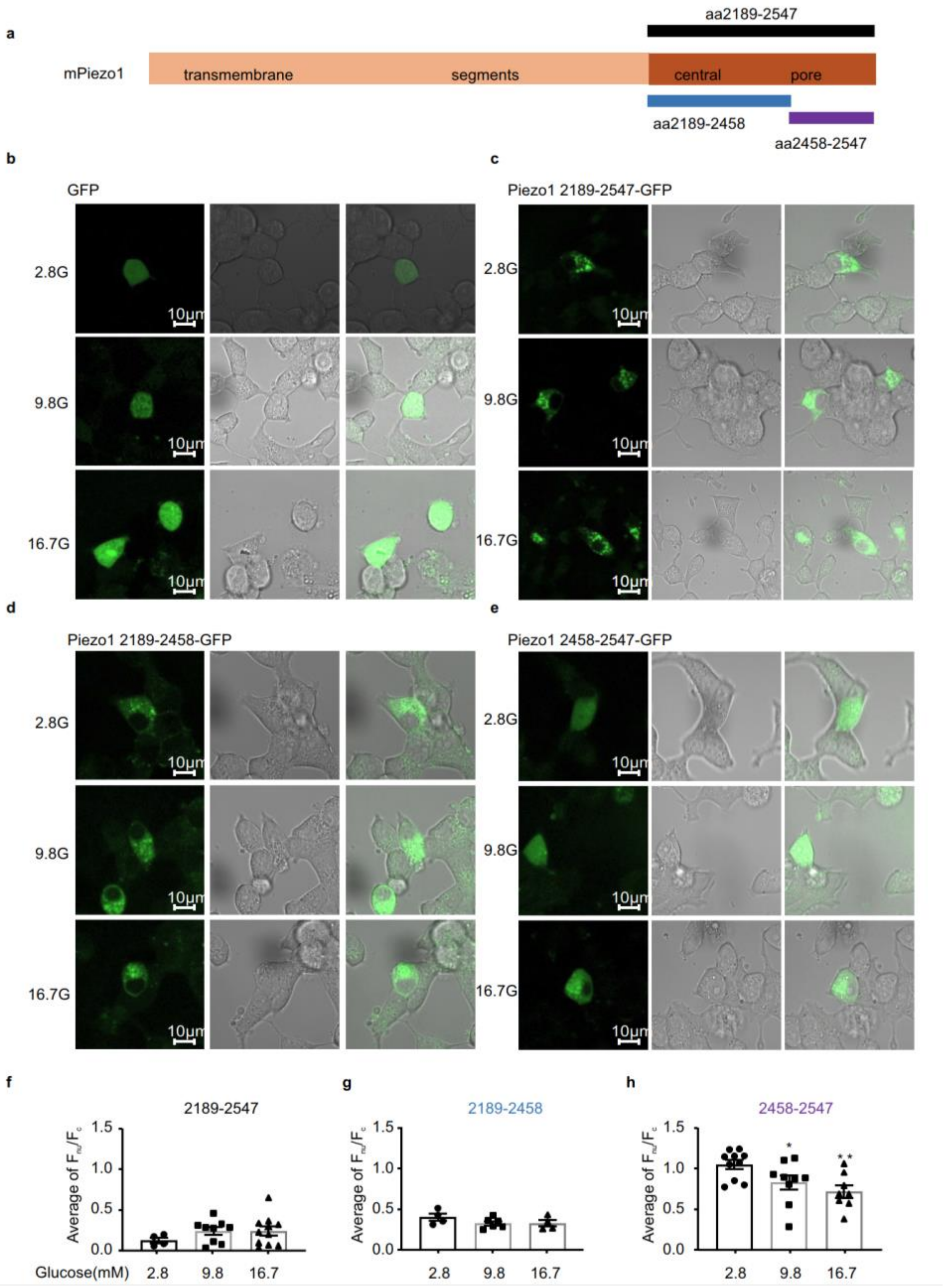
a
aa2189-2547
mPiezo1
transmembrane
segments
central
pore
aa2189-2458
aa2458-2547
b
GFP
2.8G
9.8G
16.7G
c
Piezo1 2189-2547-GFP
d
Piezo1 2189-2458-GFP
e
Piezo1 2458-2547-GFP
f
2189-2547
g
2189-2458
h
2458-2547
Average of F_{nu}/F_c
Glucose(mM)
2.8
9.8
16.7

Figure S2 Translocation of Piezo1 fragments in INS-1 832/13 cells.
a. Cartoon of mouse Piezo1 and fragments used.
b-**e**. GFP fluorescence for control-GFP (empty vector) (**b**), Piezo1 aa2189-2547-GFP (**c**), Piezo1 aa2189-2458-GFP (**d**), Piezo1 aa2458-2547-GFP (**e**).
f. Ratio of intensity for GFP tagged Piezo1 aa2458-2547 in nucleus over that in the cytosol in (**c**) (n=4, 9 and 11 cells in 2.8 mM, 9.8 mM or 16.7 mM glucose (G);
g: As in (**f**), but for (**d**; Piezo1 aa2189-2458-GFP) n=4, 6 and 4 cells, respectively;
h: As in (**f**), but for (**e**; Piezo1 aa2458-2547-GFP) n=10, 9 and 8 cells, respectively, p values for the comparison of the ratios between 2.8 and 9.8, 16.7 mM G were 0.0456, 0.0025, respectively.
Data are presented as mean values ± S.E.M. Statistical significances were evaluated using two-tailed unpaired Student's *t*-test in **f**, **g**, **h**.

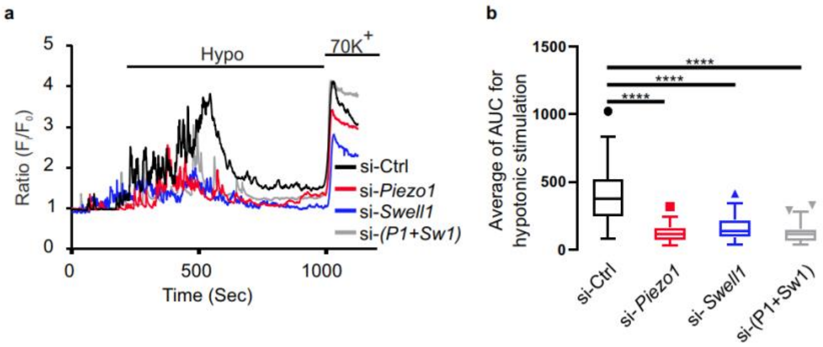

Figure S3 Piezo1 and Swell1 contribute in parallel to Ca^{2+} signaling in response to hypotonic stimulation

a. Traces represent the ratio of Fura-2 AM fluorescence (F_i) normalized to the basal (F_0) upon perfusion of 2.8 mM glucose (0-250 s), hypotonic solutions (250-750 s), followed by stimulation with 70 mM K^+ in negative control (si-Ctrl; *black*), *Piezo1*-silenced (si-*Piezo1; red*), *Swell1*-silenced (si-*Swell1; dark blue*), *Piezo1* and *Swell1*-silenced (si-*P1+Sw1; grey*) INS-1 832/13 cells.

b. The average of area under the curve (AUC) under hypotonic stimulation for **a** (n = 73, 51, 48 and 49 cells for si-Ctrl, si-*Piezo1*, si-*Swell1*, si-*P1+Sw1*, respectively). Color code as in **a**. p values for the comparison of AUC in all conditions to that in si-Ctrl were <0.0001.

Data are presented as mean values ± S.E.M. Statistical significance were evaluated using two-sided one-way ANOVA multiple comparisons for figure **b**, ****$p<0.0001$.

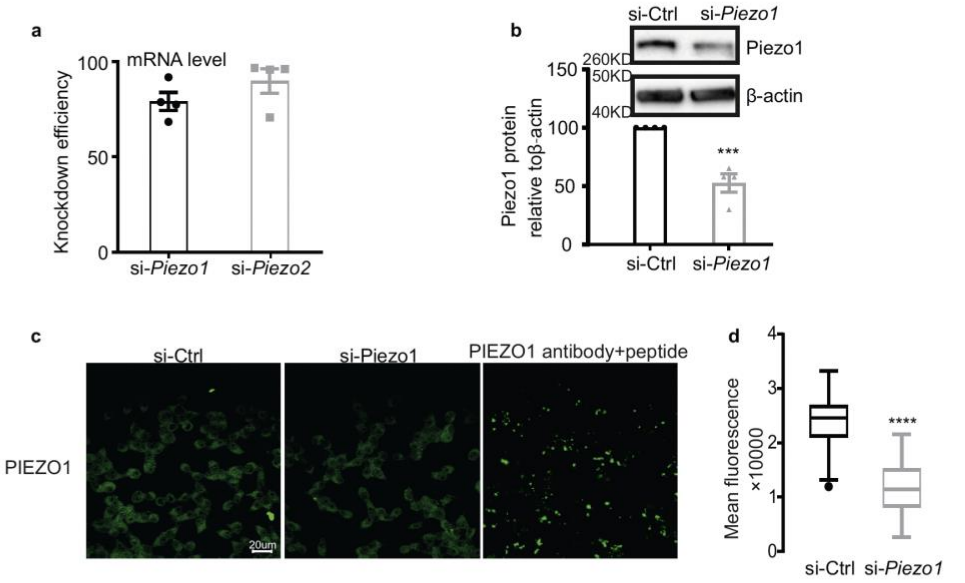

Figure S4 Assessment of Piezo1 or Piezo2 knockdown efficiency in INS-1 832/13 cells.

a. Knockdown efficiency, assessed as mRNA reduction, in *Piezo1* or *Piezo2*-silenced INS-1 832/13 cells (n=4).

b. Knockdown efficiency, assessed as reduction of PIEZO1 protein as compared to negative siRNA (si-Ctrl) treated INS-1 832/13 cells (n=4), p=0.0009.

c. Immunostaining of PIEZO1 in si-Ctrl, *Piezo1*-silenced INS-1 832/13 cells. In the right panel, Fam38a/PIEZO1 fusion protein was used to block PIEZO1 antibody prior to incubation with si-Ctrl treated INS-1 832/13 cells.

d. Mean fluorescence in **c** (n=30 cells in each group), p<0.0001.

Data are presented as mean values ± S.E.M. Statistical significances were evaluated using two-tailed unpaired Student's *t*-test, ***p<0.001, ****p<0.0001.

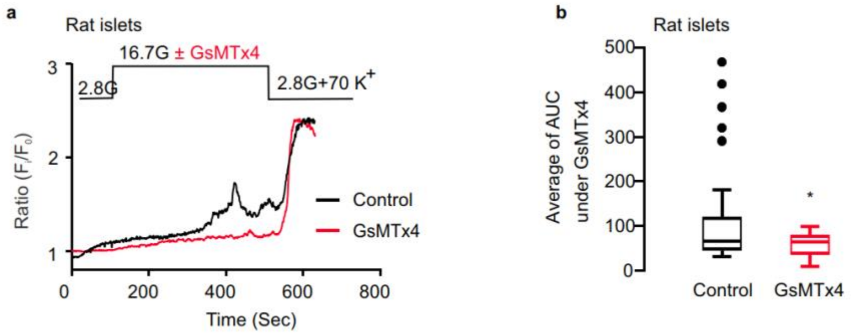

Figure S5. Effects of GsMTx4 on Ca^{2+} in rat islets

a. Representative traces upon perfusion of 2.8 mM glucose (~100 s), 16.7 mM glucose buffer with or without GsMTx4 (~400 s) and followed by 70 mM K^+ stimulation in dispersed rat islet cells.

b. The average of area under the curve (AUC) under GsMTx4 treatment in **a** (n=7 cells for control, n=8 cells for GsMTx4), p=0.0238.

Data are presented as mean values ± S.E.M. Statistical significance were evaluated using two-tailed unpaired Student's *t*-test, *$p < 0.05$.

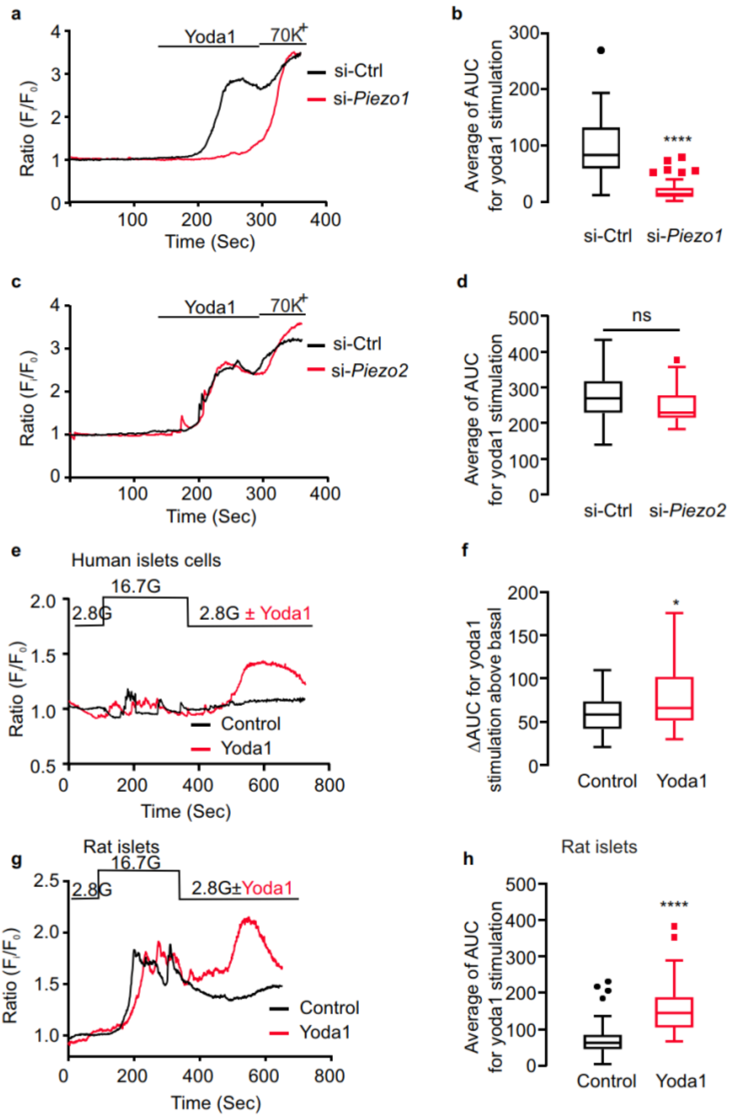

Figure S6 Effects of yoda1 and GsMTx4 on Ca^{2+} signaling in rat islet and INS-1 832/13 cells.

a. Traces represent the ratio of Fura-2 AM fluorescence (F_i) normalized to the basal level (F_0) vs time upon perfusion of 2.8 mM glucose Krebs buffer (~150 s), followed by the addition of 25 µM yoda1 and ending with 70 mM K^+ depolarizing buffer in *Piezo1*-silenced cells versus non-targeted siRNA treatment (si-Ctrl).

b. The average of area under the curve (AUC) for yoda1 stimulation in *Piezo1*-silenced cells versus si-Ctrl in (**a**) (n=63 cells, $p<0.0001$).

c. As in (**a**) but comparing negative control to *Piezo2* silencing.

d. As in (**b**) but comparing negative control to *Piezo2* silencing (n=56 cells).
e. $[Ca^{2+}]_i$ in human β-cells superfused with medium containing 2.8 mM glucose or 16.7 mM glucose (G) as indicated and following the addition of yoda1 (red) or solvent (DMSO) (black). Yoda1 was introduced in the medium at t=400 s.
f. ΔAUC for the increase in response to yoda1 (red) compared to control (DMSO alone; black) (n=27 and 31 cells for control and yoda1, respectively, p=0.0192)
g. Representative traces upon perfusion of 2.8 mM glucose (~100 s), 16.7 mM glucose (~300 s) and followed by addition of 50 µM yoda1 or DMSO as control in 2.8 mM glucose Krebs buffer in dispersed rat islet cells. The cells are assumed to be β-cells bases on their responsiveness to high glucose.
h. The cells responding to 16.7 mM glucose were analyzed for the average of area under the curve (AUC) for yoda1 stimulation in **e** (n=58 cells for each condition, p<0.0001).
Data are presented as mean values ± S.E.M. Statistical significance were evaluated using two-tailed unpaired Student's *t*-test in **b**, **d**, **f**, **h**, *p<0.05, ****p<0.0001.

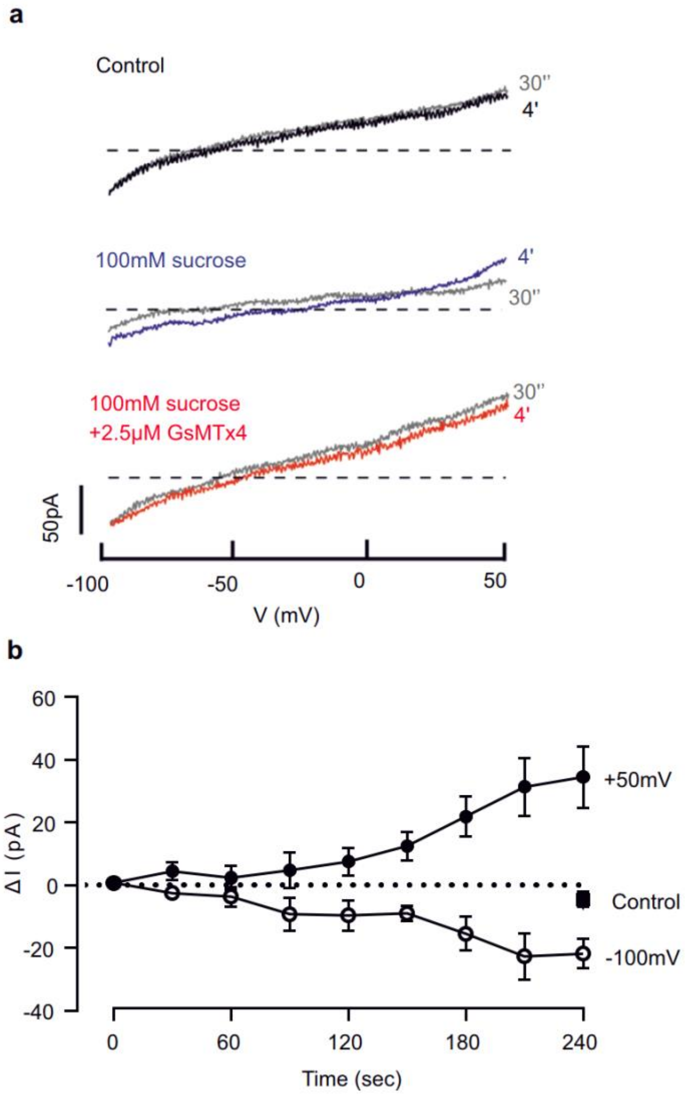

Figure S7. Swelling-induced currents in primary mouse β-cells.

a. Whole-cell currents of β-cells during 100-ms voltage ramps between -100 and 50 mV recorded ⩽30 s and 4 min after establishment after the whole-cell configuration in cells infused with normal intracellular solution (control, n=4; top) or intracellular solution supplemented with 100 mM sucrose (n=5; middle) in the presence of 2.5μM GsMTx4 in the extracellular solution (n=5; lower).

b. Time course of subtracted currents (as in main Figure 4o) at -100mV and +50mV at the time of establishment of the whole-cell configuration (t=0 s) and at later times (n=5 cells). Squares show changes in membrane currents at -100 and +50 mV over 4 min in control cells (no sucrose; n=4). Mean values ± S.E.M.

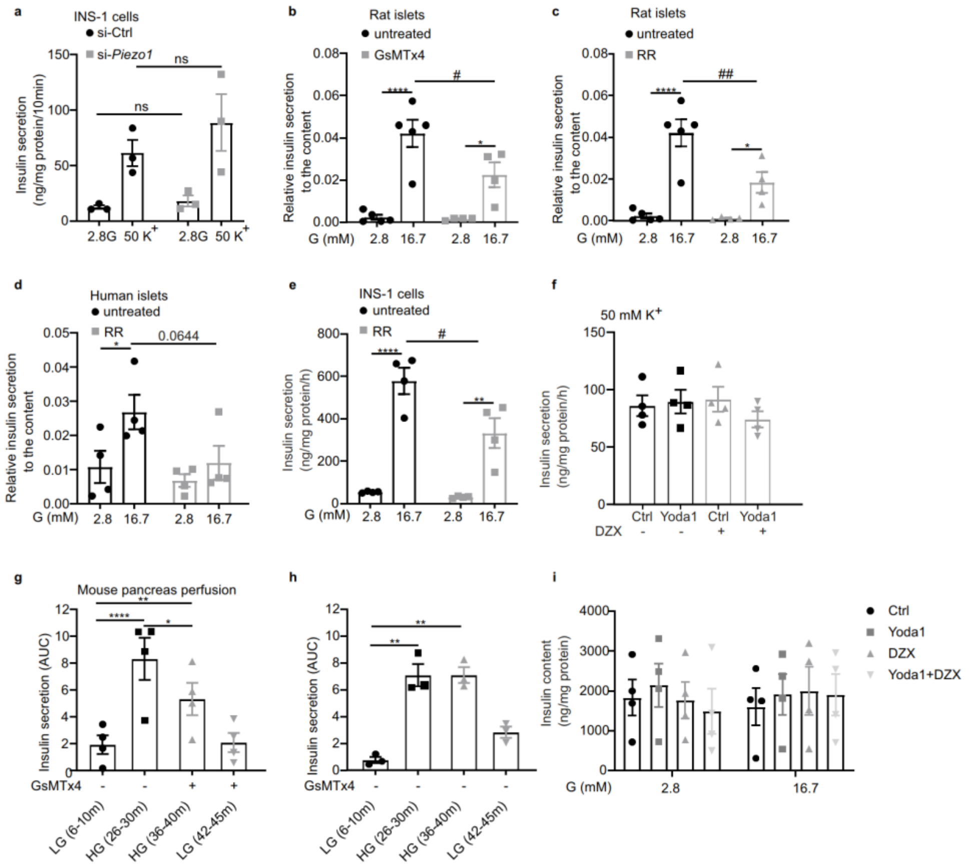

Figure S8 Effects of inhibition of Piezo1 on glucose-stimulated insulin secretion.

a. Insulin secretion in INS-1 832/13 cells in 2.8 mM glucose or 50 mM K^+ after silencing of *Piezo1* versus si-Ctrl (n=3).

b. Insulin secretion in rat islets in 2.8 mM or 16.7 mM glucose with normal buffer (n =5) or buffer with GsMTx4 (n =4), p values for the comparison between 2.8 and 16.7 mM G without/with GsMTx4 were <0.0001, 0.017, respectively, that between untreated and GsMTx4 treatment under 16.7 mM G was 0.015.

c. Insulin secretion in rat islets in 2.8 mM or 16.7 mM glucose with normal buffer (n =5) or buffer with Ruthenium Red (RR, n=4), p values for the comparison between 2.8 and 16.7 mM G without/with RR were <0.0001, 0.0375, respectively, that between untreated and RR treatment under 16.7 mM G was 0.0034.

d. As in **c** but in human islets (n=4), p values for the comparison between 2.8 and 16.7 mM G in untreated islets was 0.045, respectively, that between untreated and RR treatment under 16.7 mM G was 0.0644.

e. As in **c** but in INS-1 832/13 cells (n=4), p values for the comparison between 2.8 and 16.7 mM G without/with RR were <0.0001, 0.0033, respectively, that between untreated and RR treatment under 16.7 mM G was 0.014.

f. Insulin secretion in INS-1 832/13 cells in the presence of 50 mM K^+ in the absence and presence of yoda1 (25 μM) and/or diazoxide (200 μM; DZX) as indicated (n =4).

Diazoxide and yoda1 were dissolved in DMSO; control media contained the same amount of DMSO.
g. The total insulin release in perfusion of pancreas in the time period of t = 6-10, 26-30, 36-40 (with GsMTx4) and 42-45 min (with GsMTx4), respectively in **Figure 5d** (stippled areas; n=4), p values for the comparison between LG (6-10m) and HG (26-30m), HG (36-40m), were <0.0001, 0.0052, respectively, that between HG (26-30m) and HG (36-40m) was 0.011.
h. As in **g** but for the experiment treated without GsMTx4 as indicated in **Figure 5e** (stippled areas; n=3), p values for the comparison between LG (6-10m) and HG (26-30m), HG (36-40m), were 0.001, 0.001, respectively.
i. Insulin content related to **Figure 5c** in Ctrl, yoda1, DZX, and yoda1+DZX treated cells (n=4).
Data are presented as mean values ± S.E.M. Statistical significances were evaluated by two-way ANOVA multiple comparisons, except for one-way ANOVA multiple comparisons in **f**, *, the significance compared between 2.8 mM and 16.7 mM glucose, #, the significance compared between treatments with and without pharmacological drugs. All statistical tests used were two-sided unless otherwise indicated. *p<0.05, **p<0.01, ****p<0.0001. ns: not significant.

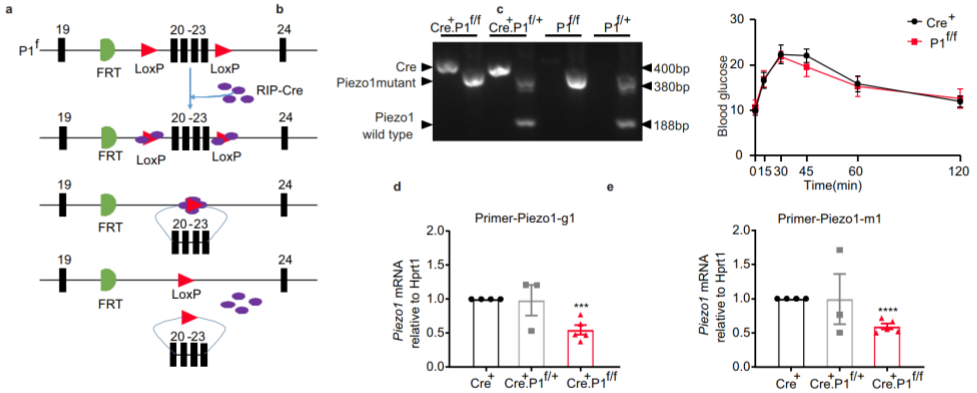

Figure S9 Verification of the β-cell specific *Piezo1* knock-out mice and IPGTT for Ctrl and floxed *Piezo1* mice.

a. The floxed and null alleles of *Piezo1* following recombination with RIP-Cre recombinase between LoxP sites (from exon 20 to 23), so part of Piezo1 from exon 19 to 24 was shown, FRT: FLP recombinase target.

b. Genotyping by PCR using tail biopsies from RIP-Cre^{+}.$P1^{f/f}$, RIP-Cre^{+}.$P1^{f/+}$, $P1^{f/f}$, $P1^{f/+}$ mice.

c. Blood glucose post-IPGTT in male RIP-Cre^{+} (black) or floxed Piezo1 mice (red) (n=6 and 3 for Cre^{+} and $P1^{f/f}$ mice, respectively).

d. Real-time qPCR analysis of *Piezo1* in isolated islets of RIP-Cre^{+}, Cre^{+}.$P1^{f/+}$, Cre^{+} $P1^{f/f}$ mice using primers targeting exons 21-22 (Primer-P1-g1). n=4, 3 and 5 for Cre^{+}, Cre^{+}.$P1^{f/+}$ and Cre^{+} $P1^{f/f}$ islets, respectively, p=0.0007.

e. As in (**c**) but using primers targeting exons 22-23 (Primer-P1-m1). n=4, 3 and 5 for Cre^{+}, Cre^{+}.$P1^{f/+}$and Cre^{+} $P1^{f/f}$ islets, respectively, p<0.0001.

Data are presented as mean values ± S.E.M. Statistical significances were evaluated by two-tailed unpaired Student's t-test in **d** and **e**, ***p<0.001, ****p<0.0001.

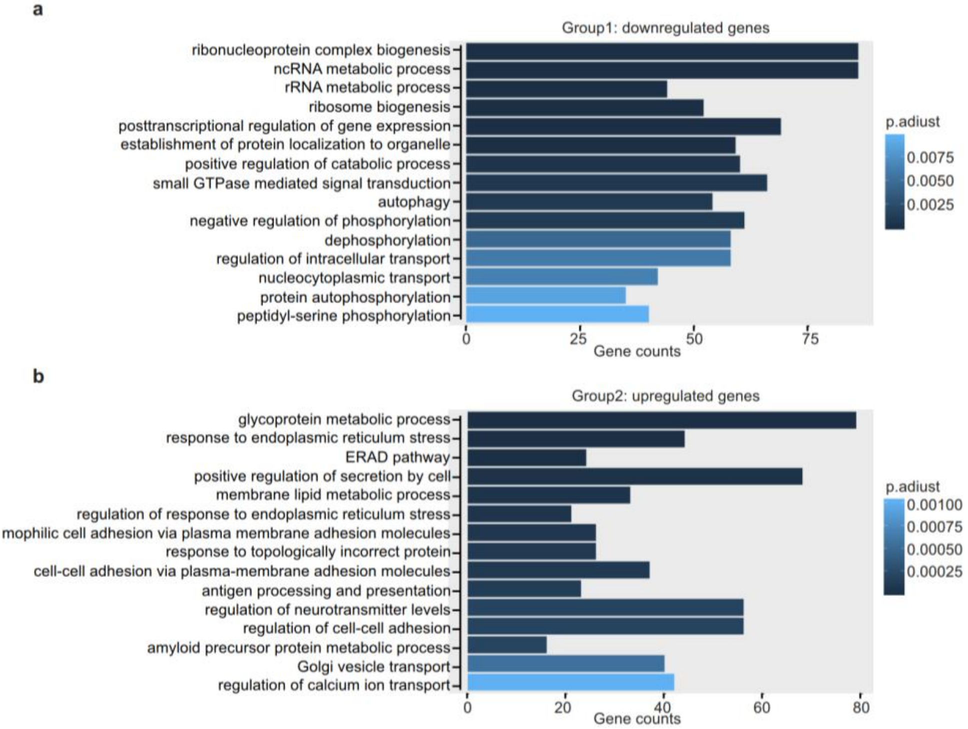

Figure S10.Gene Ontology terms enrichment for RNA-sequencing in *Piezo1*-silenced cells.

a. The most downregulated genes that involved in the indicated signal pathways.

b. Same as in **a**, but the signal pathways involved the most upregulated genes.

Supplemental Table 1

Human donor information						
**Gender**	**Age**	**BMI**	**HbA1c(%)**	**Blood group**	**Non-diabetic**	**T2D**
Male	57	26.4	5.5	A	1*	0*
Male	39	29	4.2	A	1	0
Female	53	28.7	5.2	AB	1	0
Female	68	18.7	5.5	A	1	0
Male	63	26	6.6	A	0	1
Male	66	19.7		O	1	0
Male	62	27.8	5.5	O	1	0
Male	78	20.1		A	1	0
Male	68	23.1	5.5	A	1	0
Female	54	33.7	5.8	B	1	0
Female	58	25.8	6.1	A	1	0
Male	64	27.2	5.5	A	1	0
Male	62	24	5.5	O	1	0
Male	62	34.9	6.4	A	0	1
Female	60	26.1	6.1	B	1	0
Male	68	27.8	6.4	O	1	0
Female	65	33.3	7.3	A	0	1
Male	45	23.5	7.3	A	0	1
Male	55	27.8	5.7	O	1	0
Male	63	18	5.7	O	1	0
Male	66	26.6	6.1	O	1	0
Male	67	26.1	5.6	O	1	0
Male	57	21.6	6.6	O	0	1
Male	61	37	6.1	O	1	0
Male	58	26.8	6	A	0	1
Male	65	23.1	5.6	A	1	0
Male	57	26.4	5.5	A	1	0

(*, 1 means yes and 0 means no)

Supplemental Table 2-4:

Differential gene expression was performed with Deseq2 (PMID: 25516281). Briefly, after normalization of the raw gene counts with a sample-specific size factor, normalized counts were modeled using the negative binomial distribution and the Wald test was performed on the model coefficients to compare gene expression between conditions. The p-values attained by the Wald test were corrected for multiple testing using the Benjamini-Hochberg method.

Supplemental Table 2

Differentially expressed genes in si-*Piezo1* samples			
**Rank**	**Gene_Name**	**log_2FoldChange**	**padj**
1	Slc40a1	5,711	0,01758
2	AABR07044397.1	4,564	0,035236
3	Cartpt	4,208	0
4	Lgals5	3,739	0
5	Sftpa1	3,703	0,011455
6	Gbp6	3,656	0,024215
7	NEWGENE_1306399	-3,514	0,006709
8	Gabre	3,493	0,012917
9	Lpar6	3,466	0,020677
10	Gast	3,444	0
11	RGD1559962	-3,368	0,029664
12	Krt78	3,163	0,027815
13	C1rl	2,863	0,027798
14	Crispld2	-2,851	0,033635
15	AABR07044631.2	2,81	0,002746
16	Ggt1	2,748	0,014224
17	AABR07008670.1	2,723	0,036213
18	Sds	2,719	0,000014
19	Fgg	2,677	0,016029
20	Aldh3b1	2,662	0
21	Ampd3	-2,656	0,007487
22	Pcdh10	2,649	0,013767
23	Gper1	2,604	0
24	LOC100359515	2,558	0,032669
25	AABR07018078.1	2,542	0
26	Spata31d3	-2,515	0,017123
27	Lamc3	-2,498	0,00069
28	Fgr	2,491	0,048021
29	AC120291.4	-2,489	0,048841
30	Gask1b	2,484	0,0266
31	Wnt10b	2,428	0,000471
32	Zim1	2,418	0,000026
33	Ntsr1	2,405	0
34	Chrna3	2,393	0,001141
35	Mlph	2,354	0,000333
36	Mmd2	2,344	0
37	Sun3	2,322	0,022555
38	Fbn2	2,317	0
39	Cfap73	2,292	0,046442
40	Ces1f	2,263	0,028431
41	AABR07034428.1	2,255	0,000013
42	Cyp1b1	2,255	0,000119
43	Calhm2	-2,22	0,000001
44	LOC497963	2,209	0,025294
45	Lcn1	2,197	0,000121
46	Calhm1	-2,178	0,019483
47	Esr1	2,154	0,010679
48	Col5a1	2,11	0,026281
49	Setdb2	2,108	0,007601
50	Dpp4	-2,082	0,0497
51	Ifi47	2,072	0
52	Mest	2,052	0
53	Pdgfrb	-2,046	0,001909
54	Fam151a	2,04	0
55	Prr35	-2,011	0,047072

Differentially expressed genes in si-*Piezo1* samples			
Rank	Gene_Name	log_2FoldChange	padj
56	Gbp7	1,993	0,003108
57	Chrnb4	1,957	0
58	Rgs5	1,939	0,00001
59	Cd7	1,923	0,000502
60	Ramp1	-1,918	0
61	Clcnkb	1,916	0,008117
62	Rxfp3	1,916	0,000071
63	Ptprb	1,894	0
64	Rgs4	1,882	0,008116
65	Ptn	1,88	0
66	Emp1	-1,866	0
67	P2rx2	1,839	0,000752
68	Asb4	1,838	0,007424
69	Pzp	1,835	0,000906
70	AABR07008293.4	1,822	0,006791
71	Gfra2	1,811	0,005506
72	Cemip	1,803	0,023876
73	Ckmt2	1,796	0
74	Mxra8	1,779	0,015809
75	Stap2	1,776	0,013268
76	Matn2	1,772	0
77	Tnni3k	1,754	0,000001
78	AABR07032097.1	1,737	0,008655
79	Gcnt3	1,733	0
80	Cmtm2a	1,669	0,000983
81	Chrm2	1,666	0,030028
82	Spef2	1,661	0,000315
83	Cd40	-1,593	0
84	F10	-1,593	0,020272
85	Spon1	1,586	0
86	Rbm44	-1,585	0,001722
87	Myom2	-1,559	0,000003
88	Rsph4a	1,551	0,009208
89	Atf3	-1,541	0
90	Parp14	1,536	0
91	MGC105567	1,534	0,000001
92	Trpm3	1,534	0,000009
93	Ucn3	1,522	0
94	Cngb1	1,515	0,000001
95	Sorcs1	1,501	0,00003
96	Usp18	1,498	0,000002
97	Fam129a	1,487	0
98	Fgb	1,486	0
99	Krt23	-1,478	0,000606
100	Ihh	-1,471	0

N.B. The gene counts of *Slc40a1* and *AABR07044397*.1 are much lower than that for *Cartpt*, therefore, *Cartpt* was selected as the candidate regulted by Piezo1.

Supplemental Table 3

Differentially expressed genes in si-*Piezo2* samples			
**Rank**	**Gene_Name**	**$log_2FoldChange$**	**padj**
1	Ugt2b35	1,81	0,000002
2	Pzp	1,587	0,009461
3	Ramp1	-1,481	0
4	LOC100912427	1,453	0,002422
5	AABR07049286.1	1,349	0,00162
6	Rbm44	-1,346	0,009706
7	Casp4	-1,34	0,011042
8	Dr1	-1,314	0,021767
9	Tspo2	-1,303	0,012928
10	Akap5	1,286	0,001964
11	Adgrg2	1,28	0,000001
12	Cartpt	1,278	0,021463
13	Hscb	-1,248	0
14	AABR07017104.1	-1,211	0,01267
15	Vim	-1,184	0,000967
16	Trpc5	1,174	0,011042
17	Uhmk1	1,148	0,003075
18	AABR07021096.1	1,143	0,003847
19	Krt23	-1,128	0,011546
20	Cdkl5	1,127	0,038318
21	Fst	1,111	0,004514
22	Ctrb1	-1,109	0,001787
23	AABR07015003.1	1,103	0,039181
24	Cd40	-1,098	0,000001
25	Nefm	-1,063	0
26	Barhl2	-1,06	0,040191
27	Cyp1a1	1,05	0,002238
28	Car8	-1,043	0
29	Vsig1	1,035	0,036856
30	Rnd1	-1,034	0
31	Vegp2	1,027	0,000009
32	Itih1	1,025	0,001801
33	Tgfbr3	1,024	0
34	Myom2	-1,011	0,002686
35	Col6a6	1,01	0,002462
36	Lrrc3b	-1,002	0,030878
37	C1s	1	0,000409
38	Ppm1f	0,996	0
39	Krt80	0,991	0,000002
40	Plcxd2	0,99	0,039837
41	Ephx4	-0,974	0,027793
42	Ldhb	-0,969	0
43	Guca2b	-0,954	0,000014
44	Nkrf	0,95	0,00162
45	Zfp483	0,949	0,035614
46	Prss53	0,939	0
47	Slc6a19	-0,938	0,000001
48	Npy1r	0,935	0,025565
49	Cntnap1	0,93	0,000129
50	LOC100910275	0,927	0,007359
51	AABR07026536.1	0,924	0,001067
52	Camk2a	-0,923	0,012841
53	Nr2f1	-0,916	0,016137
54	Plekhm3	0,912	0,028034
55	Ccdc190	-0,908	0,038538

Differentially expressed genes in si-*Piezo2* samples			
**Rank**	**Gene_Name**	**log_2FoldChange**	**padj**
56	Polr3g	0,904	0
57	Ugt2b37	0,894	0,000136
58	Gast	0,888	0,000091
59	Sytl4	0,887	0,000001
60	Pik3r3	0,886	0,004727
61	Igfbp2	-0,884	0,012517
62	Ccdc33	-0,879	0,043536
63	LOC297568	0,873	0,000099
64	Anxa8	-0,869	0,000017
65	Chrnb4	0,869	0,01248
66	Dtx1	-0,868	0
67	Otulinl	-0,864	0
68	Fam126a	0,856	0,045331
69	Gstm6	0,838	0,018226
70	Togaram2	-0,83	0,015962
71	Tet1	0,83	0,036115
72	Gria4	-0,829	0,043733
73	Misp3	-0,825	0
74	Meltf	0,819	0,026144
75	AABR07010878.1	0,818	0,04787
76	S100a3	-0,811	0,000321
77	S100a4	-0,81	0,000058
78	F13a1	0,808	0,027338
79	Ccdc28a	-0,808	0
80	Htra3	-0,807	0,000005
81	Gpr158	0,807	0,025494
82	Tcp11	-0,806	0,009413
83	Fam89a	-0,805	0,00029
84	LOC100909732	0,801	0,020675
85	AABR07001037.1	0,797	0,002453
86	Fyb1	0,795	0,000003
87	Svep1	0,793	0
88	Chia	-0,791	0,007406
89	Galnt9	-0,79	0
90	S100a5	-0,787	0
91	Sh3pxd2a	0,785	0,018344
92	Rasgrp2	-0,783	0,000025
93	Dusp26	-0,781	0,015707
94	Cd276	-0,779	0
95	Gpr88	-0,768	0
96	Rasa2	0,767	0,000131
97	Zbtb7c	0,762	0
98	C1r	0,76	0,000044
99	Cgnl1	0,751	0,017907
100	Osgin1	0,749	0,000152

Supplemental Table 4

Differentially expressed genes in si-*Piezo1+2* samples			
**Rank**	**Gene_Name**	**log_2FoldChange**	**padj**
1	Cartpt	3,591	0
2	AABR07044631.2	3,238	0,000533
3	Pzp	2,896	0
4	Gast	2,882	0
5	Pdgfrb	-2,608	0,000132
6	Aldh3b1	2,501	0,000001
7	AABR07034428.1	2,417	0,000003
8	Lcn1	2,417	0,000022
9	Chrna3	2,328	0,00219
10	Sds	2,29	0,000584
11	AABR07018078.1	2,224	0
12	Cyp1b1	2,02	0,001048
13	Cd7	1,994	0,000371
14	Lgals5	1,98	0,002635
15	Ugt2b35	1,978	0
16	Mest	1,943	0
17	Gper1	1,922	0
18	Ramp1	-1,91	0
19	Wfdc1	1,892	0,000579
20	P2rx2	1,889	0,000619
21	AABR07021096.1	1,858	0
22	Setdb2	1,789	0,039682
23	Lamc3	-1,785	0,005991
24	AABR07062138.2	1,747	0,008335
25	Fam151a	1,737	0
26	Wnt10b	1,732	0,029904
27	Mlph	1,73	0,019987
28	Msln	1,597	0
29	F2	1,563	0
30	C1s	1,551	0
31	Ntsr1	1,519	0,00148
32	Chrnb4	1,511	0
33	Zim1	1,495	0,02876
34	Calhm2	-1,468	0,000439
35	Cacng5	1,439	0,017475
36	Smim23	-1,431	0,03071
37	Rerg	1,401	0,010381
38	Dkk2	-1,391	0,00652
39	Mmd2	1,374	0
40	Morn5	1,364	0,020874
41	Fbn2	1,353	0,000005
42	Fkbp14	1,346	0,034633
43	Ptprb	1,34	0
44	Hk2	-1,328	0,008382
45	Ifi47	1,324	0
46	AABR07015003.1	1,324	0,007501
47	Csmd1	1,309	0,049169
48	Krt80	1,306	0
49	Tmc3	-1,292	0,014432
50	Piezo1	-1,29	0

Differentially expressed genes in si-*Piezo1+2* samples			
**Rank**	**Gene_Name**	**log2FoldChange**	**padj**
51	Plg	1,276	0,039707
52	Lrguk	1,271	0,049769
53	Atf3	-1,268	0
54	Cd40	-1,259	0
55	Col28a1	-1,241	0,040681
56	Meltf	1,227	0,000108
57	C1r	1,224	0
58	Myom2	-1,217	0,000118
59	Gcnt3	1,212	0
60	Rbm44	-1,208	0,016331
61	Emp1	-1,199	0
62	Elfn1	1,195	0,000006
63	Abcg2	1,194	0,048508
64	Smpdl3a	-1,183	0
65	Ucn3	1,153	0,000001
66	Dner	1,15	0
67	Oxtr	1,149	0,017314
68	Matn2	1,127	0
69	Pag1	1,113	0,046024
70	Gpr75	1,113	0,008996
71	Akap5	1,107	0,008117
72	LOC100361492	1,102	0,001376
73	Clic6	1,095	0,001816
74	Cald1	1,094	0,008086
75	S100a4	-1,093	0
76	Gna15	-1,088	0,000001
77	Parp14	1,086	0
78	Rab7b	-1,083	0,000011
79	Fst	1,082	0,004318
80	Caps2	1,065	0,027161
81	Svep1	1,063	0
82	Ugt2b37	1,049	0,000002
83	Vegp2	1,048	0,000003
84	Grik1	1,046	0,009124
85	Large2	1,023	0
86	Kitlg	-1,016	0,000004
87	Tekt1	1,011	0,007153
88	Acer1	-1,009	0,00074
89	Wfikkn2	1	0,025765
90	Galnt18	0,985	0
91	Piwil2	-0,978	0,001295
92	Glod5	0,972	0,000005
93	Hs3st2	0,964	0,000071
94	Cngb1	0,961	0,004965
95	Cx3cl1	-0,951	0
96	Snx31	-0,95	0,000001
97	Fhod3	-0,945	0
98	Tslp	0,934	0,018475
99	Efna1	-0,933	0,000034
100	Cyp1a1	0,933	0,006231
