# Supplementary material for: A critical role of the mechanosensor PIEZO1 in glucose-induced insulin secretion in pancreatic β-cells
Source: Nat Commun. 2022 Jul 22;13:4237. doi: 10.1038/s41467-022-31103-y (PMC9307633; doi:10.1038/s41467-022-31103-y)
Supplement: Supplementary file 2 — Reporting Summary [file 41467_2022_31103_MOESM2_ESM.pdf]

nature portfolio

Corresponding author(s): Erik Resntrom, Enming Zhang

Last updated by author(s): 2/18/2

Reporting Summary

Nature Portfolio wishes to improve the reproducibility of the work that we publish. This form provides structure for consistency and transparency in reporting. For further information on Nature Portfolio policies, see our Editorial Policies and the Editorial Policy Checklist.

Statistics

For all statistical analyses, confirm that the following items are present in the figure legend, table legend, main text, or Methods section.

n/a	Confirmed	
☐	☒	The exact sample size (*n*) for each experimental group/condition, given as a discrete number and unit of measurement
☐	☒	A statement on whether measurements were taken from distinct samples or whether the same sample was measured repeatedly
☐	☒	The statistical test(s) used AND whether they are one- or two-sided<br>*Only common tests should be described solely by name; describe more complex techniques in the Methods section.*
☒	☐	A description of all covariates tested
☐	☒	A description of any assumptions or corrections, such as tests of normality and adjustment for multiple comparisons
☐	☒	A full description of the statistical parameters including central tendency (e.g. means) or other basic estimates (e.g. regression coefficient) AND variation (e.g. standard deviation) or associated estimates of uncertainty (e.g. confidence intervals)
☐	☒	For null hypothesis testing, the test statistic (e.g. *F*, *t*, *r*) with confidence intervals, effect sizes, degrees of freedom and *P* value noted<br>*Give P values as exact values whenever suitable.*
☒	☐	For Bayesian analysis, information on the choice of priors and Markov chain Monte Carlo settings
☒	☐	For hierarchical and complex designs, identification of the appropriate level for tests and full reporting of outcomes
☐	☒	Estimates of effect sizes (e.g. Cohen's *d*, Pearson's *r*), indicating how they were calculated

Our web collection on statistics for biologists contains articles on many of the points above.

Software and code

Policy information about availability of computer code

Data collection	PatchMaster software (version 2x91) was used to collect the electrophysiology data; Cell size measurement was done by Image J software (version 1.52a); The fluorescence from Fura-2 AM was monitored at 340/ 380 nm excitation and 510 nm emission using a Polychrome V high-speed switching monochromator on a Nikon microscope equipped with an Andor ER-BOB-100 trigger box, an Andor camera Ixon3, and IQ2 software. For RNA-seq data analysis, genes were grouped according to their expression profiles using hierarchical clustering with the R package DEGreport (v.1.21.1). GO terms enrichment was performed using the R package ClusterProfiler (v. 3.13.0). Western blot data were analyzed and quantified with the Image-Lab 5.2.1 program.
Data analysis	The data analysis was done in GraphPad Prism 7 software.

For manuscripts utilizing custom algorithms or software that are central to the research but not yet described in published literature, software must be made available to editors and reviewers. We strongly encourage code deposition in a community repository (e.g. GitHub). See the Nature Portfolio guidelines for submitting code & software for further information.

Data

Policy information about availability of data

All manuscripts must include a data availability statement. This statement should provide the following information, where applicable:

- Accession codes, unique identifiers, or web links for publicly available datasets
- A description of any restrictions on data availability
- For clinical datasets or third party data, please ensure that the statement adheres to our policy

The authors declare that all data supporting the findings of this study are available within the article and its Supplementary Information Files or from the corresponding author on reasonable request. All resource data underlying relevant panels in Fig. 1-7, and Supplementary Fig. XX are provided with this publication.

Field-specific reporting

Please select the one below that is the best fit for your research. If you are not sure, read the appropriate sections before making your selection.

☒ Life sciences ☐ Behavioural & social sciences ☐ Ecological, evolutionary & environmental sciences

For a reference copy of the document with all sections, see nature.com/documents/nr-reporting-summary-flat.pdf

Life sciences study design

All studies must disclose on these points even when the disclosure is negative.

Sample size	Sample size was determined based on standard practices of field of physiology and cell biology. In general, at least three independent experiments were carried out for further statistical analysis. Accordingly, all the bar/chart showing in the study represent at least 3 repeats.
Data exclusions	Except where experiments failed because of technical issues, no data was excluded.
Replication	Each experiment was preformed multiple times under exact conditions in different models for example INS-1 cells lines (experiments were repeated weekly because of 96h transfection), primary islets (the experiments were repeated when the islets are available, for rodent islets that can be achieved frequently are repeated every 2-3 days or at least weekly, for human islets that we can't control the time for receiving them, we can only repeat the experiments when we can get them). Some patch data were not included in the study due to control experiments were not performed well.
Randomization	The passages of cell lines were randomly used for the experiments, the same passage of cell line was used for each experiment. The islets were also collected all at a time for each experiment and then assigned to different conditions randomly using a certain number of islets (e.g. 8 islets/well/condition were used for insulin secretion, 3 technical replicates/condition were applied, 50-100 islets were used for immunostaining). The treatment conditions such as reagents concentration and treatment time were fully controlled.
Blinding	The evaluation of results were all based on data achieved by software analysis and calculations, investigators also honestly reported the results. The assignment of samples to each condition was random and cell lines, primary islets especially rodent ones are relatively less in variation than human. Therefore, investigator blinding is less relevant to our study than in clinical trials.

Reporting for specific materials, systems and methods

We require information from authors about some types of materials, experimental systems and methods used in many studies. Here, indicate whether each material, system or method listed is relevant to your study. If you are not sure if a list item applies to your research, read the appropriate section before selecting a response.

Materials & experimental systems

n/a	Involved in the study	
☐	☒	Antibodies
☐	☒	Eukaryotic cell lines
☒	☐	Palaeontology and archaeology
☐	☒	Animals and other organisms
☐	☒	Human research participants
☒	☐	Clinical data
☒	☐	Dual use research of concern

Methods

n/a	Involved in the study	
☒	☐	ChIP-seq
☒	☐	Flow cytometry
☒	☐	MRI-based neuroimaging

Antibodies

Antibodies used	Primary antibodies against Piezo1 (1:200, #15939-1-AP, Proteintech), insulin (1:400, #16049, Progen) or glucagon (1:200, #ab10988, Abcam), β-actin (1:1000, #A2228, Sigma), anti-rabbit or mouse horseradish peroxidase (HRP)-conjugated secondary antibodies (1:1000, #7074, Cell Signaling Technology and #P0447, Dako), anti-rabbit Alexa Flour 488 secondary antibody (711-545-152, Jackson ImmunoResearch), anti-mouse cy3 (715-165-151, ImmunoResearch), anti-guinea pig Alexa647 (706-165-148, Jackson ImmunoResearch)
Validation	It was ascertained that the PIEZO antibody did not label beta-cell in Piezo1 knockout islets pointing out the specificity of PIEZO1 antibody (Fig6a.b and Supplemental Fig.S4).<br>Insulin (#16049, Progen) for beta-cell immunostaining was validated by our lab (Zhang et el 2019 Cell metabolism) and other group (Al-Amily, I. et al 2019 Pflugers.Arch).<br>Glucagon (#ab10988, Abcam) was validated for immunostaining in alpha-cell by manufacturer and it is one of the mostly used antibody for glucagon detection. As shown on the company website, the antibody was referred to by 140 publications (https://www.abcam.com/glucagon-antibody-k79bb10-ab10988.html).

β-actin (#A2228, Sigma) is one of the mostly used antibody for actin detection. The actin in cells of various species and tissue origin is very similar in their immunological and physical properties. As a consequence, it has been difficult to produce potent antisera to this protein. Therefore the availability of monoclonal antibodies to β-actin provides a specific and useful tool in studying the intracellular distribution of β-actin and the static and dynamic aspects of the cytoskeleton. This antibody has been diversely used in IF, WB, IHC, etc and was referred to by 2485 peer reviewed papers (https://www.sigmaaldrich.com/US/en/search/a2228?focus=papers&page=1&perPage=30&sort=relevance&term=A2228&type=citation_search).

Eukaryotic cell lines

Policy information about cell lines

Cell line source(s)	INS-1 832/13 cells was originally received from Professor Newgard CB lab. The cells derived from parental INS-1 cells and express the human proinsulin gene [Hohmeier, H.E. et al. Isolation of INS-1-derived cell lines with robust ATP-sensitive K+ channel-dependent and -independent glucose-stimulated insulin secretion. Diabetes 49, 424-430 (2000)]. The cells have been preserved and cultured in house at our department since delivery in the 00's.
Authentication	The cell line was not authenticated.
Mycoplasma contamination	The cell line was not tested for mycoplasma contamination. This is done, however, whenever the cells' behavior or culture deviates from normal.
Commonly misidentified lines (See ICLAC register)	No commonly misidentified cell lines were used in the study.

Animals and other organisms

Policy information about studies involving animals; ARRIVE guidelines recommended for reporting animal research

Laboratory animals	β-cell-specific Piezo1 knockout mice (RIP-Cre+.Piezo1f/f) were generated by mating mice expressing the Cre recombinase gene under the control of rat insulin 2 gene promoter (RIP-Cre+) 48 with Piezo1tm2.1Apat/J (also known as P1f) (Stock #029213, The Jackson Laboratory) mice to obtain RIP-Cre+. Piezo1f/+ mice, which were then crossed with P1f to get RIP-Cre+.P1f/f knockout (KO) mice. Male and age of 5-8 weeks'RIP-Cre+.P1f/f knockout (KO) mice were used in the study.<br>Wistar rats:<br>Islets isolated from male Wistar rats (≥11 weeks old) were used. A minimum of 3 rats were used in each group.<br>db/db and C57BL/6J mice:<br>Islets were isolated from male db/db mice and age-matched control male C57BL/6J mice (≥12 weeks of age). At least 3 mice were used in each group.<br>All the animals were kept in a pathogen-free facility on a 12–12 hr light–dark cycle at temperature of 22 celsius degrees with humidity of 55%.
Wild animals	The study did not use any wild animals.
Field-collected samples	The study did not involve any samples collected from the field.
Ethics oversight	Rats and mice used were approved by the Malmö/Lund and Gothenburg Animal Care and Use Committee and abided by the guide for the care and use of the laboratory animals published by the Directive 2010/63/EU of the European Parliament. Animals were allocated to experimental groups by genotype/diabetic phenotype. All the animal experiments were conducted according to ARRIVE guidelines.

Note that full information on the approval of the study protocol must also be provided in the manuscript.

Human research participants

Policy information about studies involving human research participants

Population characteristics	The RNA-seq data were analyzed from 181 donors (114 healthy, 40 pre-diabetes, 27 diabetes). Of these, islets from 12 healthy (8 males + 4 females) and 1 diabetic (male) donors were used for experiments. More information about the donors was listed in Supplemental Table 1.
Recruitment	Pancreatic islets/human tissues (fat, liver and muscle) were obtained from the Nordic Network for Islet Transplantation (Uppsala University, Sweden) via EXODIAB Human Tissue Lab in Lund University with the approval of the ethics committees at Uppsala and Lund. The islets/ tissues were selected according to the HbA1c values (<5.5 as non-diabetes, between 5.5 and 6 as IGT, impaired glucose tolerance, >6 as diabetes) and purity of the islets, we tried to use those islets with higher than 85% purity and hand-pick the islets for experiment to minimize the possible contamination from exocrine tissues.
Ethics oversight	Informed consent was obtained from pancreatic donors or their relatives in accordance with the approval by the local ethics committee at Uppsala (Uppsala Regional Ethics Board)and Lund Universities regarding organ donation for medical research (Lund-Malmö Ethical committee).

Note that full information on the approval of the study protocol must also be provided in the manuscript.
