# Supplementary material for: A critical role of the mechanosensor PIEZO1 in glucose-induced insulin secretion in pancreatic β-cells
Source: Nat Commun. 2022 Jul 22;13:4237. doi: 10.1038/s41467-022-31103-y (PMC9307633; doi:10.1038/s41467-022-31103-y)
Supplement: Supplementary file 3 — Peer Review File [file 41467_2022_31103_MOESM3_ESM.pdf]

nature portfolio

Peer Review File

A critical role of the mechanosensor PIEZO1 in glucose-induced insulin secretion in pancreatic beta-cells

Open Access This file is licensed under a Creative Commons Attribution 4.0 International License, which permits use, sharing, adaptation, distribution and reproduction in any medium or format, as long as you give appropriate credit to the original author(s) and the source, provide a link to the Creative Commons license, and indicate if changes were made. In the cases where the authors are anonymous, such as is the case for the reports of anonymous peer reviewers, author attribution should be to 'Anonymous Referee' followed by a clear attribution to the source work. The images or other third party material in this file are included in the article's Creative Commons license, unless indicated otherwise in a credit line to the material. If material is not included in the article's Creative Commons license and your intended use is not permitted by statutory regulation or exceeds the permitted use, you will need to obtain permission directly from the copyright holder. To view a copy of this license, visit http://creativecommons.org/licenses/by/4.0/.

Reviewers' Comments:

Reviewer #1:
Remarks to the Author:
General comments
In this paper, the authors investigated the roles of the mechanosensor Piezo1 in pancreatic β-cells. They show that localization of Piezo1 changes depending on the extracellular glucose concentration and that Piezo1 regulates intracellular Ca2+ homeostasis and controls insulin secretion, suggesting that regulation of insulin secretion by Piezo1 is altered in diabetic β-cells. Although several studies have reported that mechanistic stimulation such as changes in blood vessel tension and β-cell swelling by glucose-induced hypertonicity affects insulin secretion, as mentioned in their introduction, the present study is the first to show the involvement of Piezo1 in the pathophysiology of the diabetic β-cell. Considering that a variant at the PIEZO1 locus is associated with HbA1c in East Asians, this study is potentially of interest pathophysiologically. However, the authors do not provide any data on the mechanism of Piezo1 activation by mechanical stimuli (β-cell swelling?). Without such data, it is too preliminary to propose the model in Figure 5i at this stage. Substantial investigation is required to propose the model. The data on the association between Piezo1 and Swell1 are also lacking. In addition, some of the data do not support of the authors' interpretation. The authors should reconcile the inconsistent findings and interpretations.

Specific comments.
1. Lines 11-15 on page 8. The authors state "Overall, … and indicates the importance of the mechanosensitive action of Piezo1 for sensing glucose uptake, therefore, supporting the conclusion of Piezo1 as a mechanosensor sensing glucose-induced cell swelling, rather than membrane depolarization to influence insulin secretion.insulin secretion." However, no direct evidence for Piezo1 sensing of glucose uptake or glucose-induced cell swelling is given in this paper. The authors investigated the effects of Piezo1 activation and inactivation by using its agonist or inhibitor and by knockdown and knockout. How is Piezo1 activated physiologically by mechanical stimuli? Does hypertonicity (or hypotonicity) induce activation of Piezo1? Is the activation of Piezo1 by mechanical stimulation mediated by Swell1, or do Piezo1 and Swell1 have distinct roles? Does Piezo1 require both glucose metabolism and β-cell swelling to induce membrane depolarization (or to induce [Ca2+]i increase)? The authors should address these issues.

2. Localization of Piezo1.
The authors show that Piezo1 localized at the cell membrane is reduced by chronic high glucose exposure, and that it is also reduced in β-cells of diabetic mice (Figure 2). On the other hand, the total expression level of Piezo1 is elevated in diabetic β-cells, even though the PIEZO1 protein level is not significantly increased in human islets after chronic high glucose exposure (Figure 1). Since quantification by immunofluorescence indicates the relative distribution of the protein, it seems difficult to conclude that the absolute amount of Piezo1 localized at the plasma membrane differs among different metabolic states. Some additional evaluations could strengthen the conclusion (for example, western blot analysis of each fraction of the β-cell or electron microscopic analysis, etc.). What was the glucose concentration used for culturing db/db islets? Related to this point, does chronic culture of islets and beta-cell lines in which high glucose is normally used affect sublocalization of Piezo1?

3. Difference in the localization of Piezo1 between α- and β-cells.
The authors show a difference in the localization of Piezo1 protein between α- and β-cells and interpret this as different roles of Piezo1 in α- and β-cells (Figure 1 and lines 20-22 on page 4). As glucagon and insulin show an opposite response to the glucose concentration, the Piezo1 in α-cells may simply reflect this difference. The authors might want to discuss the role of Piezo1 in α-cells more specifically.

4. Fig S2. It is difficult to understand the authors' interpretation of this figure. The Piezo1 2458-2547-GFP is re-distributed to cytosol in response to high glucose. According to this result, the authors state "…echoing the behavior of wild type Piezo1. These results suggest that C-terminal inner helix part of Piezo1 (aa2458-2547) is required for correct intracellular trafficking and its metabolic regulation". However, Piezo1 2458-2547-GFP should be translocated to nucleus in response to high glucose, based on the results of figure 2. The authors should explain this point.

5. Figure. 4.
Fig. 4g. Please specify "NC", e.g., non-targeting siRNA or non-treated cells.
Fig. 4h-j. The authors should provide the insulin secretion data expressed as absolute values (not relative to control) and the data on insulin content as well.
Fig. 4i-j. Judging from the result, it seems that the Piezo1 effect on insulin secretion is significantly diminished after diazoxide treatment. This suggests that the Piezo1 effect requires KATP channel closure to some extent, which argues against the authors' conclusion (lines 15-16 on page 7). The authors should modify their statements. Again, the data expressed as fold change (relative to control) are not convincing (see above).

6. Conditional Piezo1 KO mice.
Figure 5e, f show that glucose-induced [Ca2+]i increase depends on the expression level of Piezo1, whereas glucose tolerance of heterozygous KO mice is improved compared with control Cre+ mice, possibly due to the impaired glucose tolerance of Cre+ mice mentioned in the manuscript. What about insulin secretion from the islets of heterozygous and homozygous KO mice or serum insulin levels during glucose tolerance test? These experiments would be important to reach the conclusion that reduced membrane-anchored Piezo1 affects insulin secretion in the diabetic state.

7. Line 10 on page 3. Reference 6 (Zatelli et al., J Clin Endocrinol Metab) seems inappropriate. There is no description of blood vessel tension induced by free fatty acids in the paper.

Reviewer #2:
Remarks to the Author:
The manuscript by Yingying Ye et al. determines how the mechanosensitive channel Piezo1 influences beta-cell calcium entry and insulin secretion. The manuscript finds that Piezo1 knockdown reduces beta-cell calcium currents, glucose-induced calcium entry and insulin secretion. Interestingly, Piezo1 is shown to increase voltage-dependent calcium channel currents under depolarized potentials, however, Piezo1 did not influence calcium entry or insulin secretion from beta cells during depolarization-induced by high potassium. The manuscript goes on to conclude that Piezo1 control of beta cell membrane potential regulates glucose-induced calcium entry and insulin secretion. Although the manuscript shows that Piezo1 influences calcium entry and insulin secretion, there is no assessment of the role of Piezo1 on beta cell membrane potential. Therefore, there are some issues that require attention, which are specified below.

Major,
The regulation of voltage-gated calcium channel currents by PIEZO1 under voltage clamped conditions has not been shown before and is interesting. The authors state that Peizo1 expression correlates with changes in the expression of CACNA1C, CACNA1D and CACNA1H. The manuscript fails to look if knockdown or knockout of Piezo1 changes any calcium channel transcript, which is required. The manuscript also shows that Piezo1 co-localizes with CaV1.3 channels (at least 20% of the CaV1.3 channels) and may thus control CaV1.3 activity. The authors claim that, "These results agree with previous report that shear stress causes depolarization and activate voltage-gated Ca2+ channels in the adjacent vascular smooth muscle cells [23].This is compatible with the idea that Piezo1 mediates the basal depolarizing tone in β-cells." However, as the VDCC currents are enhanced during depolarizing steps with voltage clamp electrophysiology, this statement is not exactly correct because the membrane potential is clamped. While the VDCC control by Piezo1 is interesting, the manuscript requires some mechanism for how Piezo1 control of VDCCs. Co-localization is not enough to indicate that interactions of Piezo1 with CaV1.3 impacts channel function.

VDCC currents are significantly reduced from beta cells with Piezo1 knockdown compared to controls in response to equivalent depolarizing voltage steps using the voltage clamp technique (differences in VDCC currents are observed from -10 mV through 40 mV voltage steps). This strongly predicts that clamping the membrane voltage at a depolarized potential with high potassium should also change calcium entry through VDCC channels at least during the peak of

the action potentials resulting from this condition. However, the manuscript finds that high potassium-induced depolarization causes equivalent calcium entry and insulin secretion from beta cells with or without Piezo1 expression and/or islets with PIEZO activation or inhibition. Therefore, the data are confusing because Piezo1 regulation of calcium channel activity independently of its control of membrane potential (data shown in figure3i) should result in differences in high potassium changes in calcium and insulin secretion when Piezo1 levels or function is different. Are the authors trying to show that Piezo1 control of VDCCs impacts beta cell function and/or that Piezo1 controls the membrane potential? The authors' claims about Piezo1 control of membrane potential should definitely be substantiated with some membrane potential recordings.

When YODA was first identified it was shown to increase calcium influx into cells through PIEZO1 (see PMID: 26001275). Thus YODA could potentially increase calcium influx in low glucose conditions through direct activation of PIEZO1 calcium entry. The authors claim that, "We explored if activation of Piezo1 in beta-cells leads to membrane depolarization using [Ca2+]i imaging." However, as KATP would be active under low glucose conditions, YODA may not result in enough of a depolarizing current to activate VDCC currents. Therefore, the authors need to determine if the YODA influences on calcium entry and insulin secretion under low glucose conditions are due to depolarization mediated VDCC activation or just PIEZO1 mediated calcium entry. This could be accomplished though membrane potential recordings; if there are no action potentials elicited with YODA under low glucose conditions, then VDCCs are not activated.

Some confirmation that the antibody staining is only marking PIEZO1 is required. This could easily be performed in beta cells and alpha cells from piezo deficient islets or SiRNA treated cells. Note that control islet cells or control SiRNA treated cells that are stained at the same time and imaged with the same microscope settings should also be included in this antibody control experiment.

The mouse insulin secretion shown in Figure 4 panel c seems abnormal in that there is no first or second phase of secretion. This differs from the labs previous measurements with the same technique using mice on a C57Bl/6 background (for example see PMID: 15630454). Some explanation for the lack of first phase insulin secretion is requires.

Insulin secretion from the piezo deficient islets should be included.

Minor:
A size marker for the western blots should be included. Otherwise it is hard to determine the molecular weight of the bands shown.

The x access on the graph in figure 5g does not appropriately display the time. The 0-5 minute time interval is shown is the same width as the 60-120 minute interval. This needs to be adjusted.

Reviewer #3:
Remarks to the Author:
In this manuscript, Ye et al. provided some interesting findings that Piezo1, a mechanically-activated cation channel, regulates insulin secretion. Although there is potential novelty implicating a mechanosensor in beta cell biology, the results are too preliminary which limits the overall impact and significance. Are beta cells mechanosenstive? Do mechanical forces induce currents in beta cells? Are they mediated by Piezo1? What kinds of mechanical force beta cells experience under physiological conditions? Are they strong enough to activate Piezo1 channel? These are the key questions linking mechanotransduction to beta cell biology. The authors appears to suggest blood flow and shear stress could activate Piezo1, however, it's unclear beta cells can directly experience shear like endothelial cells do. The authors also suggested that glucose-induced beta cell swelling may activate Piezo1. But the swelling is very limited (~10% even at 20 mM gluacose perfusion). There is no elecrophysiological evidence that high glucose can activate Piezo1 channel in beta cells.

Other points on experiment design and reagents:

1. The antibody for Piezo1 immunostaining in Figure 1, 2 and 3 needs careful validation. Piezo1, like many other membrane proteins, are notoriously difficult to do immunostaining due to non-specificity. The broad and strong Piezo1 signals in Figure 1 and 2 do appear to be nonspecific staining, especially given the very low levels of Piezo1 mRNA expression in the islet. Proper controls with KO cells are necessary. Piezo1 is a membrane protein. What exactly does it mean to translocate to nucleus, presumably still in a membrane bound form? From the pictures, it's not possible to determine the membrane localization of Piezo1 from cytosolic localization. In addition, if the antibody performs so well, the authors should examine tissue sections so they can have a better idea on Piezo1 expression in difference cell types in the islet, their relative subcellular localization, its response to glucose, Piezo1 KO mice cell type specificity etc.

2. The critical in vivo experiments (Figure 5) are confusing and not conclusive partly due to the serious issues with RIP-cre line as noted by others in the field and the authors (leakage in other cell types, and human growth factor minigene http://www.jbc.org/content/281/5/2649.full). MIP-cre or CreER should be used, not only for the cell specificity, also making sure the phenotype is not due to the deletion of Piezo1 during beta cell development.

Reviewers' comments:

Reviewer #1 (Remarks to the Author):

Comment 1: In this paper, the authors investigated the roles of the mechanosensor Piezo1 in pancreatic β-cells. They show that localization of Piezo1 changes depending on the extracellular glucose concentration and that Piezo1 regulates intracellular Ca2+ homeostasis and controls insulin secretion, suggesting that regulation of insulin secretion by Piezo1 is altered in diabetic β-cells. Although several studies have reported that mechanistic stimulation such as changes in blood vessel tension and β-cell swelling by glucose-induced hypertonicity affects insulin secretion, as mentioned in their introduction, the present study is the first to show the involvement of Piezo1 in the pathophysiology of the diabetic β-cell. Considering that a variant at the PIEZO1 locus is associated with HbA1c in East Asians, this study is potentially of interest pathophysiologically.

Response: We were pleased to see that this Reviewer recognizes that this study is the first to address the role of PIEZO1 in the pathophysiology of diabetes.

Comment 2: However, the authors do not provide any data on the mechanism of Piezo1 activation by mechanical stimuli (β-cell swelling?). Without such data, it is too preliminary to propose the model in Figure 5i at this stage. Substantial investigation is required to propose the model.

Response: These are very relevant questions. These have been addressed by new experiments showing: (i) that high glucose causes cell swelling (Figure S5); (ii) that shear stress induces insulin secretion (Figure 3h); (iii) that hypotonicity stimulates insulin secretion (Figure 3g).

The involvement of PIEZO1 in these reactions was demonstrated by using the PIEZO1 inhibitor GsMTx4 (Figure 3g).

GsMTx4 reduced resting membrane potential and Ca^{2+} signaling induced by hypotonicity (Figure 3a-b, e-f). As a consequence of this, GsMTx4 also decreased hypotonicity-stimulated insulin secretion (Figure 3g).

With these newly added crucial results, we think we provide sufficient evidence for the model we outline in Figure 8 and that also incorporates the effects of type 2 diabetes.

Comment 3: The data on the association between Piezo1 and Swell1 are also lacking. In addition, some of the data do not support of the authors' interpretation. The authors should reconcile the inconsistent findings and interpretations.
Response: To see whether SWELL1 participates in PIEZO1-activated Ca^{2+} signaling, we measured intracellular Ca^{2+}. Our results show that silencing of *Piezo1* alone, or double knockdown of *Piezo1* and *Swell1*, had similar effects to that of silencing of *Swell1* alone, on hypotonicity-induced $[Ca^{2+}]_i$ oscillations (Figure S4). This suggests that PIEZO1 and SWELL1 operate in parallel and that activation of either of these channels suffice to stimulate insulin secretion and/or increase Ca^{2+}. A statement to this effect has been inserted (ll 116-119 and ll 285-287)

Specific comments.
Comment 4: (Point 1). Lines 11-15 on page 8. The authors state "Overall, … and indicates the importance of the mechanosensitive action of Piezo1 for sensing glucose uptake, therefore, supporting the conclusion of Piezo1 as a mechanosensor sensing glucose-induced cell swelling, rather than membrane depolarization to influence insulin secretion.insulin secretion."

These are highly relevant questions that are identified by the latter's a-e as indicated below:

[a] However, no direct evidence for Piezo1 sensing of glucose uptake or glucose-induced cell swelling is given in this paper. The authors investigated the effects of Piezo1 activation and inactivation by using its agonist or inhibitor and by knockdown and knockout.

[b] How is Piezo1 activated physiologically by mechanical stimuli?

[c] Does hypertonicity (or hypotonicity) induce activation of Piezo1?

[d] Is the activation of Piezo1 by mechanical stimulation mediated by Swell1, or do Piezo1 and Swell1 have distinct roles?

[e] Does Piezo1 require both glucose metabolism and β-cell swelling to induce membrane depolarization (or to induce [Ca2+]i increase)?

Response to **[a], [e]**

We interpret these questions as the reviewer would like to see the following chain of events clarified:

Glucose uptake → Beta-cell swelling → PIEZO1 activation →Beta-cell metabolism →Insulin secretion

It has previously been demonstrated that glucose induces beta-cell swelling [1-3]. In our hands, we also observed that cell size increased by >6% under perfusion of high glucose (16.7 mM) compared to that in 2.8 mM glucose (Figure S5). In fact, these published observations were the starting point for this study and here we have focused on the downstream events.

We show that equimolar of the non-metabolizable sugar mannitol for glucose fails to increase cytoplasmic Ca^{2+} (unlike what is seen with glucose itself) (Figure 4a-c).

By contrast, silencing of *Piezo1* in INS-1 832/13 cells or beta-cell specific KO of *Piezo1* impaired glucose-induced electrical activity (Figure 4l-m & 6h-j), intracellular Ca^{2+} signaling (Figure 4a & c, Figure 6f-g) and insulin secretion (Figure 5b & 6e). Likewise, the PIEZO1 inhibitor GsMTx4 impaired glucose-induced Ca^{2+} signaling (Figures 4h-i, S6g-h) and insulin secretion (Figures 5a,d,h, S7a,f) in INS-1 832/13 cells, mouse, rat and human islets.

These results clearly demonstrate that PIEZO1, across species, senses uptake and metabolism of glucose, as well as affects the sequence of reactions culminating in insulin secretion.

[b] [c] Physiologically, glucose stimulation can result in either beta-cell swelling upon glucose stimulation (see references[1-3]), or by changes in islet blood flow through the richly vascularized islets. Islet blood flow is normally much higher than in the surrounding exocrine tissue and coupled to the need for insulin secretion (see referene [4]).

To address whether PIEZO1 can be activated by cell swelling, we reduced the osmolarity of the extracellular solution in different experiments. INS-1 832/13 cells exposed to hypotonic stimulation responded with increased cytoplasmic Ca^{2+} (Figure 3a-d), induction of electrical activity (Figure 3e-f) and stimulation of insulin secretion (Figure 3g). All these effects were reversed by the PIEZO1 inhibitor GsMTx4.

Finally, as stated above, pancreatic islets are richly vascularized and the blood flow exhibits great variation *in vivo* [4].To mimic this, we applied *in vitro* shear stress to cultured INS-1 832/13 cells and

measured insulin secretion. Shear stress stimulated insulin secretion but the latter effect, unlike that of hypotonicity, was not affected by GsMTx4 (Figure 3h).

Taken together, these data show that PIEZO1 only mediates the effects of hypotonicity/swelling but not that of shear stress.

[d] See response to Comment 3 above.

[e] Concerning the contribution of beta-cell metabolism to swelling-induced insulin secretion the following data helps to clarify the view:

Treatment with the PIEZO1 agonist yoda1 under low-glucose conditions induces beta-cell electrical activity (Figure 4j-k). This shows that the PIEZO1-dependent mechanism stimulating insulin secretion – under these conditions – appears to act independently of beta-cell metabolism.

Treatment with the K_{ATP} channel opener diazoxide also prevented the stimulatory effect of yoda1 on glucose-induced insulin secretion (Figure 5c).

Taken together, these results show that PIEZO1-related effects on insulin secretion are enhanced by simultaneously occurring glucose metabolism (Figure 5c). Fully activated K_{ATP} channels (mimicking a low-energy state) keep the beta-cells in a hyperpolarized state and largely prevent PIEZO1 from activating the beta-cell. Thus, we fully agree with the Reviewer's comment that "the Piezo1 effect requires K_{ATP} channel closure to some extent" but we think this role is permissive and that PIEZO1 does not directly influence K_{ATP} channel activity.

Comment 5: (Point 2). Localization of Piezo1. The authors show that Piezo1 localized at the cell membrane is reduced by chronic high glucose exposure, and that it is also reduced in β-cells of diabetic mice (Figure 2). On the other hand, the total expression level of Piezo1 is elevated in diabetic β-cells, even though the PIEZO1 protein level is not significantly increased in human islets after chronic high glucose exposure (Figure 1).

The question falls into two parts, which are identified as **[a]** and **[b]** as follows:

[a] Since quantification by immunofluorescence indicates the relative distribution of the protein, it seems difficult to conclude that the absolute amount of Piezo1 localized at the plasma membrane differs among different metabolic states. Some additional evaluations could strengthen the conclusion (for example, western blot analysis of each fraction of the β-cell or electron microscopic analysis, etc.).

[b] What was the glucose concentration used for culturing db/db islets? Related to this point, does chronic culture of islets and beta-cell lines in which high glucose is normally used affect sublocalization of Piezo1?

Responses:

[a] The concern of the reviewer that the reduced plasma membrane expression of PIEZO1, as demonstrated by immunofluorescence, does not necessarily reflect an absolute reduction in PIEZO1 expression, is valid. However, for different reasons, this point is nearly impossible to address with available methods. EM with immunogold labeling is also a relative assessment, and the same is in reality also true for the cellular fractionation experiments.

To make the best of this we have made additional experiments to study PIEZO1 cellular localizations in whole mouse islets cultured with 5 mM, 10 mM and 20 mM glucose for 72 h. Using high-resolution

confocal imaging, we observed nearly identical patterns of intracellular distributions of PIEZO1 in single beta-cells dispersed from the cultured islets as in Figure 2 (Figure S1c). These confocal images in mouse beta-cells, human beta-cells under a series of conditions and treatments, all point to the same patterns of subcellular distributions of PIEZO1 and the same effects by culture under hyperglycemic conditions. We therefore feel confident that the localization of PIEZO1 is very sensitive to changes in glucose concentrations.

[b] As to the effects of glucose on PIEZO1 localization in *db/db* mouse beta cells, we have made a fuller investigation on the recovery of PIEZO1 distribution after standard glucose (10 mM) incubation (Figure 2g-h). First, as shown in Figure 2e-f, we cultured both C57 black mouse and *db/db* mouse dispersed islet cells with 10 mM glucose overnight prior to immunostaining. The *db/db* mice had severe hyperglycemia (fed plasma glucose: >25 mM) before being sacrificed (to be compared with <10 mM in control mice). Our data demonstrate that short-term culture (overnight) is unable to significantly change the PIEZO1 intracellular distribution. For this, more long-term (72 h) culture is required, after which nuclear PIEZO1 localization shifts from the nucleus to the cytosol and plasma membrane area, probably restoring normal PIEZO1 function in the plasma membrane (Figure 2g-h).

Comment 6: (Point 3). Difference in the localization of Piezo1 between α- and β-cells.
The authors show a difference in the localization of Piezo1 protein between α- and β-cells and interpret this as different roles of Piezo1 in α- and β-cells (Figure 1 and lines 20-22 on page 4). As glucagon and insulin show an opposite response to the glucose concentration, the Piezo1 in α-cells may simply reflect this difference. The authors might want to discuss the role of Piezo1 in α-cells more specifically.

Response: This is indeed an interesting aspect. However, the role of PIEZO1 in α-cells is a huge topic that probably requires a separate study and we are therefore obliged to conclude that such experiments are 'beyond the scope of this study' (ll.74-77) and would require several years of experiments. We hope that the Reviewer finds the approach reasonable.

Comment 7: (Point 4). Fig S2. It is difficult to understand the authors' interpretation of this figure. The Piezo1 2458-2547-GFP is re-distributed to cytosol in response to high glucose. According to this result, the authors state "…echoing the behavior of wild type Piezo1. These results suggest that C-terminal inner helix part of Piezo1 (aa2458-2547) is required for correct intracellular trafficking and its metabolic regulation". However, Piezo1 2458-2547-GFP should be translocated to nucleus in response to high glucose, based on the results of figure 2. The authors should explain this point.

We appreciate that the way these experiments were described may have been confusing. We have reworded this section as follows: "These results suggest that the C-terminal inner helix part of PIEZO1 (aa2458-2547) is required for the intracellular trafficking of PIEZO1. Additional experiments are required to identify the exact residue(s)/protein regions responsible for sensing the metabolic state and translocation to the nucleus. "(ll. 101-104). However, it is clear that this work would form a basis for future initiatives to pharmacologically correct the defective intracellular trafficking of PIEZO1 in beta-cells from T2D patients (see ll. 292-294)

Comment 8. (Point 5). Figure. 4. Fig. 4g. Please specify "NC", e.g., non-targeting siRNA or non-treated cells.
Fig. 4h-j. The authors should provide the insulin secretion data expressed as absolute values (not relative to control) and the data on insulin content as well.
Fig. 4i-j. Judging from the result, it seems that the Piezo1 effect on insulin secretion is significantly

diminished after diazoxide treatment. This suggests that the Piezo1 effect requires KATP channel closure to some extent, which argues against the authors' conclusion (lines 15-16 on page 7). The authors should modify their statements. Again, the data expressed as fold change (relative to control) are not convincing (see above).

Response: These are all very good points and we have amended the manuscript as suggested:

Fig 4g (NOW Fig. 5b). "NC" has been changed accordingly to "si-Ctrl" which means transfection with non-targeting siRNA.

Fig. 4h-j (NOW Fig. 5c) We agree and that is now presented as absolute values. Data on insulin content are shown in the Supplementary material as requested (Figure S7i).

The statement about PIEZO1 and K_{ATP} channel closure pathway has been modified to avoid the seeming contradiction (see also Response to the question **[e]** of Comment 4 above).

Comment 9: (Point 6). Conditional Piezo1 KO mice.
Figure 5e, f show that glucose-induced [Ca2+]i increase depends on the expression level of Piezo1, whereas glucose tolerance of heterozygous KO mice is improved compared with control Cre+ mice, possibly due to the impaired glucose tolerance of Cre+ mice mentioned in the manuscript. What about insulin secretion from the islets of heterozygous and homozygous KO mice or serum insulin levels during glucose tolerance test? These experiments would be important to reach the conclusion that reduced membrane-anchored Piezo1 affects insulin secretion in the diabetic state.

For clarity, we now only show Ctrl and KO data and have omitted results from the heterozygous mice. We now show data on the effects of ablating *Piezo1* on glucose-induced insulin secretion *in vitro* (Figure 6e). In addition, we have been able to complement the characterization of the *Piezo1* knockout mice with measurements of beta-cell electrical activity (new Figure 6h-j; ll. 230-241)

Comment 10. (Point 7). Line 10 on page 3. Reference 6 (Zatelli et al., J Clin Endocrinol Metab) seems inappropriate. There is no description of blood vessel tension induced by free fatty acids in the paper.

Thanks for pointing it out. This information has been deleted from the manuscript.

Reviewer #2 (Remarks to the Author):

The manuscript by Yingying Ye et al. determines how the mechanosensitive channel Piezo1 influences beta-cell calcium entry and insulin secretion. The manuscript finds that Piezo1 knockdown reduces beta-cell calcium currents, glucose-induced calcium entry and insulin secretion. Interestingly, Piezo1 is shown to increase voltage-dependent calcium channel currents under depolarized potentials, however, Piezo1 did not influence calcium entry or insulin secretion from beta cells during depolarization-induced by high potassium. The manuscript goes on to conclude that Piezo1 control of beta cell membrane potential regulates glucose-induced calcium entry and insulin secretion. [B1a]Although the manuscript shows that Piezo1 influences calcium entry and insulin secretion, there is no assessment of the role of Piezo1 on beta cell membrane potential. Therefore, there are some issues that require attention, which are specified below.

We thank the Reviewer for the nice summary of our work.

Major,

Comment 1: The regulation of voltage-gated calcium channel currents by PIEZO1 under voltage clamped conditions has not been shown before and is interesting. The authors state that Peizo1 expression correlates with changes in the expression of CACNA1C, CACNA1D and CACNA1H. The manuscript fails to look if knockdown or knockout of Piezo1 changes any calcium channel transcript, which is required. The manuscript also shows that Piezo1 co-localizes with CaV1.3 channels (at least 20% of the CaV1.3 channels) and may thus control CaV1.3 activity. The authors claim that, "These results agree with previous report that shear stress causes depolarization and activate voltage-gated Ca2+ channels in the adjacent vascular smooth muscle cells [23].This is compatible with the idea that Piezo1 mediates the basal depolarizing tone in β-cells." However, as the VDCC currents are enhanced during depolarizing steps with voltage clamp electrophysiology, this statement is not exactly correct because the membrane potential is clamped. While the VDCC control by Piezo1 is interesting, the manuscript requires some mechanism for how Piezo1 control of VDCCs. Co-localization is not enough to indicate that interactions of Piezo1 with CaV1.3 impacts channel function.

Response 1: We agree that more work is needed to address the control of VDCCs by PIEZO1. There was also a discrepancy between the INS-1 832/13 cells and human islet data that we have been unable to resolve. Following revision, this aspect has become somewhat peripheral to the manuscript's narrative and we have therefore decided to omit it until we are in a position to provide a more definitive mechanism.

The role of PIEZO1 is more tightly linked to the initiation of electrical activity. We have now conducted fluorimetric recordings of membrane potential using PMPI. These show that the glucose-stimulated depolarization is significantly impaired after silencing of *Piezo1* (Figure 4l-m). By membrane potential recordings in intact islets from beta-cell specific *Piezo1* KO mice, glucose-stimulated electrical activity is also severely reduced (Figure 6h-j). By contrast, tolbutamide remained stimulatory in *Piezo1* knockout mice (suggesting the existence/contribution of additional depolarizing mechanisms). Thus, activation of PIEZO1 is specific for glucose and upstream of depolarization-evoked activation of the voltage-gated Ca^{2+} channels.

Comment 2: VDCC currents are significantly reduced from beta cells with Piezo1 knockdown compared to controls in response to equivalent depolarizing voltage steps using the voltage clamp

technique (differences in VDCC currents are observed from -10 mV through 40 mV voltage steps). This strongly predicts that clamping the membrane voltage at a depolarized potential with high potassium should also change calcium entry through VDCC channels at least during the peak of the action potentials resulting from this condition. However, the manuscript finds that high potassium-induced depolarization causes equivalent calcium entry and insulin secretion from beta cells with or without Piezo1 expression and/or islets with PIEZO activation or inhibition. Therefore, the data are confusing because Piezo1 regulation of calcium channel activity independently of its control of membrane potential (data shown in figure3i) should result in differences in high potassium changes in calcium and insulin secretion when Piezo1 levels or function is different.

This is a very valid point and we agree it was confusing. As discussed above (Response 1), revision has necessitated some changes of the conclusions and this has – as discussed above -resulted in less focus on the voltage-gated calcium channels and this aspect is no longer part of the study.

Comment 3:]Are the authors trying to show that Piezo1 control of VDCCs impacts beta cell function and/or that Piezo1 controls the membrane potential? The authors' claims about Piezo1 control of membrane potential should definitely be substantiated with some membrane potential recordings.

Response: We agree. Such data are now provded (see Response to Comment 1 above).

Comment 4: When YODA was first identified it was shown to increase calcium influx into cells through PIEZO1 (see PMID: 26001275). Thus YODA could potentially increase calcium influx in low glucose conditions through direct activation of PIEZO1 calcium entry. The authors claim that, "We explored if activation of Piezo1 in beta-cells leads to membrane depolarization using [Ca2+]i imaging." However, as KATP would be active under low glucose conditions, YODA may not result in enough of a depolarizing current to activate VDCC currents. Therefore, the authors need to determine if the YODA influences on calcium entry and insulin secretion under low glucose conditions are due to depolarization mediated VDCC activation or just PIEZO1 mediated calcium entry. This could be accomplished though membrane potential recordings; if there are no action potentials elicited with YODA under low glucose conditions, then VDCCs are not activated.

Response 4: This is a very insightful comment on an important issue. To address this, we monitored the membrane potential during addition of yoda1, the new data is added to Figure 4j-k.

Treatment with the PIEZO1 agonist yoda1 under low-glucose conditions induces beta-cell electrical activity (Figure 4j-k). This shows that the PIEZO1-dependent mechanism stimulating insulin secretion –without further glucose metabolism and K_{ATP} channel closure - generates electrical activity with VGCC action potential.

Treatment with the K_{ATP}-channel opener diazoxide can counteract the stimulatory effect of yoda1 on glucose-induced insulin secretion (Figure 5c). It should be noted that the glucose dependence of insulin secretion occurs at lower glucose concentrations in rat and human islets than in mouse islets and that the K_{ATP} channels are partially inhibited at 2.8 mM glucose. This might explain why we don't observe any stimulation of insulin secretion in perfused mouse pancreas at 2.8 mM glucose, despite yoda1 producing a transient increase in $[Ca^{2+}]_i$ in rat and human beta-cells (Figure 4f and Figure S6e).

Taken together, these results suggest that stimulation of insulin secretion by PIEZO1-activation can occur without further beta-cell metabolism (Figure 5c), but also that full activation of K_{ATP} channels prevent this and keep the beta-cells in a hyperpolarized state (see also Response to the question **[e]** of Comment 4 above to Reviewer 1).

Comment 5: Some confirmation that the antibody staining is only marking PIEZO1 is required. This could easily be performed in beta cells and alpha cells from piezo deficient islets or SiRNA treated cells. Note that control islet cells or control SiRNA treated cells that are stained at the same time and imaged with the same microscope settings should also be included in this antibody control experiment.

Response 6: We have verified the PIEZO1 antibody specificity in beta-cell specific knockout mice (Figure 6b) and *Piezo1*-silenced INS-1 832/13 cells (Figure S3b-d), measured by confocal imaging and Western blotting. A comment to this effect has been inserted in Methods (ll. 373, 406).

Comment 7: The mouse insulin secretion shown in Figure 4 panel c seems abnormal in that there is no first or second phase of secretion. This differs from the labs previous measurements with the same technique using mice on a C57Bl/6 background (for example see PMID: 15630454). Some explanation for the lack of first phase insulin secretion is requires.

Response 7: The representative curves are replaced by the average of repeated experiments shown in Figure 5d-e, in which the phasic insulin response is evident.

Comment 8: Insulin secretion from the piezo deficient islets should be included.

Response 8: These data are now presented and show that ablation of *Piezo1* results in diminished glucose-induced insulin secretion whilst not affecting basal secretion (Figure 6e).

Minor:
Comment 9: A size marker for the western blots should be included. Otherwise it is hard to determine the molecular weight of the bands shown.

Response 9: Corrected as suggested. The size marker has been added to the western blot images in Figure 1c.

Comment 10The x access (axis?) on the graph in figure 5g does not appropriately display the time. The 0-5 minute time interval is shown is the same width as the 60-120 minute interval. This needs to be adjusted.

Response 10: The x-axis has been adjusted by a proper time interval in Figure 6c.

Reviewer #3 (Remarks to the Author):

In this manuscript, Ye et al. provided some interesting findings that Piezo1, a mechanically-activated cation channel, regulates insulin secretion. Although there is potential novelty implicating a mechanosensor in beta cell biology, the results are too preliminary which limits the overall impact and significance.

We thank for the Reviewer for commenting on the novelty of the study. The critique on the preliminary nature of the data has been taken on board and we have extensively revised the manuscript.

Comment 1: Are beta cells mechanosenstive? Do mechanical forces induce currents in beta cells? Are they mediated by Piezo1? What kinds of mechanical force beta cells experience under physiological conditions? Are they strong enough to activate Piezo1 channel? These are the key questions linking mechanotransduction to beta cell biology.

The authors appears to suggest blood flow and shear stress could activate Piezo1, however, it's unclear beta cells can directly experience shear like endothelial cells do. The authors also suggested that glucose-induced beta cell swelling may activate Piezo1. But the swelling is very limited (~10% even at 20 mM gluacose perfusion). There is no elecrophysiological evidence that high glucose can activate Piezo1 channel in beta cells.

Response 1: We thank the reviewer for his/her feedback. This comment highlights a number of key questions. We deal with these questions in turn as detailed below:

Are beta cells mechanosenstive? We demonstrate that hypotonicity and shear stress induces insulin secretion in INS-1 832/13 cell (Figure 3g-h). Hypotonicity stimulates Ca^{2+} signaling (Figure 3a-d), membrane depolarization (Figure 3e-f). Together, with new online report [3], this demonstrates convincingly that beta-cells really are mechanosensitive.

Do mechanical forces induce currents in beta cells? Are they mediated by Piezo1? As explained in the preceding section, beta-cells are mechanosensitive and respond to mechanical stimuli as hypotonicity and shear stress by induction of insulin secretion. This stimulation involves PIEZO1, as evidenced by the sensitivity to GsMTx4 (Figure 3g). We provide evidence that the effects of hypotonicity on Ca^{2+} are prevented by silencing *Piezo1* (Figure 3c-d) or GsMTx4 (Figure 3a-b).

What kinds of mechanical force beta cells experience under physiological conditions? Are they strong enough to activate Piezo1 channel? The Reviewer's comment has made us insert a discussion of the swelling induced by glucose in beta-cells (ll. 139-144). We show that high glucose increases cell area by >6%, similar to what has been observed previously [1-3].

The model (Figure 8) has been revised to illustrate the link between glucose, glucose metabolism, beta-cell swelling and PIEZO1 activation. We provide direct evidence that PIEZO1 is required for glucose-induced (but not tolbutamide-induced) membrane depolarization (Figure 6h-j). As discussed this suggests the existence of additional depolarizing conductances (ll. 255-257)

We acknowledge that we have not been able to record the PIEZO1 currents; presumably because they are too small to be resolved. However, because the beta-cell input resistance is very high, even small currents can be expected to produce sufficient depolarization to evoke regenerative electrical activity. A statement to this effect has been inserted (ll. 166-170).

The authors appears to suggest blood flow and shear stress could activate Piezo1, however, it's unclear beta cells can directly experience shear like endothelial cells do.

We have reconsidered how to present this aspect in the revised version. Figure 3h shows that shear stress indeed triggers insulin release and that this effect is independent of PIEZO1 (as suggested by resistance to GsMTx4). By contrast, PIEZO1 is required for hypotonicity-induced insulin secretion (Figure 3g). This might be attributed to the membrane tension generated by hypotonicity, which generates a membrane stretch that effectively activates PIEZO1 [8]. From the data now presented, it seems reasonable to conclude that beta-cells are indeed able to sense shear stress, but the underlying mediator remains elusive and needs to be further explored. By contrast, cell swelling as generated by glucose metabolism is effectively sensed by PIEZO1.

Other points on experiment design and reagents:

Comment 2: The antibody for Piezo1 immunostaining in Figure 1, 2 and 3 needs careful validation. Piezo1, like many other membrane proteins, are notoriously difficult to do immunostaining due to non-specificity. The broad and strong Piezo1 signals in Figure 1 and 2 do appear to be nonspecific staining, especially given the very low levels of Piezo1 mRNA expression in the islet. Proper controls with KO cells are necessary.

Response: We have verified the PIEZO1 antibody in beta-cell specific knockout mice (Figure 6b) and *Piezo1*-silenced INS-1 832/13 cells (Figure S3b-d), assessed by confocal imaging and Western blotting (see Methods ll. 373, 406).

Comment: Piezo1 is a membrane protein. What exactly does it mean to translocate to the nucleus, presumably still in a membrane-bound form? From the pictures, it's not possible to determine the membrane localization of Piezo1 from cytosolic localization.

Response: This is an interesting question that we have tried to address in closer detail. Our observations simply reflect the glucose-dependent redistribution of PIEZO1-immunoreactivity in islet beta-cells (Figure 2 and new data in Figure S1c). We have tried to identify the certain domains involved in the nuclear distribution of PIEZO1 (Figure S2).

We have addressed the significance of nuclear translocation by analysis of differential expression by RNA-sequencing in *Piezo1*-silenced cells (Figure S9). For example, 58 genes in the regulation of intracellular transport and 42 genes in nucleocytoplasmic transport were downregulated after silencing of *Piezo1*, these can be candidates for further study of the mechanism of PIEZO1 redistribution under different stimuli.

In addition, if the antibody performs so well, the authors should examine tissue sections so they can have a better idea on Piezo1 expression in difference cell types in the islet, their relative subcellular l

To examine PIEZO1 expression, and its glucose dependence, in different islet cell types, we applied whole islet immunostaining after culturing islets in 5, 10 or 20 mM glucose for 72 h. The results are in line with the findings in the previous manuscript version, to the effect that PIEZO1 translocates to the nucleus under high glucose treatment (new data in Figure S1c).

Comment 3. The critical in vivo experiments (Figure 5) are confusing and not conclusive partly due to the serious issues with RIP-cre line as noted by others in the field and the authors (leakage in other cell types, and human growth factor minigenehttp://www.jbc.org/content/281/5/2649.full). MIP-cre

or CreER should be used, not only for the cell specificity, also making sure the phenotype is not due to the deletion of Piezo1 during beta cell development.

Response: We thank this reviewer for bringing up this important issue. The study referred to shows that the effect on blood glucose in the RIP-cre mice is predominant in female, but very slight in male mice (Figure 1 in the ref paper [9]). In our study, we use male mice and observe a low degree of variation among all RIP-cre. We have also compared blood glucose excursions after IPGTT between RIP-cre and floxed *Piezo1* mice, without any noticeable major differences (see attached data). For the sake of clarity, we only compare Ctrl and *Piezo1* KO mice in the revised version of the manuscript.

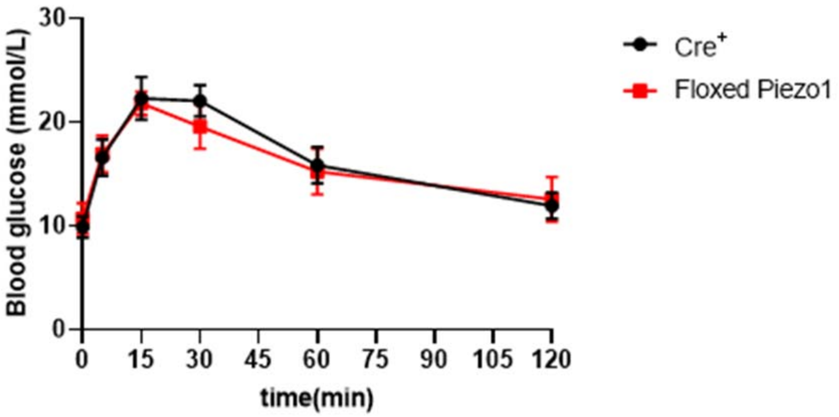

1.Semino, M.C., et al., Early changes in the rat pancreatic B cell size induced by glucose. Acta Anat (Basel), 1990. 138(4): p. 293-6.

2.Miley, H.E., et al., Glucose-induced swelling in rat pancreatic beta-cells. J Physiol, 1997. 504 (Pt 1): p. 191-8.

3.Kang, C., et al., SWELL1 is a glucose sensor regulating beta-cell excitability and systemic glycaemia. Nat Commun, 2018. 9(1): p. 367.

4.Jansson, L., et al., Pancreatic islet blood flow and its measurement. Ups J Med Sci, 2016. 121(2): p. 81-95.

5.Rode, B., et al., Piezo1 channels sense whole body physical activity to reset cardiovascular homeostasis and enhance performance. Nat Commun, 2017. 8(1): p. 350.

6.Schulla, V., et al., Impaired insulin secretion and glucose tolerance in beta cell-selective Ca(v)1.2 Ca2+ channel null mice. EMBO J, 2003. 22(15): p. 3844-54.

7.Deivasikamani, V., et al., Piezo1 channel activation mimics high glucose as a stimulator of insulin release. Sci Rep, 2019. 9(1): p. 16876.

8. Ridone, P., M. Vassalli, and B. Martinac, Piezo1 mechanosensitive channels: what are they and why are they important. Biophys Rev, 2019. 11(5): p. 795-805.

9.Lee, J.Y., et al., RIP-Cre revisited, evidence for impairments of pancreatic beta-cell function. J Biol Chem, 2006. 281(5): p. 2649-53.

Reviewers' Comments:

Reviewer #2:
Remarks to the Author:
The manuscript by Yingying Ye et al. has been improved with the revisions, which have addressed my previous concerns. The manuscript provides important mechanistic understanding of how the mechanosensitive channel Piezo1 influences beta-cell calcium entry and insulin secretion.

Reviewer #3:
Remarks to the Author:
Ye et. al. now proposed a model in which glucose metabolism induces beta cell swelling, which in turn activates the mechanosenor Piezo1 channel. Piezo1 activation mediates calcium influx and membrane depolarization, which leads to voltage-gated calcium channel (VGCC), secondary calcium influx and insulin secretion. The author presented some additional data to support this model. However, the key evidence supporting Piezo1 as a mechanosenor and responding to beta cell swelling in glucose-stimulated insulin secretion is still largely missing.

1. Piezo1 channel is activated by lipid bilayer membrane tension. In contrast to intuition, mild hypotonic swelling in mammalian cells actually does not generate strong membrane tension because mammalian cells have membrane reserve and invaginations. So far, it has not been reported in the literature that Piezo1 channel is activated by hypotonic cell swelling. Considering glucose only induces very mild beta cells swelling (5-10%), the new claim that Piezo1 channel is activated under this condition requires evidence. The authors speculated that the Piezo1 currents were too small to be electrophysiologically recorded. It's not clear whether such experiments were tried. If that's the case, the authors should overexpress Piezo1 and then record under high glucose perfusion. If beta cell recording is technically difficult, recording in a heterologous expression system should be performed to provide at least some indirect evidence.

2. In addition to the Piezo1 current, the mechanically-activated calcium influx is another important physiological readout. However, the calcium imaging experiments in the manuscript were performed in the bulk beta cells/islets over a longer time scale. Although they are conventional in the field, these large calcium signals are mainly due to the opening of VGCC, but not the proposed Piezo1-mediated calcium influx. This distinction is important and need to emphsized. Given the claim that Piezo1-mediated calcium influx induces membrane depolarization, it's critical to investigate the very initial VGCC-independent (using VGCC blocker?) calcium influx in much shorter time scale and single beta cell resolution.

3. The relationship between Piezo1 and Swell1 channels is puzzling. They were proposed to be activated by cell swelling but cause membrane depolarization with completely different mechanisms: Piezo1-calcium (positive ion) influx; SWELL1-choride (negative ion) efflux. The authors should discuss the potential reasons why no additive effect of knocking down both pathways is observed.

4. Accumulating evidence suggests that Piezo1 is a multi-function protein, which may regulate cellular function in non-conventional/mechanical ways. Indeed, the authors presented nice profiling data that Piezo1 knockdown changes the expression of hundreds of genes. These changes could at least in part explain the phenotype such as insulin secretion the authors observed. Therefore, it's important to discuss this and keep an open mind on how Piezo1 channel may regulate beta cell biology and diabetes.

Reviewer #4:
Remarks to the Author:
This is a very intriguing and potentially important paper on a novel mechanism of ion transport in pancreatic beta cells. The authors have responded to prior critiques of their manuscript in a responsible manner, as many valid concerns were raised by the previous reviewers. However,

there are still issues with the paper unfortunately that need to be addressed.

1. Overall, the fact that knocking out Piezo1 leads to changes in gene expression, while interesting and a minor focus of the current paper does make the story more complex. The previous reviewers expressed concern about this and while the work with GsMx4 would seem to neutralize these concerns in the main, it would help if the authors could provide argument about differing time scales of the membrane protein knockout vs. change in expression of many genes? Perhaps this could be argued based on in vitro data where Piezo1 was silenced? This would address what I believe to be concerns about CACN and other ion channels being changed by silencing and what the relationship might be between KATP and Piezo1 channels. It also appears that in some examples shown, Piezo1 knockdown did reduce KCl-induced rises in Ca2+ (see Figs. 4A, 4H, 6F)

2. While there is no doubt that hypotonic conditions evokes insulin secretion via cell swelling, it seems hard to accept that sufficient beta cell swelling occurs in vivo in intact tissue like pancreas and isolated beta cells may be more sensitive to swelling than in vivo. This will likely be hard to prove one way or another and the shear stress experiments to my mind were not convincing: this must be damaging in many ways to cells as it is rather nonspecific. Plus, virtually all mammalian cells have volume regulated ion channels like SWELL1 and others, and the same could be true of PIEZO1? The finding that PIEZO1 is altered by hyperglycemia is interesting but cause and effect here is complex and not separable at present. Many genes are affected by plasma glucose, as the authors know.

3. The authors should confirm by western blots that in their knockouts and knockdowns/silencing that protein levels of Piezo1 are reduced.

4. It is a concern for whole animal studies that RIP-cre mice were used for recombination due to expression of cre in the brain, which is well known from work from Philipson and colleagues, as well as others. This point at least needs to be discussed.

5. It is somewhat puzzling that the effects of Piezo1 activation were ablated by diazoxide. While it will indeed hyperpolarize the cells due to KATP activation, this should also increase driving force activating on cationic flux through Piezo1. The only way this might occur is if Piezo1 is also voltage dependent such that sufficient hyperpolarization might prevent its activation?

6. The loss of first phase GSIS in perfused pancreas is confusing and its restoration by yoda is strange.

7. While the authors explain the persistence of attenuated electrical activity in their beta cell specific Piezo1 knockouts as being due to invasion of spiking from delta cells, the alternative which seems quite plausible is that there is enough depolarization occurring in these islets to provide excitatory input to the beta cells despite a lack of Piezo1. Without a way to silence the delta cells I think their explanation is inconclusive.

Reviewers' comments:

Reviewer #2 (Remarks to the Author):

Comment: The manuscript by Yingying Ye et al. has been improved with the revisions, which have addressed my previous concerns. The manuscript provides important mechanistic understanding of how the mechanosensitive channel Piezo1 influences beta-cell calcium entry and insulin secretion.

Response: We were pleased to see that this Reviewer felt that the earlier version had addressed his/her previous concerns and that the manuscript 'provides important mechanistic information'.

Reviewer #3 (Remarks to the Author):

Comment: However, the key evidence supporting Piezo1 as a mechanosenor and responding to beta cell swelling in glucose-stimulated insulin secretion is still largely missing.

Piezo1 channel is activated by lipid bilayer membrane tension. In contrast to intuition, mild hypotonic swelling in mammalian cells actually does not generate strong membrane tension because mammalian cells have membrane reserve and invaginations. So far, it has not been reported in the literature that Piezo1 channel is activated by hypotonic cell swelling. Considering glucose only induces very mild beta cells swelling (5-10%), the new claim that Piezo1 channel is activated under this condition requires evidence. The authors speculated that the Piezo1 currents were too small to be electrophysiologically recorded. It's not clear whether such experiments were tried. If that's the case, the authors should overexpress Piezo1 and then record under high glucose perfusion. If beta cell recording is technically difficult, recording in a heterologous expression system should be performed to provide at least some indirect evidence.

Response: We agree that this is an important aspect. We have addressed this by intracellular sucrose infusion. This approach was chosen because extracellular hypotonicity causes cell rupture. However, functionally, increasing the intracellular osmolarity should be the same as lowering the extracellular osmolarity. We show that 100 mM intracellular sucrose (100 mOsm; 25% hyperosmolarity relative to the outside) increases cell capacitance (=cell area) by 40% within 3 min (Fig. 3e). Although cells (including beta-cells) have invaginations, these would still be included in our measurements of cell capacitance (which detects vesicles as small as 30 nm). It is therefore extremely unlikely that the observed increases in cell capacitance can occur without a corresponding increase in cell tension leading to the activation of Piezo 1. Indeed, we document an increase in membrane conductance that is sensitive to the Piezo1 inhibitor GsMTx4 under these conditions (Fig. 3f). We also show – by a non-invasive imaging technique – that glucose stimulation (from 2.8 to 16.7 mM) leads to a 6% increase in cell surface area (Fig. 4a). Taken together, we feel these observations provide good evidence that cell swelling/increase membrane tension results in activation of Piezo1 in beta-cells.

Comment: In addition to the Piezo1 current, the mechanically-activated calcium influx is another important physiological readout. However, the calcium imaging experiments in the manuscript were performed in the bulk beta cells/islets over a longer time scale. Although they are conventional in the field, these large calcium signals are mainly due to the opening of VGCC, but not the proposed Piezo1-mediated calcium influx. This distinction is important and need to emphsized. Given the claim that Piezo1-mediated calcium influx induces membrane depolarization, it's critical to investigate the very initial VGCC-independent (using VGCC blocker?) calcium influx in much shorter time scale and single beta cell resolution.

Response: We are not quite sure what the Reviewer means. We are not claiming that Ca^{2+} influx through Piezo1 produces the observed increase in Ca^{2+}. Piezo1 is a non-selective cation-permeable ion channel and we propose that it gives rise to a depolarizing current and that this in turn leads to regenerative electrical activity that culminates in Ca^{2+} entry due to opening of voltage-gated Ca^{2+} channels. The 0.5 nS increase in membrane conductance due to Piezo1 (measured in response to yoda1 with millisecond resolution) is <10% of the 3 nS of the voltage-gated Ca^{2+} entry (Fig. 4o). We have reworded the text to specifically emphasize this aspect (see lines 195-200).

Comment: The relationship between Piezo1 and Swell1 channels is puzzling. They were proposed to be activated by cell swelling but cause membrane depolarization with completely different mechanisms: Piezo1-calcium (positive ion) influx; SWELL1-choride (negative ion) efflux. The authors

should discuss the potential reasons why no additive effect of knocking down both pathways is observed.

Response: We welcome the opportunity to discuss the interactions between Piezo1 and Swell1. As the Reviewer notes, the ionic selectivity is quite different (cations vs Cl^-). However, with the ionic gradients in beta-cells (especially relatively high intracellular Cl^-), activation of either channel will result in membrane depolarization. Importantly, they do not operate in isolation but against a background of K_{ATP} channels. The simplest explanation to the conundrum that the effects are not additive is that both Piezo1 and Swell1 have to open for the full effect and when either of them is ablated, activation of one of the channels is not sufficient to overcome the repolarizing influence of the K_{ATP} channels (ll. 330-339).

Comment: Accumulating evidence suggests that Piezo1 is a multi-function protein, which may regulate cellular function in non-conventional/mechanical ways. Indeed, the authors presented nice profiling data that Piezo1 knockdown changes the expression of hundreds of genes. These changes could at least in part explain the phenotype such as insulin secretion the authors observed. Therefore, it's important to discuss this and keep an open mind on how Piezo1 channel may regulate beta cell biology and diabetes.

Response: Indeed. However, these effects on gene transcription are likely to be long-term and are unlikely to explain the acute effects of pharmacological activation and inhibition. We do discuss the effects on gene transcription (ll. 290-292). Clearly, the observation that Piezo1 accumulates in the nuclei in T2D is highly important from a pathophysiological perspective and we think that this – as alluded to in the final paragraph of the Discussion - is the way forward (ll. 340-347).

Reviewer #4 (Remarks to the Author):

Comment: This is a very intriguing and potentially important paper on a novel mechanism of ion transport in pancreatic beta cells. The authors have responded to prior critiques of their manuscript in a responsible manner, as many valid concerns were raised by the previous reviewers. However, there are still issues with the paper unfortunately that need to be addressed.

Response: We thank this Reviewer for his/her comments that the manuscript is 'intriguing' and 'potentially important'. We have made a significant effort to address the 'issues' that [still] need to be addressed'.

Comment: Overall, the fact that knocking out Piezo1 leads to changes in gene expression, while interesting and a minor focus of the current paper does make the story more complex. The previous reviewers expressed concern about this and while the work with GsMx4 would seem to neutralize these concerns in the main, it would help if the authors could provide argument about differing time scales of the membrane protein knockout vs. change in expression of many genes? Perhaps this could be argued based on in vitro data where Piezo1 was silenced? This would address what I believe to be concerns about CACN and other ion channels being changed by silencing and what the relationship might be between KATP and Piezo1 channels. It also appears that in some examples shown, Piezo1 knockdown did reduce KCl-induced rises in Ca2+ (see Figs. 4A, 4H, 6F)

Response: The observation that Piezo 1 leads to changes in gene expression is interesting. We agree with the Reviewer that the biophysical and the transcriptional effects likely operate on different time scales. We have analyzed the impact of Piezo1 inhibition on depolarization-evoked $[Ca^{2+}]_i$ increases and observed no or small effects (see Fig. 1, below). The fact that tolbutamide-induced electrical activity in Piezo1-deficient beta-cells is similar to that in control beta-cells also militates against the idea that Piezo1 interferes with the expression/function of other channels. We have added a statement to this effect on ll. 290-292.

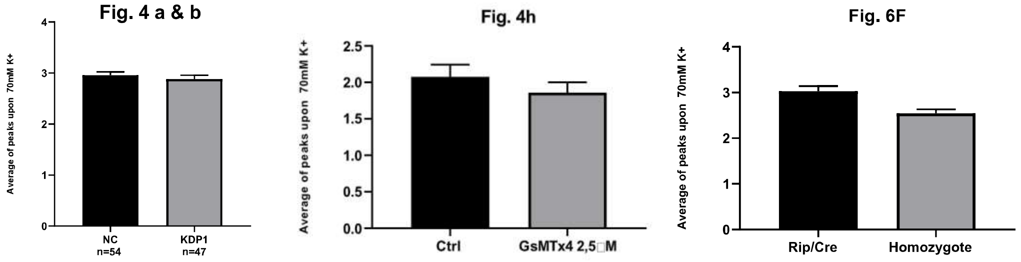

Comment: While there is no doubt that hypotonic conditions evokes insulin secretion via cell swelling, it seems hard to accept that sufficient beta cell swelling occurs in vivo in intact tissue like pancreas and isolated beta cells may be more sensitive to swelling than in vivo. This will likely be hard to prove one way or another and the shear stress experiments to my mind were not convincing: this must be damaging in many ways to cells as it is rather nonspecific. Plus, virtually all mammalian cells have volume regulated ion channels like SWELL1 and others, and the same could be true of PIEZO1? The finding that PIEZO1 is altered by hyperglycemia is interesting but cause and effect here is complex and not separable at present. Many genes are affected by plasma glucose, as the authors know.

Response: We believe that the data obtained with the blocker GsMTx4 using isolated islets and the perfused pancreas (Fig. 5d-e) in particular shows that Piezo1 plays an important role in the intact tissue.

The interaction between Swell1 (and other channels) and Piezo1 is of course an important aspect (also highlighted by Reviewer 3) and we have more extensively discussed this question in the concluding part of the manuscript (ll. 329-339).

With regard to the shear stress, we agree that the experimental protocol may not be optimal but it is one that have been used previously by others [1]. Naturally, shear stress will be damaging to the cells but we note that insulin secretion is only moderately increased when normalized to protein content (which was the same). The latter argues that cell damage and unspecific leakage of insulin is not a major factor.

Comment: The authors should confirm by western blots that in their knockouts and knockdowns/silencing that protein levels of Piezo1 are reduced.

Response: Piezo1 knockout has been confirmed by immunostaining which is visibly clear and obviously show the deletion of Piezo1 in beta-cells (Figure 6b). And Piezo1 knockdown efficiency has been tested on mRNA (by qPCR) and protein level (by western blot), please see Figure S4. Importantly, immunostaining of islets from Piezo1 KO mice illustrates the specificity of the antibody (now commented on; ll. 469-470)

Comment: It is a concern for whole animal studies that RIP-cre mice were used for recombination due to expression of cre in the brain, which is well known from work from Philipson and colleagues, as well as others. This point at least needs to be discussed.

Response: This important issue was raised in a previous round of reviews. The RIP-Cre mice we used in this study was generated by Dr. Pedro Herrera, which is known to have the most similar phenotype with C57BL/6 mice [2]. The study referred to shows that the effect on blood glucose in the RIP-cre mice is predominant in female, but very slight in male mice (Figure 1 in the ref paper [2]). In our study, we use male mice and observe a low degree of variation among all RIP-cre. This is now discussed in the revised manuscript ll. 232-235. We have also compared blood glucose excursions after IPGTT between RIP-cre and floxed Piezo1 mice, without any noticeable major differences (Fig. S7b).

Comment: It is somewhat puzzling that the effects of Piezo1 activation were ablated by diazoxide. While it will indeed hyperpolarize the cells due to KATP activation, this should also increase driving force activating on cationic flux through Piezo1. The only way this might occur is if Piezo1 is also voltage dependent such that sufficient hyperpolarization might prevent its activation?

Response: Electrophysiologically this is not puzzling at all! The membrane potential of the beta-cell (and thus electrical activity) is determined by a balance between depolarizing and repolarizing conductances. Among the latter, the K_{ATP} channels are by far the most important. Small depolarizing conductances will only be able to affect the membrane potential sufficiently to induce electrical activity when K_{ATP} channel activity is strongly reduced. In the presence of diazoxide, K_{ATP} channel activity is so large that Piezo1 (or any other conductance) is unable to evoke electrical activity. This concept is a cornerstone of beta-cell electrophysiology and explains why the insulinotropic effects of amino acids, muscarinic agonists and incretin hormones are largely restricted to normo- and hyperglycemic conditions. The ability of diazoxide to prevent the stimulatory effect of yoda1 does not have any implications in terms of an intrinsic voltage dependence of Piezo1. We now discuss this in some detail in the revised version of the manuscript (ll. 310-325).

Comment: The loss of first phase GSIS in perfused pancreas is confusing and its restoration by yoda is strange.

Response: Sadly, yoda1 had to be dissolved in DMSO and this – inexplicably – appeared to interfere with the kinetics of glucose-induced insulin secretion. Although it is clear that yoda1 has an effect, it may seem odd that the control response is different in this series of experiments and we have therefore decided to omit these data.

Comment: While the authors explain the persistence of attenuated electrical activity in their beta cell specific Piezo1 knockouts as being due to invasion of spiking from delta cells, the alternative which seems quite plausible is that there is enough depolarization occurring in these islets to provide excitatory input to the beta cells despite a lack of Piezo1. Without a way to silence the delta cells I think their explanation is inconclusive.

Response: This proposal was based on a limited number of experiments. We have subsequently had more mice and this has allowed us to explore the impact of ablating Piezo1 in a statistically more rigorous way and it is clear that beta-cells remain capable of generating electrical activity in the absence of this channel but that the depolarization produced by glucose is impaired. The new data allow us to quantitate the relative contribution of Piezo1 and the other conductances in the beta-cell responses to glucose. Thus, Piezo1 contributes to the glucose-induced membrane depolarizations but it is not the sole depolarizing conductance (see ll. 271-274). We believe the new data and the revised model is better agreement with the published literature.

1. Deivasikamani, V., et al., *Piezo1 channel activation mimics high glucose as a stimulator of insulin release.* Sci Rep, 2019. **9**(1): p. 16876.
2. Lee, J.Y., et al., *RIP-Cre revisited, evidence for impairments of pancreatic beta-cell function.* J Biol Chem, 2006. **281**(5): p. 2649-53.

Reviewers' Comments:

Reviewer #3:
Remarks to the Author:
The authors are responsive to the previous comments made by the reviewers, and the manuscript was improved. However, there are still a few issues that are mainly related to the new data and require further attention.

1. Piezo1 channel is activated by mechanical forces applied to cells, such as poking, membrane stretch, and shear stress. However, hypotonic swelling is not known to directly activate Piezo1, even in cells (such as HEK293) overexpressing this channel. In the revision, the authors presented new data in Fig. 3f that intracellular hypertonicity (which induces cell swelling) increases membrane conductance. The description of this important experiment is rather brief and it's implied that this conductance/current is mediated by Piezo1: "To confirm that the inward current carried by PIEZO1 depolarizes INS-1 832/13 cells,". This is a critical set of new data and needs to expand and presented clearly. Instead of "ratio of conductance", it's better to present the whole-cell current at both positive and negative mV over time. So, the kinetics and rectification of the current can be examined. Intracellular hypertonicity also activates Swell1, a large chloride current. According to the solutions (chloride-based) described in the methods, Swell1 current should be very prominent which also reverse at 0 mV. It's surprising that GsMTx4, not a Swell1 inhibitor, completely abolished this conductance. GsMTx4 is not specific to Piezo1 either and is known to block other ion channels (i.e. TRP channels). So, knockdown Piezo1 and Swell1 are better experiments here to establish the role of these two channels in mediating this cell swelling-induced current.

2. Both Figures 1 and 2 are based on Piezo1 immunostaining. So, the specificity of the Piezo1 antibody in IF is critical. Fig. 6b is the strongest evidence the authors presented to support its specificity. But, there is only one small insulin-positive cell in the cKO culture. It would be better to show an islet section with many beta cells as in Fig. S1c (whole and inserts).

3. Please cite and maybe discuss another study (posted in Biorxiv in 2018, published in 2019) on the effects of Piezo1 activation on insulin secretion: "The Piezo1 channel activation mimics high glucose as a stimulator of insulin". It complements some of the conclusions in the current manuscript.

Reviewer #4:
Remarks to the Author:
Reexamination of the revised manuscript and the response to the reviewers supports the importance of the manuscript and the careful responses provided by the authors. The authors argue convincingly that Piezo1 contributes to the control of glucose induced insulin secretion.

Reviewer 3:

We thank Reviewer 3 for taking their time to comment on our manuscript again.

Comment: The authors are responsive to the previous comments made by the reviewers, and the manuscript was improved. However, there are still a few issues that are mainly related to the new data and require further attention.

Response: We appreciate the comment that we have responded to the Reviewers' earlier comments and that the revised manuscript 'was improved'. We have dealt with the 3 issues Reviewer 3 highlights in their report as follows:

Comment 1[a]: Piezo1 channel is activated by mechanical forces applied to cells, such as poking, membrane stretch, and shear stress. However, hypotonic swelling is not known to directly activate Piezo1, even in cells (such as HEK293) overexpressing this channel. In the revision, the authors presented new data in Fig. 3f that intracellular hypertonicity (which induces cell swelling) increases membrane conductance. The description of this important experiment is rather brief and it's implied that this conductance/current is mediated by Piezo1: "To confirm that the inward current carried by PIEZO1 depolarizes INS-1 832/13 cells,". This is a critical set of new data and needs to expand and presented clearly. Instead of "ratio of conductance", it's better to present the whole-cell current at both positive and negative mV over time. So, the kinetics and rectification of the current can be examined. Intracellular hypertonicity also activates Swell1, a large chloride current.

Response: We agree. The experiments in the INS-1 832/13 cells were challenging and we eventually decided to use beta-cells in intact pancreatic islets for these experiments. We used the standard whole-cell configuration for these experiments. To prevent insulin secretion (exocytosis), the experiments were done with fairly high intracellular EGTA concentrations (to chelate calcium) and at room temperature (a condition known to strongly inhibit secretion in beta-cells). Thus, the increase in cell size (measured by membrane capacitance) we observe is unlikely to reflect massive exocytosis but rather stretching/unfolding of the membrane. We respectfully point out that our measurements indicate that hypotonicity does indeed activate PIEZO1 (Fig. 3). Hypotonicity has previously been reported to activate Piezo1 in insulin secreting cells (see PMID: 31727906). We now show that application of intracellular sucrose (to mimic the effects of extracellular hypotonicity and the impact of intracellular accumulation of glucose metabolites) leads to a time-dependent activation of a small outwardly rectifying GsMTx4-sensitive current with a reversal potential at 0 mV (Fig. 4p-q). The 'raw' traces used to calculate the net swelling-induced current are shown in Fig. S7a. The time dependence of the activation of the difference current is shown in Fig. S7b.

Comment 1[b]: According to the solutions (chloride-based) described in the methods, Swell1 current should be very prominent which also reverse at 0 mV. It's surprising that GsMTx4, not a Swell1 inhibitor, completely abolished this conductance. GsMTx4 is not

specific to Piezo1 either and is known to block other ion channels (i.e. TRP channels). So, knockdown Piezo1 and Swell1 are better experiments here to establish the role of these two channels in mediating this cell swelling-induced current.

Response: We found that the current induced by hypotonicity (intracellular sucrose) was strongly inhibited by GsMTx4 (Fig.4q). We agree that knockdown experiment is a better way of determining the selectivity GsMTx4 and we now demonstrate that knockdown of Piezo1 in INS-1 832/13 cells and GsMTx4 similarly reduced the capacity of glucose to increase intracellular calcium and that the effects of the two approaches were not additive. Thus, we believe it is justifiable to conclude that GsMTx4 in INS-1 832/13 cells and under our experimental conditions is a selective inhibitor of Piezo1. Of note, neither knockdown of Piezo1 nor GsMTx4-induced inhibition of the current abolished the response to glucose, illustrating the existence of additional depolarizing currents (including – but not limited to -Swell1). This is now acknowledged.

Comment 2. Both Figures 1 and 2 are based on Piezo1 immunostaining. So, the specificity of the Piezo1 antibody in IF is critical. Fig. 6b is the strongest evidence the authors presented to support its specificity. But, there is only one small insulin-positive cell in the cKO culture. It would be better to show an islet section with many beta cells as in Fig. S1c (whole and inserts).

Response: Indeed. We now provide more extensive analysis of these data (based on 28 control cells and 12 KO cells.

3. Please cite and maybe discuss another study (posted in Biorxiv in 2018, published in 2019) on the effects of Piezo1 activation on insulin secretion: "The Piezo1 channel activation mimics high glucose as a stimulator of insulin". It complements some of the conclusions in the current manuscript.

Response: We agree. Indeed, this paper was cited in the revised version and we now also refer to it in the context hypotonicity-induced current. Our study builds on this earlier study and we believe that they together provide strong evidence for an important role of Piezo1 in insulin secretion.

Reviewer 4:

Reexamination of the revised manuscript and the response to the reviewers supports the importance of the manuscript and the careful responses provided by the authors.

We thank the Reviewer for their re-examination of our manuscript.

The authors argue convincingly that Piezo1 contributes to the control of glucose induced insulin secretion.

Thank you.

Reviewers' Comments:

Reviewer #3:
Remarks to the Author:
The authors are responsive to the previous comments made by the reviewers, and the manuscript has been improved for publication.
